# Supplementary material for: Antioxidant and antidiabetic effects of Angylocalyx oligophyllus leaves aqueous extract in pregnant diabetic rats: Feto-maternal repercussions
Source: PLoS One. 2025 Nov 14;20(11):e0334166. doi: 10.1371/journal.pone.0334166 (PMC12617868; doi:10.1371/journal.pone.0334166)
Supplement: S1 Data — (PDF) [file pone.0334166.s001.pdf]

QUANTITATIVE PHYTOCHEMICAL DATA

TOTAL POLYPHENOLS

Concentration (µg/mL)	0	5	10	15	20	25
OD(nm)	-0,096	0,024	0,21	0,358	0,608	0,808

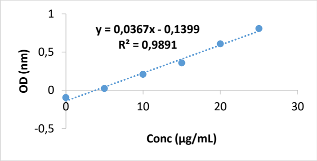

TOTAL FLAVONOIDS

Concentration (µg/mL)	0	50	100	150	200	250
OD(nm)	0,114	0,137	0,134	0,166	0,195	0,202

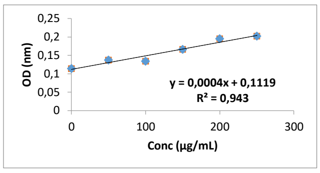

DATA OF THE *IN VITRO* TESTS

IN VITRO ALPHA AMYLASE TEST

Concentration (mg/mL)	Acarbose			*A. oligophyllus*		
**6,125**	13,81	14,13	12,75	8,33	8,19	7,41
**12,5**	24,57	23,38	22,41	13,81	17,21	11,69
**25**	51,31	50,99	50,99	34,88	41,14	35,20
**50**	86,75	84,17	75,89	96,55	92,31	87,34

IN VITRO ANTIOXYDANT TESTS

ABTS+

Concentration (µg/mL)	TROLOX					*A. oligophyllus*				
**1**	30,30	11,83	32,61	46,18	15,87	19,05	27,99	28,14	22,37	32,18
**3**	41,56	38,96	33,19	18,04	24,10	15,15	19,62	23,81	28,72	26,70
**10**	90,76	61,47	101,59	100,58	78,64	67,39	44,44	54,69	22,08	52,09
**30**	69,99	102,45	101,73	72,15	100,72	91,92	88,02	81,10	88,60	92,21
**100**	79,65	76,77	99,57	96,39	86,58	107,07	103,17	107,07	106,06	106,64
**300**	74,17	85,14	94,81	94,23	92,93	101,01	93,94	104,04	99,13	89,61
**700**	97,40	100,58	100,72			92,50	94,44	97,76		
**1000**	92,21	93,29	93,80			95,89	95,96	96,39		

HO·

Concentration (µg/mL)	TROLOX			*A. oligophyllus*		
**1**	-2,94	5,18	15,65	7,19	-3,13	3,63
**3**	11,86	21,99	21,52	3,98	8,15	11,75
**10**	16,89	23,26	23,11	12,36	7,77	14,57
**30**	18,24	23,49	23,80	16,92	12,94	25,50
**100**	25,58	37,56	49,92	18,89	29,33	20,44
**300**	56,30	42,16	35,86	26,93	23,26	9,78
**700**	62,60	48,57	63,14	27,01	14,53	9,54
**1000**	62,29	67,12	75,73	25,62	31,80	24,77

DPPH

Concentration (µg/mL)	Vit C			A. oligophyllus		
**1**	9,60	-0,12	37,79	7,17	-1,34	14,46
**3**	27,34	44,59	44,59	-2,79	-22,72	15,67
**10**	88,34	91,74	90,77	7,17	-0,36	-12,52
**30**	100,49	91,25	96,11	27,58	32,93	32,69
**100**	106,32	96,84	89,79	84,45	91,74	94,17
**300**	99,03	100,49	99,51	104,37	117,98	103,16
**700**	90,77	96,11	91,74	133,29	129,40	125,03
**1000**	94,17	93,20	114,09	131,35	122,84	127,70

FRAP

Concentration (µg/mL)	VITC			A.oligophyllus		
**1**	0,41	0,36	0,43	0,14	0,14	0,14
**3**	0,88	1,24	1,20	0,05	0,05	0,05
**10**	2,59	2,69	2,88	0,22	0,18	0,18
**30**	2,94	2,91	2,82	0,43	0,37	0,40
**100**	3,58	3,18	3,18	1,03	1,23	0,91
**300**	2,89	3,08	2,92	3,19	3,17	3,17
**700**	2,77	2,77	2,77	3,27	2,81	3,27
**1000**	2,56	2,56	2,56	3,51	2,86	3,51

DATA OF MATERNAL PARAMETERS

BODY MASS

DAYS	5	10	15	20
NC	151,17	195,20	204,60	215,00
	153,17	207,00	206,60	216,50
	170,83	209,80	219,20	228,50
	164,83	210,60	222,00	234,80
	153,67	204,80	208,00	217,75
	145,83	185,60	190,60	197,50
	158,67	199,80	208,80	218,75
	150,37	187,90	194,80	199,89
	162,00	204,30	217,90	242,00

DAYS	5	10	15	20
DC	155,83	195,60	199,00	202,20
	154,50	187,40	200,60	195,50
	156,17	195,60	199,00	202,20
	174,33*	220,80*	225,60*	231,00*
	142,40	192,00	199,40	201,00
	152,17	186,40	198,20	210,00

DAYS	5	10	15	20
Gli	147,83	184,60	189,20	192,75
	159,00	191,00	185,80	208,75
	174,83	215,00	208,20	208,50
	146,83	192,00	194,80	199,25
	164,67	204,40	214,00	217,00

DAYS	5	10	15	20
AoAE50	150,00	174,60	184,00	196,25
	150,17*	182,00*	185,00*	193,25*
	151,17	188,00	198,00	206,00
	144,17*	179,00*	190,60*	189,50*
	160,67	196,00	203,40	208,25
	162,33	205,40	219,60	228,75
	150,53	184,05	193,05	199,12
	156,10	199,87	207,05	214,43

DAYS	5	10	15	20
AoAE100	158,83	198,80	204,00	211,00
	142,67*	174,80*	166,75*	172,00*
	169,50	209,80	211,10	218,60
	156,55	188,50	191,45	189,06
	170,23	207,17	213,85	222,63

DAYS	5	10	15	20
AoAE200	163,67	208,80	222,00	250,50
	163,00	209,00	217,00	253,50
	150,50	190,20	200,80	209,33
	172,83	209,80	224,60	220,50
	170,50	208,40	214,80	222,50
	153,17	185,40	202,40	211,67
	159,67	189,80	206,20	219,60

RELATIVE ORGAN MASSES

LIVER

NC	DC	Glib	AoAE50	AoAE100	AoAE200
3,45	3,14	3,95	3,50	2,23	3,71
3,69	3,70	4,62	3,52	4,14	3,17
2,56	4,33	3,62	3,66	3,34	4,68*
3,20	4,06	3,78	3,30	2,97	3,44
2,53	3,15	4,97*	2,75	3,61	4,40
2,89	3,52		3,35		4,54
2,42			3,51		3,84
2,30					

KIDNEY

NC	DC	Glib	AoAE50	AoAE100	AoAE200
0,52	0,62	0,73	0,51	0,35	0,71
0,53	0,64	0,60	0,48	0,59	0,53
0,43	0,72	0,61	0,64	0,50	0,84
0,53	0,67	0,64	0,62	0,44	0,55
0,35	0,46	0,73	0,43	0,54	0,73
0,48	0,75		0,51		0,61
0,44			0,53		0,79
0,36					

PANCREAS

NC	DC	Glib	AoAE50	AoAE100	AoAE200
0,12	0,16	0,21	0,15	0,10	0,31
0,15	0,14	0,16	0,14	0,15	0,20
0,14	0,15	0,18	0,18	0,19	0,16
0,14	0,10	0,17	0,18	0,17	0,16
0,15	0,12	0,17	0,09	0,12	0,15
0,13	0,20		0,13		0,28
0,10			0,16		0,16
0,13					

HEART

NC	DC	Glib	AoAE50	AoAE100	AoAE200
0,27	0,28	0,29	0,30	0,17	0,34
0,25	0,28	0,26	0,24	0,25	0,28
0,29	0,25	0,22	0,30	0,29	0,29
0,27	0,29	0,26	0,28	0,28	0,41
0,25	0,28	0,30	0,27	0,27	0,27
0,25	0,30		0,27		0,27
0,27			0,28		0,33
0,21					

AORTA

NC	DC	Glib	AoAE50	AoAE100	AoAE200
0,04	0,03	0,03	0,06	0,02	0,02
0,03	0,08	0,04	0,04	0,04	0,02
0,04	0,02	0,06	0,02	0,04	0,02
0,03	0,03	0,03	0,05	0,05	0,06
0,03	0,08	0,06	0,06	0,02	0,04
0,04	0,02		0,02		0,02
0,04			0,02		0,05
0,03					

SPLEEN

NC	DC	Glib	AoAE50	AoAE100	AoAE200
0,26	0,11	0,23	0,17	0,17	0,32
0,60*	0,22	0,64	0,18	0,17	0,17
0,13	0,32	0,16	0,23	0,31	0,16
0,16	0,12	0,36	0,17	0,26	0,29
0,16	0,46	1,00	0,12		0,26
0,16	0,43		0,38		0,50
0,14			0,34		0,53
0,11					

BRAIN

NC	DC	Glib	AoAE50	AoAE100	AoAE200
0,73	0,86	0,99	0,88	0,51	0,79
0,69	0,75	0,80	0,75	0,82	0,86
0,82	0,79	0,78	0,30	0,75	0,84
0,42	0,85	0,86	0,79	0,81	0,81
0,68	0,77	0,81	0,75	0,70	0,85
0,83	0,78		0,67		0,73
0,78			0,76		0,82
0,71					

ADRENAL GLAND

NC	DC	Glib	AoAE50	AoAE100	AoAE200
0,03	0,03	0,04	0,03	0,02	0,03
0,02	0,03	0,04	0,02	0,05	0,02
0,03	0,03	0,04	0,02	0,03	0,03
0,03	0,05	0,04	0,03	0,03	0,05
0,02	0,03	0,05	0,03	0,04	0,02
0,03	0,04		0,03		0,05
0,05			0,02		0,04
0,03					

ABDOMINAL FAT

NC	DC	Glib	AoAE50	AoAE100	AoAE200
1,41	0,00	0,00	0,30	1,16	0,22
1,56	0,23	1,05	0,45	0,15	0,26
1,19	0,43	0,00	0,43	1,55	1,22
1,64	0,02	0,39	0,24	1,23	0,10
0,72	0,54	0,42	0,85	0,66	1,00
2,06	0,16		2,03		0,40
0,43			1,48		0,45
0,43					

PERIOVARIAN FAT

NC	DC	Glib	AoAE50	AoAE100	AoAE200
1,09	0,00	0,00	1,63	0,61	0,15*
1,59	0,60	0,90	1,21	0,26	0,75
2,15*	0,33	0,20	0,86	0,80	1,60
1,59	0,02	0,40	0,53	0,70	1,92
1,32	1,92*	1,13	2,59*	0,64	0,70
0,84*	0,46		1,60		0,58
1,07			1,50		1,08
1,04					

OVARIES

NC	DC	Glib	AoAE50	AoAE100	AoAE200
0,03	0,04	0,05	0,05	0,03	0,04
0,015*	0,04	0,04	0,03	0,04	0,04
0,04	0,03	0,04	0,04	0,03	0,04
0,04	0,04	0,04	0,04	0,03	0,03
0,026*	0,05	0,05	0,03	0,04	0,04
0,04	0,04		0,04		0,03
0,04			0,05		0,02
0,03					

GLYCEMIA

Group	DAYS 1	5	10	15	20
NC	121	108	80	82	84
	117	81	100	108	76
	70	97	74	71	76
	115	103	86	97	102
	110	115	84	88	100
	113	91	99	105	82

Group	DAYS 1	5	10	15	20
DC	220	385	319	461	407
	445	396	288	235	430
	456	388	515	410	390
	374	390	374	369	399
	257	396	305	386	360

Group	DAYS 1	5	10	15	20
Glib	486	512	305	260	236
	221	423	326		
	402	361	426	519	313
	329	180	416	405	236
	392	320	293	256	313

Group	DAYS 1	5	10	15	20
AoAE50	433	335	383	328	221
	327	316	343	35	350
	366	297	257	366	217
	393	370	380	202	119
	255	384	380	270	251
	212	125	159	160	149

Group	DAYS 1	5	10	15	20
AoAE100	257	250	312	354	292
	463	368	278	400	330
	365	75	320	90	56
	385	275	220	320	307
	231	284	333	330	330

Group	DAYS 1	5	10	15	20
AoAE200	301	292	357	370	165
	342	200	271	170	123
	364	263	252	250	216
	381	375	386	345	385
	277	325	362	262	216
	238	181	65	125	111

ORAL GLUCOSE TOLERANCE TEST (OGTT)

GROUP	Time (Min) 0	30	60	120
NC	100,00	131,75	130,16	95,24
	100,00	104,40	102,20	102,20
	100,00	144,12	132,35	111,76
	100,00	147,37	128,07	114,04
	100,00	144,12	125,00	105,88
	100,00	134,25	123,00	106,20

GROUP	Time (Min) 0	30	60	120
DC	100,00	212,24	176,33	152,65
	100,00	224,77	187,16	117,89
	100,00	171,24	145,15	111,04
	100,00	182,48	153,83	116,41
	100,00	198,55	166,75	125,60

GROUP	Time (Min) 0	30	60	120
Glib	*100,00**	*230,47**	*157,81**	*146,10**
	100,00	149,36	133,01	84,62
	100,00	258,75	205,00	95,00
	100,00	264,56	216,46	98,73
	100,00	225,78	178,78	109,84

GROUP	Time (Min) 0	30	60	120
AoAE50	100,00	159,32	149,05	110,65
	100,00	140,80	130,06	96,63
	100,00	178,47	199,31	113,89
	100,00	222,15	196,64	87,92
	100,00	210,28	199,02	126,06
	100,00	184,35	176,29	108,13

GROUP	Time (Min) 0	30	60	120
AoAE100	100,00	127,84	115,98	106,70
	100,00	192,63	173,73	110,14
	100,00	217,72	219,62	112,66
	100,00	173,99	161,43	109,87
	100,00	178,04	167,69	109,84

GROUP	Time (Min) 0	30	60	120
AoAE200	100,00	120,80	115,79	93,23
	100,00	177,09	139,32	94,74
	100,00	140,80	130,06	96,63
	100,00	176,95	162,71	123,05
	100,00	153,55	134,97	101,92
	100,00	153,22	138,15	102,11

AIREA UNDER CURVE (AUC)

NC	DC	Glib	AoAE50	AoAE100	AoAE200
359,50	514,90		413,70	347,20	333,20
307,70	520,90	374,70	369,20	471,40	413,80
382,40	421,90	561,30	484,70	543,70	369,20
382,50	444,50	580,40	512,80	440,40	451,20
372,10	478,10	509,50	522,30	450,70	389,50
360,40			464,70		392,40

INSULIN TOLERANCE TEST (ITT)

Group	Time (Min) 0	30	60
NC	100,00	57,83	65,06
	100,00	31,58	30,26
	100,00	32,97	25,27
	100,00	52,94	51,96
	100,00	55,70	34,96
	100,00	40,08	47,26

Group	Time (Min) 0	30	60
DC	100,00	31,25	15,06
	100,00	43,92	13,40
	100,00	53,81	20,55
	100,00	44,01	16,63
	100,00	43,25	16,41

Group	Time (Min) 0	30	60
Glib	100,00	50,65	64,94
	100,00	53,25	21,00
	100,00	59,35	47,83
	100,00	56,09	37,82
	100,00	54,83	42,90

Group	Time (Min) 0	30	60
AoAE50	100,00	30,82	11,19
	100,00	58,33	19,12
	100,00	21,39	10,96
	100,00	36,13	26,05
	100,00	34,74	15,15
	100,00	38,60	18,51

Group	Time (Min) 0	30	60
AoAE100	100,00	69,64	33,93
	100,00	9,88	15,02
	100,00	70,53	40,05
	100,00	46,77	30,46
	100,00	49,21	29,87

Group	Time (Min) 0	30	60
AoAE200	100,00	40,17	28,93
	100,00	27,23	21,78
	100,00	45,72	17,36
	100,00	39,75	22,36
	100,00	36,34	21,72
	100,00	40,11	23,50

AIREA UNDER CURVE (AUC)

NC	DC	Glib	AoAE50	AoAE100	AoAE200
140,40	88,78	133,10	86,42	136,60	104,60
96,71	100,60	113,80	117,90	67,39	88,12
95,60	114,10	133,30	76,87	140,60	104,40
128,90	102,30	125,00	99,16	112,00	100,90
123,20	101,50	126,30	92,32	114,10	97,20
113,70			97,86		101,90

AST, ALT, AST/ALT and TOTAL PROTEINS

AST

NC	DC	Glib	*AoAE50*	*AoAE100*	*AoAE200*
110,58	195,29	237,77	142,01	182,15	188,52
77,08	216,00	234,04	128,62	196,45	180,00
151,90	325,01	131,29	188,83	195,68	162,46
130,69	330,93	139,44	101,01	138,67	107,54
59,82	339,66	128,62	161,71	187,11	110,00

ALAT

NC	DC	Glib	*AoAE50*	*AoAE100*	*AoAE200*
47,96	73,12	15,51	28,02	33,67	39,72
19,10	63,11	39,05	21,43	34,24	46,71
35,57	30,33	20,09	34,01	32,79	35,36
27,65	40,65	47,31	19,70	18,48	45,72
20,19	77,01	15,58	62,07	45,32	36,01

ASAT/ALAT

NC	DC	Glib	*AoAE50*	*AoAE100*	*AoAE200*
2,31	2,67	15,33	5,07	5,41	4,75
4,04	3,42	5,99	6,00	5,74	3,85
4,27	10,72	6,54	5,55	5,97	4,59
4,73	8,14	2,95	5,13	7,50	2,35
2,96	4,41	8,25	2,61	4,13	3,05

TOTAL PROTEINS

NC	DC	Glib	*AoAE50*	*AoAE100*	*AoAE200*
2,89	3,79	3,28	2,71	2,58	5,20
3,40	2,82	2,64	3,74	3,67	4,27
2,87	2,37	4,58	2,71	3,90	4,07
3,97	3,12	3,40	4,27	3,32	3,72
3,61	3,25	4,13	4,35	3,86	4,81

LIIPID PROFILE

TRIGLYCERIDES

NC	DC	Glib	*AoAE50*	*AoAE100*	*AoAE200*
108,67	178,18	126,00	82,67	142,00	152,00
154,67	201,82	210,00	137,33	124,00	127,33
132,67	163,64	161,33	118,00	124,00	146,67
115,33	198,18	156,00	132,67	124,67	159,33
154,00	220,91	114,00	144,67	*30,00**	*64,67**

TOTAL CHOLESTEROL

NC	DC	Glib	*AoAE50*	*AoAE100*	*AoAE200*
53,48	72,05	57,57	55,34	42,37	64,25
79,11	124,05	56,08	70,19	40,85	60,17
54,22	128,88	67,97	87,65	42,71	55,34
63,88	103,25	59,61	43,45	93,22	42,71
75,39	116,77	74,92	92,11	97,25	78,77

HDL-CHOLESTEROL

NC	DC	Glib	*AoAE50*	*AoAE100*	*AoAE200*
11,76	*19,65**	10,89	16,40	24,66	30,79
*9,89**	7,26	16,27	10,14	23,28	29,54
27,16	7,01	16,27	18,90	16,65	22,65
12,52	7,51	12,77	11,26	10,39	21,65
17,18	7,38	17,02	16,00	15,02	*10,86**

LDL-CHOLESTEROL

NC	DC	Glib	*AoAE50*	*AoAE100*	*AoAE200*
19,98		21,48	22,41	10,69	3,06
	76,43	2,19	32,59	7,22	5,16
0,53	89,14	19,43	45,15	1,27	3,35
28,30	56,10	15,64	5,66	57,90	10,81
27,41	65,21	35,10	47,18		

LIVER OXYDATIVE STRESS PARAMETERS

MDA

NC	DC	Gli	AoAE50	AoAE100	AoAE200
0,007	0,010	0,004	0,008	0,003	0,005
0,008	0,021	0,016	0,009	0,003	0,005
0,003	0,015	0,012	0,008	0,010	0,008
0,009	0,027	0,006	0,007	0,008	0,009
0,007	0,017	0,008	0,008	0,006	0,008

SOD

NC	DC	Gli	AoAE50	AoAE100	AoAE200
0,325	2,602	1,382	0,650	0,244	0,813
0,813	2,276	1,707	0,813	0,244	0,894
0,081	1,707	1,870	0,813	1,301	0,407
0,488	1,382	1,768	0,813	0,569	0,813
0,407	1,821	1,682	0,407	0,797	0,906

CAT

NC	DC	Gli	AoAE50	AoAE100	AoAE200
0,249	0,519	0,233	0,278	0,426	0,413
0,234	0,903	0,230	0,576	0,291	0,203
0,447	0,792	0,531	0,442	0,356	0,356
0,378	1,339	0,315	0,525	0,462	0,392
0,327	0,888	0,449	0,480	0,423	0,448

GSH

NC	DC	Gli	AoAE50	AoAE100	AoAE200
0,065	0,100	0,125	0,075	0,055	0,051
0,065	0,142	0,076	0,077	0,040	0,068
0,063	0,116	0,125	0,096	0,055	0,023
0,103	0,103	0,076	0,058	0,040	0,050
0,087	0,125	0,077	0,068	0,053	0,060

HISTOMORPHOMETRIC DATA OF THE PANCREAS

NC	DC	Glib	AoAE50	AoAE100	AoAE200
1728,72	778,72	1217,38	1304,50	1079,38	1128,32
1304,50	704,50	1072,32	905,24	991,15	1212,24
905,24	905,24	1541,11	1541,11	1234,13	1514,37
958,75	758,75	1236,70	1236,70	1264,24	1004,10
1160,74	760,74	976,63	1204,37	1604,15	1284,35

REPRODUCTION PERFORMANCE

% OF GESTATION

PARAMETERS OF FEMALE RATS	NC	DC	Glib	*AoAE50*	*AoAE100*	*AoAE200*
Number of mated female rats	10	20	10	10	10	10
Number of pregnant female rats	9	6	5	8	5	7
Number of pseudopregnant female rats	1	14	5	2	5	3
**% of gestation**	**90**	**30**	**50**	**80**	**50**	**70**

CORPORA LUTEA

NC	DC	Glib	*AoAE50*	*AoAE100*	*AoAE200*
36	19	37	24	33	23
23	16	27	25	40	20
40	31	32	36	28	18
36	18	33	30	32	19
21	16	40	23	32	37
36	22		28		36
41			37		12
32					

IMPLANTATION SITE

NC	DC	Glib	*AoAE50*	*AoAE100*	*AoAE200*
7	9	9	9	8	6
9	6	6	8	7	9
12	9	8	9	9	11
14	9	8	8	9	10
9	7	8	10	5	8
8	8		8		10
8			7		9
7					

PRE-IMPLANTATION LOSS (%)

NC	DC	Gli	AoAE 50	AoAE 100	AoAE 200
80,56	42,11	75,68	62,50	70,76	76,00
60,87	43,75	77,78	68,00	77,40	59,09
70,00	80,65	75,00	75,00	58,96	45,00
61,11	50,00	75,76	73,33	66,88	45,38
57,14	43,75	80,00	56,52	73,38	84,49
77,78	68,18		71,43		75,68
80,49			81,08		64,27
78,13					

POST-IMPLANTATION LOSS

NC	DC	Glib	*AoAE50*	*AoAE100*	*AoAE200*
0	9,0909091	0	0	0	0
0	11,111111	16,666667	0	14,285714	22,222222
8,3333333	0	12,5	0	0	18,181818
0	0	12,5	12,5	0	0
0	11,111111	12,5	0	0	25
0	100		0		0
0			0		0
0					

DEAD FETUSES

NC	DC	Glib	*AoAE50*	*AoAE100*	*AoAE200*
0	0	0	0	0	0
0	0	0	0	0	0
0	0	0	0	0	0
0	0	0	0	0	0
0	0	0	0	0	0
0	**8**		0		0
0			0		0
0					

FETAL SURVIVAL LEVEL

NC	DC	Glib	*AoAE50*	*AoAE100*	*AoAE200*
100,00	90,91	100,00	88,89	100,00	100,00
100,00	88,89	83,33	87,50	85,71	77,78
83,33	100,00	87,50	100,00	100,00	81,82
71,43	100,00	87,50	87,50	100,00	100,00
88,89	100,00	100,00	100,00	100,00	75,00
87,50	100,00		100,00		110,00
87,50			100,00		77,78
142,86					

GESTATIONAL INDEX (%)

NC	DC	Glib	*AoAE50*	*AoAE100*	*AoAE200*
70	50	75	80	80	60
90	40	52	70	60	70
100	30	68	90	90	90
100	45	68	70	90	100
80	45	70	100	50	60
70	35		80		110
70			70		70
100					

RESORPTION RATE

NC	DC	Glib	*AoAE50*	*AoAE100*	*AoAE200*
0	0	0	0	0	0
0	0	0	0	0	0
0	0	0	0	0	0
0	**1**	**1**	0	0	0
0	0	**1**	0	0	0
0	8		0		0
0			0		**1**
**1**					

UTERINE HORN MASS (g)

NC	DC	Glib	*AoAE50*	*AoAE100*	*AoAE200*
32,10	32,02	26,87	39,27	24,96	27,52
*27,889**	34,35	26,60	35,55	39,05	36,96
42,52	17,79	22,35	36,41	24,74	35,59
45,66	35,72	25,27	30,75	29,58	34,30
42,83	16,04	22,41	51,78	27,47	25,73
30,87	27,18		40,34		*20,26**
42,25			*22,63**		32,08
36,52					

ESTRADIOL-PROGESTERONE

β-OESTRADIOL

NC	*DC*	*Gli*	*AoAE50*	*AoAE100*	*AoAE200*
376,35	266,85	292,51	362,90	339,20	318,17
332,00	266,80	387,15	310,03	320,69	337,52
326,58	256,75	226,47	435,99	326,16	318,17
305,50	198,28	305,97	299,20	323,22	337,52
297,72	198,28	247,08	377,06	331,70	352,24

PROGESTERONE

NC	*DC*	*Gli*	*Ao AE50*	*Ao AE100*	*Ao AE200*
58,58	*56,26*	51,34	54,71	50,29	53,88
49,90	52,17	56,81	45,59	54,71	55,76
52,11	55,76	*45,15*	71,73	*73,61*	58,80
56,10	51,67	52,61	58,91	59,53	56,14
54,18	*53,20*	53,59	57,75	54,84	56,15

DATA OF FETAL PARAMETERS

FETAL WEIGHT

NC	DC	Glib	*AoAE50*	*AoAE100*	*AoAE200*
2,57	2,04	2,64	2,95	2,34	2,58
2,86	2,14	*1,34**	3,03	2,62	3,74
3,33	2,70	3,43	2,27	2,29	2,60
2,41	1,27	3,89	2,74	2,41	2,51
3,32	2,25	2,65	4,08	4,97	2,59
3,58	*4,37**		3,46		4,59
4,45			3,48		4,55
3,44					

MASSES and CORRESPONDING MASSES (Adequate, Small or Large) for GESTATIONAL AGE (AGA, SGA or LGA)

	pup1	pup2	pup3	pup4	pup5	pup6	pup7	pup8	pup9	pup10	pup11	Pops Mean Body Mass per Rat
	2,44	2,55	2,53	2,56	2,66	2,66	2,61					2,57
	2,66	2,48	2,76	2,92	3,12	2,85	2,48	3,15	3,19	2,98		2,86
	2,94	3,37	3,41	3,19	3,38	3,29	3,64	3,42	3,30			3,33
	2,43	2,28	2,46	2,47	2,45	2,42	2,37	2,41				2,41
	3,03	3,51	4,04	3,41	3,11	3,52	2,59					3,32
	3,56	4,02	3,54	3,85	3,95	3,30	3,39	3,02				3,58
	4,67	4,41	4,30	4,60	4,93	3,95	4,43	4,68	4,46	4,03		4,45
	3,77	3,14	3,48	3,52	3,35	3,50	3,52	3,32	3,21	3,65		3,44
**NC**												**Number of AGA, SGA and LGA**
	AGA	AGA	AGA	AGA	AGA	AGA	AGA					**7 AGA**
	AGA	AGA	AGA	AGA	AGA	AGA	AGA	AGA	AGA	AGA		**10 AGA**
	AGA	AGA	AGA	AGA	AGA	AGA	AGA	AGA	AGA			**9 AGA**
	AGA	AGA	AGA	AGA	AGA	AGA	AGA	AGA				**8 AGA**
	AGA	AGA	AGA	AGA	AGA	AGA	AGA					**7 AGA**
	AGA	AGA	AGA	AGA	AGA	AGA	AGA					**7 AGA**
	AGA	AGA	AGA	LGA	LGA	AGA	LGA	LGA	LGA	AGA		**5 AGA; 5 LGA**
	AGA	AGA	AGA	AGA	AGA	AGA	AGA	AGA	AGA	AGA		**10 AGA**

% of AGA, SGA and LGA

AGA	SGA	LGA
**92,65**	**7,35**	**0,00**

	pup1	pup2	pup3	pup4	pup5	pup6	pup7	pup8	pup9	pup10	pup11	Pops Mean Body Mass per Rat
	1,98	1,91	2,25	2,28	1,89	1,99	1,69	2,37	2,11	1,90		2,04
	2,21	2,13	1,93	2,10	1,96	2,16	2,05	2,51	2,18			2,14
	2,98	2,93	2,22	2,74	2,68	2,72	2,50	2,84				2,70
	1,53	1,29	1,19	1,21	0,95	1,46						1,27
	2,35	2,38	2,06	2,18	2,33	2,46	1,97	2,32	2,25			2,25
**DC**	5,15	4,71	4,61	4,36	5,41	3,68	2,66					4,37
												**Number of AGA, SGA and LGA**
	SGA	SGA	AGA	AGA	SGA	SGA	SGA	AGA	SGA	SGA		**7 SGA; 3 AGA**
	AGA	SGA	SGA	SGA	SGA	AGA	SGA	AGA	AGA			**5 SGA; 4 AGA**
	AGA	AGA	AGA	AGA	AGA	AGA	AGA	AGA				**8 AGA**
	SGA	SGA	SGA	SGA	SGA	SGA						**6 SGA**
	AGA	AGA	SGA	AGA	AGA	AGA	SGA	AGA	AGA			**2 SGA; 7 AGA**
	LGA	LGA	LGA	LGA	LGA	AGA	AGA					**5 LGA; 2 AGA**

% of AGA, SGA and LGA

AGA	SGA	LGA
**48,98**	**40,81**	**10,20**

	pup1	pup2	pup3	pup4	pup5	pup6	pup7	pup8	pup9	pup10	pup11	Pops Mean Body Mass per Rat
	2,15	3,02	2,01	2,21	2,23	2,22	2,31	4,97				2,64
	1,45	1,26	1,42	1,37	1,22	1,42	1,16	1,35	1,39			1,34
	2,85	3,07	2,76	2,85	2,86	4,54	5,06					3,43
	4,01	3,82	4,32	3,91	3,91	3,83	3,45					3,89
**Glib**	2,44	2,62	2,50	2,57	2,57	2,54	2,24	2,36	4,00			2,65
												**Number of AGA, SGA and LGA**
	SGA	AGA	SGA	AGA	AGA	AGA	AGA	LGA				**7 AGA; 1LGA**
	SGA	SGA	SGA	SGA	SGA	SGA	SGA	SGA	SGA			**9 SGA**
	AGA	AGA	AGA	AGA	AGA	LGA	LGA					**5 AGA; 2 LGA**
	AGA	AGA	AGA	AGA	AGA	AGA	AGA					**7 AGA**
	AGA	AGA	AGA	AGA	AGA	AGA	AGA	AGA	AGA			**9 AGA**

% of AGA, SGA and LGA

AGA	SGA	LGA
**70**	**7,50**	**22,50**

	pup1	pup2	pup3	pup4	pup5	pup6	pup7	pup8	pup9	pup10	pup11	Pops Mean Body Mass per Rat
	2,85	3,02	3,22	3,26	2,74	2,85	2,67	3,01				2,95
	2,97	3,09	2,99	2,96	3,17	2,88	3,18					3,03
	2,32	2,36	2,37	2,06	2,01	2,37	2,29	2,37	2,26			2,27
	2,95	3,00	2,52	2,64	2,58	2,63	2,89					2,74
	4,27	3,30	4,33	4,21	3,91	4,47						4,08
	3,94	3,45	3,34	3,46	3,44	3,28	3,28					3,46
	4,01	4,12	3,63	2,92	2,73							3,48
AOAE50												**Number of AGA, SGA and LGA**
	AGA	AGA	AGA	AGA	AGA	AGA	AGA	AGA				**8 AGA**
	AGA	AGA	AGA	AGA	AGA	AGA	AGA					**7 AGA**
	AGA	AGA	AGA	SGA	SGA	AGA	AGA	AGA	AGA			**7 AGA; 2 SGA**
	AGA	AGA	AGA	AGA	AGA	AGA	AGA					**7 AGA**
	AGA	AGA	AGA	AGA	AGA	LGA						**5 AGA; 1 LGA**
	AGA	AGA	AGA	AGA	AGA	AGA	AGA					**7 AGA**
	AGA	AGA	AGA	AGA	AGA							**5 AGA**

% of AGA, SGA and LGA

AGA	SGA	LGA
**98,00**	**2,00**	**0,00**

	pup1	pup2	pup3	pup4	pup5	pup6	pup7	pup8	pup9	pup10	pup11	Pops Mean Body Mass per Rat
	2,19	2,21	2,26	2,28	2,29	2,54	2,63					2,34
	2,63	2,20	2,84	2,79	2,43	2,51	2,59	2,83	2,73	2,66		2,62
	2,27	2,04	2,33	2,20	2,23	2,32	2,34	2,34	2,20	2,61		2,29
	2,37	2,15	2,48	2,42	2,32	2,45	2,46	2,58	2,46	2,46		2,41
AOAE100	5,51	5,50	5,32	5,51	4,84	5,37	5,28	4,88	2,49			4,97
												**Number of AGA, SGA and LGA**
	AGA	AGA	AGA	AGA	AGA	AGA	AGA					**7 AGA**
	AGA	AGA	AGA	AGA	AGA	AGA	AGA	AGA	AGA	AGA		**10 AGA**
	AGA	SGA	AGA	AGA	AGA	AGA	AGA	AGA	AGA	AGA		**9 AGA; 1 SGA**
	AGA	SGA	AGA	AGA	AGA	AGA	AGA	AGA	AGA	AGA		**9 AGA; 1 SGA**
	LGA	LGA	LGA	LGA	LGA	LGA	LGA	LGA	AGA			**1 AGA; 8 LGA**

% of AGA, SGA and LGA

AGA	SGA	LGA
**78,26**	**17,39**	**4,35**

	pup1	pup2	pup3	pup4	pup5	pup6	pup7	pup8	pup9	pup10	pup11	Pops Mean Body Mass per Rat
	2,83	2,88	3,14	2,76	1,73	2,15						2,58
	3,99	3,60	3.649	4,11	4,08	3,07	3,59					3,74
	2,75	2,54	2,65	2,10	2,41	2,66	2,81	2,95	2,52			2,60
	2,32	2,47	2,63	2,04	2,92	2,67						2,51
	2,56	2,53	2,69	2,64	2,50	2,57	2,56	2,65	2,52	2,64		2,59
	4,86	3,84	4,48	4,85	4,93	4,62	4,58	4,48	4,15	4,59	5,15	4,59
	4,53	4,58	4,76	4,39	4,35	4,52	4,72					4,55
AOAE200												**Number of AGA, SGA and LGA**
	AGA	AGA	AGA	AGA	SGA	AGA						**6 AGA**
	AGA	AGA	AGA	AGA	AGA	AGA	AGA					**7 AGA**
	AGA	AGA	AGA	SGA	AGA	AGA	AGA	AGA	AGA			**8 AGA; 1 SGA**
	AGA	AGA	AGA	SGA	AGA	AGA						**5 AGA; 1 SGA**
	LGA	LGA	LGA	LGA	LGA	LGA	LGA	LGA	LGA	LGA		**10 LGA**
	LGA	AGA	LGA	LGA	LGA	LGA	LGA	LGA	AGA	LGA	LGA	**2 AGA; 9 LGA**
	LGA	LGA	LGA	LGA	LGA	LGA	LGA					**7 LGA**

% of AGA, SGA and LGA

AGA	SGA	LGA
**50,00**	**3,57**	**46,43**

PLACENTAL MASS AND EFFICIENCY

PLACENTAL MASS (g)

NC	DC	Glib	*AoAE50*	*AoAE100*	*AoAE200*
2,75	4,41	3,64	4,31	2,21	1,72
2,91	3,78	3,43	3,73	4,91	4,63
4,22	2,48	2,99	3,89	3,80	5,13
4,35	4,90	3,32	3,45	3,39	3,08
2,55	4,30	3,67	3,27	2,75	3,73
2,98	2,89		3,01		4,79
4,12			3,97		4,20
4,11					

PLACENTAL EFFICIENCY

NC	DC	Gli	*AoAE50*	*AoAE100*	*AoAE200*
35,95	24,01	33,27	38,75	27,69	41,09
46,58	25,18	19,30	38,78	30,05	30,53
35,84	31,82	41,53	27,03	28,47	25,42
39,76	14,97	35,78	30,07	29,98	26,95
49,39	26,57	41,98	49,37	34,53	49,81
39,27	*51,49**		43,11		49,96
37,23			39,03		42,12
46,33					
