# Supplementary material for: Antioxidant and antidiabetic effects of Angylocalyx oligophyllus leaves aqueous extract in pregnant diabetic rats: Feto-maternal repercussions
Source: PLoS One. 2025 Nov 14;20(11):e0334166. doi: 10.1371/journal.pone.0334166 (PMC12617868; doi:10.1371/journal.pone.0334166)

PLOS ONE

Antioxidant and Anti-diabetic effects of Angylocalyx oligophyllus leaves aqueous extract in pregnant diabetic rats: Feto-maternal repercussions

--Manuscript Draft--

**Manuscript Number:**	PONE-D-25-21592
**Article Type:**	Research Article
**Full Title:**	Antioxidant and Anti-diabetic effects of Angylocalyx oligophyllus leaves aqueous extract in pregnant diabetic rats: Feto-maternal repercussions
**Short Title:**	Antioxidant/Anti-diabetic effects of Angylocalyx oligophyllus leaves aqueous extract in pregnant diabetic rats
**Corresponding Author:**	Marie Claire TCHAMADEU, Ph.D.<br>University of Douala: Universite de Douala<br>Douala, CAMEROON
**Keywords:**	Pregestational diabetes mellitus; pregnancy; Angylocalyx oligophyllus; Antioxidant; Anti-diabetic; Profertil; Fetoprotector.
**Abstract:**	Pregestational diabetes mellitus can lead to many adverse outcomes during pregnancy both in the mother and her embryo/fetus. Plant-based products are empirically used as an alternative strategy to reduce these disorders. To investigate the effects of the Angylocalyx oligophyllus leaves aqueous extract on diabetes-induced metabolic, reproductive and fetal developmental disorders in pregnant diabetic rats, the in vitro anti-α-amylase and antioxidant plant effects first were evaluated. Then, adult virgin female rats primarily made diabetic by streptozotocin (35 mg/kg) and normal ones were mated with adult male rats. The pregnant rats were distributed into normal and diabetic control groups receiving distilled water, and diabetic rats groups treated with the plant extract doses (50, 100 and 200 mg/kg) or Glibenclamide (standard; 10 mg/kg). Animals were orally treated from 1st to 19th day of gestation, daily weighted, blood glucose levels measured on 1st, 5th, 10th, 15th and 20th gestation days (gd). At the end of pregnancy, maternal diabetic and reproductive parameters, and fetal morphological parameters were analyzed. At the gd 20, there were significant hyperglycemia, altered glucose tolerance, increased total cholesterol, triglycerides, transaminases, reabsorptions sites, post-implantation losses and death fetuses, reduced 17-β-estradiol and numbers of pancreatic cells, corpora luteum, implantation sites and live fetuses in non-treated diabetic mothers, associated with reduced weight and placental and caudal malformations in offsprings. The A. oligophyllus leaves aqueous extract induced significant anti-α-amylase and antioxidant activities in vitro. In pregnant diabetic rats, the plant significantly ($p<0.5$-$p<0.001$) reduced the serum levels of glucose, total cholesterol, triglycerides, LDL-cholesterol, transaminases and post-implantation losses, increased the serum HDL-cholesterol and 17-β-estradiol, the number of pancreatic cells, implantation sites and borned-living fetuses, while reducing placental and caudal malformations, and normalizing fetal weights in offsprings. The A. oligophyllus supplementation during pregnancy would be beneficial in preventing reproductive complications related to diabetes mellitus.
**Order of Authors:**	Christian Takoukam Tenezogang
	Marie Claire Tchamadeu
	Sylvin Benjamin Ateba
	Wylliam Nana Yousseu
	Quelie Nzekuie Selakong
	Armel-Kevin Fotso Pechi
	Ahmadou Hassimatou
	Calvin Zangue Bogning
	Pascal Emmanuel Owona
	Modeste Wankeu-Nya
	Alain Bertrand Dongmo

Powered by Editorial Manager® and ProduXion Manager® from Aries Systems Corporation

	Dieudonné Lembe Massoma
**Additional Information:**	
**Question**	**Response**
**Financial Disclosure**<br><br>Enter a financial disclosure statement that describes the sources of funding for the work included in this submission. Review the submission guidelines for detailed requirements. View published research articles from *PLOS ONE* for specific examples.<br><br>This statement is required for submission and **will appear in the published article** if the submission is accepted. Please make sure it is accurate.<br><br>**Funded studies**<br>Enter a statement with the following details:<br>• Initials of the authors who received each award<br>• Grant numbers awarded to each author<br>• The full name of each funder<br>• URL of each funder website<br>• Did the sponsors or funders play any role in the study design, data collection and analysis, decision to publish, or preparation of the manuscript?<br><br>Did you receive funding for this work?	The author(s) received no specific funding for this work.
**Competing Interests**<br><br>Use the instructions below to enter a competing interest statement for this submission. On behalf of all authors, disclose any competing interests that could be perceived to bias this work—acknowledging all financial support and any other relevant financial or non-financial competing interests.<br><br>This statement is required for submission and **will appear in the published article** if the submission is accepted. Please make sure it is accurate and that any funding	The authors have declared that no competing interests exist

sources listed in your Funding Information later in the submission form are also declared in your Financial Disclosure statement.<br><br>View published research articles from *PLOS ONE* for specific examples.<br><br>**NO authors have competing interests**<br><br>Enter: *The authors have declared that no competing interests exist.*<br><br>**Authors with competing interests**<br><br>Enter competing interest details beginning with this statement:<br><br>*I have read the journal's policy and the authors of this manuscript have the following competing interests: [insert competing interests here]*<br><br>* typeset	
**Ethics Statement**<br><br>Enter an ethics statement for this submission. This statement is required if the study involved:<br><br>• Human participants<br>• Human specimens or tissue<br>• Vertebrate animals or cephalopods<br>• Vertebrate embryos or tissues<br>• Field research<br><br>Write "N/A" if the submission does not require an ethics statement.<br><br>General guidance is provided below. Consult the submission guidelines for detailed instructions. **Make sure that all information entered here is included in the Methods section of the manuscript.**	The experimental procedure was conducted in accordance with All Guidelines for Care and Use of Laboratory Animals as described in the European Community Guidelines (EEC Directive 2010 / 63 / EU of September 22, 2010) and after the approval for Animal Experimentation N°2512 CEI-UDO/04/2021/T by the Institutional Ethics Committee of the University of Douala.<br>At the end of experiment (day 20 of pregnancy), pregnant rats were sacrificed by decapitation under anesthesia (Ketamine/Diazepam complex (70/30))

**Format for specific study types**<br><br>**Human Subject Research (involving human participants and/or tissue)**<br>• Give the name of the institutional review board or ethics committee that approved the study<br>• Include the approval number and/or a statement indicating approval of this research<br>• Indicate the form of consent obtained (written/oral) or the reason that consent was not obtained (e.g. the data were analyzed anonymously)<br><br>**Animal Research (involving vertebrate animals, embryos or tissues)**<br>• Provide the name of the Institutional Animal Care and Use Committee (IACUC) or other relevant ethics board that reviewed the study protocol, and indicate whether they approved this research or granted a formal waiver of ethical approval<br>• Include an approval number if one was obtained<br>• If the study involved *non-human primates*, add *additional details* about animal welfare and steps taken to ameliorate suffering<br>• If anesthesia, euthanasia, or any kind of animal sacrifice is part of the study, include briefly which substances and/or methods were applied<br><br>**Field Research**<br><br>Include the following details if this study involves the collection of plant, animal, or other materials from a natural setting:<br>• Field permit number<br>• Name of the institution or relevant body that granted permission	
**Data Availability**<br><br>Authors are required to make all data underlying the findings described fully available, without restriction, and from the time of publication. PLOS allows rare exceptions to address legal and ethical concerns. See the PLOS Data Policy and FAQ for detailed information.	Yes - all data are fully available without restriction

Powered by Editorial Manager® and ProduXion Manager® from Aries Systems Corporation

A Data Availability Statement describing where the data can be found is required at submission. Your answers to this question constitute the Data Availability Statement and **will be published in the article**, if accepted.<br><br>**Important:** Stating 'data available on request from the author' is not sufficient. If your data are only available upon request, select 'No' for the first question and explain your exceptional situation in the text box.<br><br>Do the authors confirm that all data underlying the findings described in their manuscript are fully available without restriction?	
**Describe where the data may be found in full sentences. If you are copying our sample text, replace any instances of XXX with the appropriate details.**<br><br>• If the data are **held or will be held in a public repository**, include URLs, accession numbers or DOIs. If this information will only be available after acceptance, indicate this by ticking the box below. For example: *All XXX files are available from the XXX database (accession number(s) XXX, XXX.)*.<br>• If the data are all contained **within the manuscript and/or Supporting Information files**, enter the following: *All relevant data are within the manuscript and its Supporting Information files.*<br>• If neither of these applies but you are able to provide **details of access elsewhere**, with or without limitations, please do so. For example:<br><br>*Data cannot be shared publicly because of [XXX]. Data are available from the XXX Institutional Data Access / Ethics Committee (contact via XXX) for researchers who meet the criteria for access to confidential data.*<br><br>*The data underlying the results presented in the study are available from (include the name of the third party*	All relevant data are within the manuscript and its Supporting Information files.

and contact information or URL).<br>• This text is appropriate if the data are owned by a third party and authors do not have permission to share the data.<br><br>* typeset	
Additional data availability information:	Tick here if the URLs/accession numbers/DOIs will be available only after acceptance of the manuscript for publication so that we can ensure their inclusion before publication.; Tick here if your circumstances are not covered by the questions above and you need the journal's help to make your data available.

Title, author list, and affiliation page

Click here to access/download;Manuscript;Title, author list, and affiliations page.docx

Antioxidant and Anti-diabetic effects of *Angylocalyx oligophyllus* leaves aqueous extract in pregnant diabetic rats: Feto-maternal repercussions

Christian Takoukam Tenezogang[1,¶], Marie Claire Tchamadeu[1,¶,*], Sylvin Benjamin Ateba[1,&], William Nana Yousseu[1,&], Quelie Nzekuie Selakong[1,ð], Armel-Kevin Fotso Pechi[1,ð], Ahmadou Hassimatou[1,ð], Calvin Zangue Bogning[1,&], Pascal Emmanuel Owona[2,&], Modeste Wankeu-Nya[1,&], Alain Bertrand Dongmo[1,&], Dieudonné Lembe Massoma[1,¶]

[1]Department of Biology and Physiology of Animal organisms, Faculty of Science, University of Douala, Douala, Cameroon

[2]Department of Animal Biology and Physiology, Faculty of Science, University of Yaoundé 1, Yaounde, Cameroon

[*]Corresponding author:

E-mail: *marieclaire_tchamadeu@yahoo.fr* (MCT)

[¶] These authors contributed equally to this work and are Joint Senior Authors

[&] These authors also contributed equally to this work

[ð] These authors also contributed equally to this work

Manuscript Click here to access/download;Manuscript;Manuscript body.docx

Abstract

Pregestational diabetes mellitus can lead to many adverse outcomes during pregnancy both in the mother and her embryo/fetus. Plant-based products are empirically used as an alternative strategy to reduce these disorders. To investigate the effects of the *Angylocalyx oligophyllus* leaves aqueous extract on diabetes-induced metabolic, reproductive and fetal developmental disorders in pregnant diabetic rats, the in vitro anti-α-amylase and antioxidant plant effects first were evaluated. Then, adult virgin female rats primarily made diabetic by streptozotocin (35 mg/kg) and normal ones were mated with adult male rats. The pregnant rats were distributed into normal and diabetic control groups receiving distilled water, and diabetic rats groups treated with the plant extract doses (50, 100 and 200 mg/kg) or Glibenclamide (standard; 10 mg/kg). Animals were orally treated from 1st to 19th day of gestation, daily weighted, blood glucose levels measured on 1st, 5th, 10th, 15th and 20th gestation days (gd). At the end of pregnancy, maternal diabetic and reproductive parameters, and fetal morphological parameters were analyzed. At the gd 20, there were significant hyperglycemia, altered glucose tolerance, increased total cholesterol, triglycerides, transaminases, reabsorptions sites, post-implantation losses and death fetuses, reduced 17-β-estradiol and numbers of pancreatic cells, corpora luteum, implantation sites and live fetuses in non-treated diabetic mothers, associated with reduced weight and placental and caudal malformations in offsprings. The *A. oligophyllus* leaves aqueous extract induced significant anti-α-amylase and antioxidant activities in vitro. In pregnant diabetic rats, the plant significantly ($p<0.5$-$p<0.001$) reduced the serum levels of glucose, total cholesterol, triglycerides, LDL-cholesterol, transaminases and post-implantation losses, increased the serum HDL-cholesterol and 17-β-estradiol, the number of pancreatic cells, implantation sites and borned-living fetuses, while reducing placental and caudal malformations, and normalizing fetal weights in offsprings. The *A.*

oligophyllus supplementation during pregnancy would be beneficial in preventing reproductive complications related to diabetes mellitus.

Key words: Pregestational diabetes mellitus; Pregnancy; *Angylocalyx oligophyllus*; Antioxidant; Anti-diabetic; Profertil; Fetoprotector.

Introduction

Diabetes mellitus is a growing public health problem, responsible for 10.4% of global mortality [1], affecting many organs and systems including the reproductive system. Men and women with diabetes mellitus have a significant decrease in fertility, particularly in cases of early onset or in childbearing age [2]. In women, whether before or during gestation (pregestational or gestational), diabetes mellitus can have a negative impact on pregnancy with increased risks for the mother and child [3,4]. In 2021, approximately 21.1 million live births (16.7%) suffered from some form of hyperglycemia during pregnancy, with 80.3% related to gestational diabetes, 10.6% related to diabetes (type 1 and type 2) pre-existing to pregnancy, and significant disparities between countries [5,6]. The prevalence of pregnancy complicated by diabetes varies from 1 to 14% across countries, with the most affected being low- and middle-income countries where access to maternal care is limited [5,6,7]. The periconceptional period and early gestation are critical and vulnerable windows of pregnancy to environmental influences that can alter the course of embryonic/fetal development and impose lasting health effects throughout life [8]. On the one hand, hyperglycemia is detrimental to conception and the implantation phase [9]. The maternal diabetic environment (hyperglycemia, insulin deficiency and/or failure) is associated with increased rates of embryonic resorption and malformation, placental dysfunction, increased risk of fetal, neonatal and obstetric complications, and a 4- to 10-fold increased risk in infants of developing diabetes in adulthood [10]. Furthermore, in diabetic women, pregnancy

accelerates the microvascular complications of diabetes leading to adverse fetal-maternal outcomes [11]. These adverse outcomes include fetal and neonatal death, congenital anomalies, preterm delivery, macrosomia or microsomia, associated with a series of obstetric complications (birth trauma, stillbirth, respiratory distress syndrome and neonatal hypoglycemia or hyperglycemia) [12]. Maternal complications include a high risk of pre-eclampsia, cesarean section, worsening of pre-existing diabetic complications and, in exceptional cases, maternal death [10, 13]. The pathophysiological mechanisms involved in the occurrence and maintenance of these disorders are multiple and complex.

In women with type 1 diabetes, insulin deficiency can contribute to a state of malnutrition, reduce the leptin signal and, through the KNDy (kisspeptin, neurokinin B, dynorphin) neural network, lead to a gonadotropic deficiency (impairment of ovarian functions) which can result in a reduction in the quantity and quality of gametes [14]. Fluctuations in insulin sensitivity during pregnancy, mostly due to changing hormone levels, complicate insulin replacement in gravid patients with Type 1 diabetes [15]. In addition, hyperglycemia can lead to an acceleration of ovarian apoptosis, leading to a decrease in ovarian reserve, or even premature ovarian failure. The possible association with autoimmune oophoritis can, of course, further aggravate this development [14]. Hyperglycemia will also generate inflammatory and pro-oxidative factors aggravating the alterations. Pregestational diabetes mellitus induces periconceptional changes that are accompanied by increased expression of the pro-inflammatory cytokines Trail, an increase in pro-inflammatory mediators in the uterus (Il-1α, TNF, etc.) and local dynamic changes in T lymphocytes. These T-cell changes impair the adaptive immune response to pregnancy with a 60 % reduction in anti-inflammatory regulatory T-cells in lymph nodes draining the uterus [16]. Maternal hyperglycaemia is also associated with oxidative stress, a hypoxic state of the embryo and changes in the regulation of apoptotic pathways, which modify all the crucial signaling events during prenatal

development, the expression of genes linked to morphogenesis and the integrity of genetic material [17]. Futhermore, numerous metabolic pathways have been identified associating diabetes mellitus and fetopathy. The diabetic fetopathy is thought to be the possible cause of perinatal losses in patients with pre-existing diabetes. The alterations in the transport of triglycerides, glucose and amino acids, associated with insulin resistance linked to pregnancy and oxidative stress, have been reported to be manifestations of the development of maternal microvascular complications and fetal disorders [18]. The frustration associated with the precipitation of micro-vascular complications and fetal malformations in pre-gestational diabetes calls for a preventive and alternative solution. In low- and middle-income countries where feto/maternal conditions are more common due to limited access to maternal care, medicinal plants are widely used as an alternative [19]. One of them, *Angylocalyx oligophyllus,* is a common plant of Cameroon, Nigeria, Benin, Gabon and the Belgian Congo used as alternative by women with hyperglycemia in pregnancy. This plant is vernacularly called elongo or etongo by the Baka and Iboa peoples, biem, sumba, nvuti etc. in Congo [20, 21]. The Song-Bong people of southern Cameroon use *A. oligophyllus* in traditional medicine to treat eye infections and diabetes. In addition, *in vitro* pharmacological studies of the plant have shown antioxidant, anti-urease and anti-α-glucosidase potential [22]. In light of its ethno-pharmacological uses and phytochemical composition, *A. oligophyllus* is hypothesized to possess preventive or therapeutic activities against maternal and fetal complications arising from diabetes during gestation. Therefore, given that streptozotocin (STZ) is the most commonly used diabetogenic chemical for creating rat models of type 1 and type 2 diabetes and Wistar rats are highly sensitive to it [23], this study aimed to evaluate the preventive properties of an aqueous extract from *A. oligophyllus* leaves on reproductive complications in pregnant, STZ-induced diabetic rats, as well as its antidiabetic and antioxidant effects.

Materials and methods

The study utilised the in-vitro and in-vivo experimental methods conducted over 2-months, with the in-vivo study conducted over 42 days.

Ethical approval

The experimental procedure was conducted in accordance with All Guidelines for Care and Use of Laboratory Animals as described in the European Community Guidelines (EEC Directive 2010 / 63 / EU of September 22, 2010) and after the approval for Animal Experimentation N°2512 CEI-UDO/04/2021/T by the Institutional Ethics Committee of the University of Douala.

Drugs and reagents

Iron-chlorid, sodium chloride, Kallium peroxodisulfat, potassium hydroxide were purchased from Acros organics (Germany); Natrium carbonat (Na_2CO_3), Tris-HCl and reagent from Roche (Germany); 2-deoxy-D-ribose, sodium hydroxide (NaOH) and sodium nitrite ($NaNO_2$) were obtained from Alfa Aesar (Germany). Phosphate buffered saline (PBS) and NED were purchased from VWR life science (Belgium).

Hydrogen peroxide HR rapid was from Tintometer group GmbH. Trichloroacetic acid and gallic acid were from Cayman chemical company. Alanine aminotransferase (ALT), Aspartate aminotransferase (AST), Total cholesterol, Triglycerides, HDL and total protein (TP) reagent kits were purchased at SGM Italia (Roma).

Trolox (6-hydroxy-2,5,7, 8-tetramethylchromane-2-carboxylic acid), sulfanilamide, Potassium ferricyanide [$K_3Fe(CN)_6$], potassium persulphate, 2,2- Diphenyl-1-picrylhydrazyl (DPPH), thiobarbituric acid, 2,2'-azino-bis-(3-ethylbenzothiazoline-6-sulphonic acid)

(ABTS), disodium hydrogen phosphate (Na_2HPO_4), phosphoric acid, ferric chloride and ascorbic acid were obtained from Sigma Aldrich (Germany).

Plant material

The leaves of *Angylocalyx oligophyllus* were harvested in the Center Region of Cameroon (Song-Bong, at 56 km from Eseka) in February 2021, after identification of a sample in the National Herbarium of Cameroon, in comparison with the voucher registered under the number N° 55817/HNC. The leaves were air-dried and then powdered using a grinder.

Extraction and standard preparation

Preparation and dosage determination of the *A. oligophyllus* leaves aqueous extract were done as previously describe [24]. Briefly, 366.6 g of leaves power were boiled in 2.5 L of distilled water for 20 min. After cooling, the mixture was filtered using a Whatman grade 3 qualitative filter paper. The leaf residue was again decocted following the same procedure. Both filtrates were mixed and evaporated in the oven at 40˚C. After evaporation, a mass of 11.813 g of extract was obtained representing a 3.22% extract yield.

The dosage selection of *A. olligophyllus* aqueous extract was based on the dose empirically administered by the traditional healer, and the therapeutic dose determined during the acute toxicity test [24,25].

Phytochemical analysis of *A. olligophyllus* leaves aqueous extract

Phytochemical analysis of *A. olligophyllus* leaves aqueous extract was carried out by qualitative methods, and polyphenol and flavonoids contents of the plant leaf tissue were investigated.

Qualitative phytochemical analysis

Qualitative phytochemical screening of the *A. oligophyllus* leaves aqueous extract was carried out following standard procedures as previously described to reveal the presence of saponins (Frotting test), alkaloids (Wagner test), polyphenols ($FeCl_3$ and $K_3Fe(CN)_6$ test), flavonoids and flavonols (Wilstater test), tannins and cathechic tannins ($FeCl_3$, test), triterpenoids (Liebermann-Burchard test), thiol proteins (Biuret test), unsatured steroids (Salkowski test) [26], carotenoids [27] and free quinones (Ether/NaOH test) [28]

Quantitative phytochemical analysis

Total polyphenols determination: The total polyphenolic content in aqueous extract of *A. oligophyllus* leaves was determined according to the Folin-Ciocalteu procedure [29]. About 0.2 mL of *A. oligophyllus* aqueous extract (2 mg/mL) was mixed with 1.2 mL of distilled water and 0.2 mL of Folin-Ciocalteu reagent (10%). After 3 minutes, 0.4 mL of sodium carbonate (Na_2CO_3, 7.5%) was added to the mixture which was immediately shaked and incubated for 20 minutes in a water bath at 40°C. Absorbance was measured in a spectrophotometer (UV-Genesis, Germany) at 760 nm and the results were expressed as gallic acid equivalents from a gallic acid standard curve (mgGAE/100g^{-1} Aqueous Extract; r^2=0,943). The analyses were performed in triplicate.

Total flavonoid content determination: The total flavonoid content in *A. oligophyllus* leaves aqueous extract was determined following the aluminum chloride colorimetric method of Zhishen et al. [30]. Briefly, a complexe solvent methanol–distilled water–acetic acid was previously prepared (140:50:10, v/v). Then, dried aqueous extract (2 mg) of *A. oligophyllus* leaves was homogenized with the solvent (1 mL) and filtered using Wattman N$^{o.}$3 filter paper. The aluminum chloride ($AlCl_3$) reagent solution was also prepared by dissolving 133 mg of

aluminum chloride crystals and 400 mg of sodium acetate crystals in 100 mL of solvent. To 0.2 mL of the extract filtrate was added 1 mL of aluminum chloride ($AlCl_3$) reagent solution and the whole was mixed and incubated for one hour at room temperature. The absorbance was measured at 430 nm using UV-Vis spectrophotometer. The analysis was performed in triplicate. The total flavonoid content was estimated from a quercetin standard curve and the results are expressed as mg quercetin equivalents (mg QE/$100g^{-1}$ Aqueous Extract; r^2= 0.9891).

Assessment of the *in vitro* anti-α-amylase and antioxidant activities of *A. oligophyllus* leaves aqueous extract

α-amylase activity inhibition test

The α-amylase inhibition assay was determined according to the modified method of Apostolidis and Lee [31]. Different concentrations (50, 25, 12.5 and 6.125 mg/mL) of *A. oligophyllus* leaf aqueous extract were prepared in 500 µL of phosphate buffer solution (0.02 M; pH 6.9; NaCl 0.006 M) and then, 100 µL of α-amylase solution were added to each tube. After incubation at 25°C for 10 min, 500 µL of starch solution (1% w/v) was added to each tube and the mixture incubated once again (25°C for 10 min). The reaction was stopped by adding 1 mL of 3,5-dinitrosalicylic acid (DNSA) reagent solution. The test tubes were incubated in a water bath at 95°C for 5 min and then cooled to room temperature. In addition, 10 mL of distilled water was added to the reaction mixture and the absorbance was measured at 540 nm against the blank. Acarbose, the standard drug, was used at the same concentrations as the extract. The α-amylase inhibitory activity was expressed as a percentage of inhibition and calculated according to the following formula:

$$\textbf{Inhibition of } \boldsymbol{\alpha} - \textbf{amylase } (\%) = \frac{\textbf{Abs standard} - \textbf{Abs sample}}{\textbf{Abs standard}} \times \mathbf{100}$$

DPPH radical scavenging assay

DPPH free radical scavenging activity of *A. oligophyllus* leaf aqueous extract was assessed using the method described by Mensor et al. [32]. The *A. oligophyllus* extract was dissolved in methanol (1000 µg/mL). Then, 50 µL of this solution was mixed with 150 µL of 0.02% methanolic solution of DPPH to give a final extract concentration ranging from 1000 to 1 µg/mL (1000, 700, 300, 100, 10, 3 and 1µg/mL). After 30 min incubation in the dark at room temperature, the optical density was measured at 517 nm using a UV/Genesis light spectrophotometer (UV-Genesis, Germany). Ascorbic acid (Vitamin C) was used as a positive control. Each assay was performed in triplicate and results were expressed as percentage of inhibition and calculated according to the following formula:

$$\textbf{Inhibition of DPPH}\ (\%) = \frac{\textbf{Abs standard} - \textbf{Abs sample}}{\textbf{Abs standard}} \times \mathbf{100}$$

ABTS scavenging assay

ABTS $^{+}$ free radical scavenging activity of *A. oligophyllus* leaf aqueous extract was assessed using the method described by Re *et al*. [33]. The *A. oligophyllus* extract was dissolved twice in methanol (1000 µg/mL). 25 µL of the diluted extract was mixed with 65 µL of methanolic solution of 2,2'-azino-bis (3-ethylbenzothiazoline-6-sulphonic acid) (ABTS), to obtain a final concentration of the extract ranging from 1000 to 1 µg/mL (1000, 700, 300, 100, 10, 3 and 1µg/mL). After 30 min incubation in the dark at room temperature, the absorbances were measured at 734 nm using a UV/Genesis light spectrophotometer (UV-Genesis, Germany). Ascorbic acid (Vitamin C) was used as a control. Each assay was performed in triplicate and results were expressed as a percentage of inhibition and calculated according to the following formula:

$$\textbf{Inhibition of ABTS } (\%) = \frac{\textbf{Abs standard} - \textbf{Abs sample}}{\textbf{Abs standard}} \times \mathbf{100}$$

Hydroxyl scavenging assay

Hydroxyl radical scavenging generated by the Fenton reaction was measured using the modified protocol of Rao and Kunchandy [34]. To 500µL of different concentrations (1-1000 µg/mL) of the *A. oligophyllus* extract or Trolox (standard) were added 100 µL of 2-deoxy-D-ribose (28 mM), 100 µL EDTA (1.04 mM), 100 µL $FeCl_3$ (0.2 mM) (v/v, 1:1), 100 µL H_2O_2 (1 mM) and 100 µL ascorbic acid (1 mM). The mixture (M1) was incubated at 37°C for 1 hour. Thereafter 500 µL of thiobarbituric acid (TBA) (1 %) and 500 µL of trichloroacetic acid (TCA) (2.8%) were added to the mixture M1and incubated at 100˚C for 30 min. After cooling and reaching the room temperature, the absorbance of the solution was measured at 532 nm against the blank. Results were expressed as percentage degradation of 2-deoxyribose:

$$\textbf{Inhibition of 2} - \textbf{deoxyribose degradation}(\%) = \frac{\textbf{Astandard} - \textbf{Asample}}{\textbf{Astandard}} \times \mathbf{100}$$

FRAP assay

The reducing capacity of the extract was performed according to previously described method with modifications [35]. 0.5 mL of extract/ascorbic acid (1-300 µg/mL) were pipetted and introduced in the test tubes in triplicate long with 1.25 mL of phosphate buffer (200 mM, PH= 6.6) and 1.25 mL of potassium ferricyanide (1%). The mixture was incubated at 50°C for 20 min before adding 1.25 mL of trichloroacetic acid (10%). Following the centrifugation (3000 rpm for 10 min), 1.25 mL of the supernatant was taken away and mixed with 1.25 mL of distilled water and 0.25 mL of $FeCl_3$ (0.1%). This mixture was incubated for 10 min at 37°C and the absorbance measured at 700 nm against the blank.

Assessment of the effects of *A. oligophyllus* leaves aqueous extract in pregnant diabetic rats

Animal material

The in-vivo study was conducted over 42 days, using 89 Wistar rats (77 female and 12 male) aged 10 to 12 weeks and weighing 160 ± 20 g. Vaginal smears of adult normal female rats were examined every morning using a pipet tube and 0.9%, and those exhibiting at least 3 successive and regular estrous cycles were selected for the study. All animals were raised in the animal facility of the Laboratory of Animal Biology and Physiology, Faculty of Science, University of Douala, and housed at room temperature in plastic cages lined with shavings. Rats were housed three or one animals per cage respectively for non-pregnant or pregnant rats. Each housing cage contained information related to the experimental period, as well as the type, dose, and duration of the treatment. Animals were maintained under a natural light/dark cycle and fed a diet consisting of 54% maize meal, 4% wheat meal, 20% fish meal, 10% maize groundnut meal, 3% bone meal, 7% palm kernel oil, 2% salt, and 0.02% vitamin complex. They received tap water as drink *at libitum*.

Induction of type 1 diabetes in female rats

The streptozotocin (STZ) solution concentrated at 40 mg/mL was prepared by dissolving 250 mg of STZ powder in 6.25 mL of 0.9% NaCl solution for diabetes induction (STZ from Sigma Chemical Compagny, St Louis, Meule, USA). Type 1 diabetes was induced in 67 normal female rats exhibiting a regular estrous cycle by intraperitoneal injection of a streptozotocin solution at the dose of 35 mg/kg body weight. Ten another normal female rats with regular estrous cycle used as normal control received the equal volume of the vehicle (0.9% NaCl) [36]. Using an Accu Chek Active glucometer (Sandhofer Strasse, Mannheim,

Germany), diabetes was confirmed 72 hours after STZ injection by a fasting blood glucose level > 220 mg/dL [37]. If necessary, rats that did not exhibit hyperglycaemia received one or two additional 15 mg/kg injections of STZ solution, administered after diabetes screening on the third and fifth days following the first STZ injection [38].

Experimental groups and procedure

Vaginal smears of diabetic and non-diabetic female rats were again examined every morning, and those at the proestrus phase of the estrous cycle were mated overnight with adult male breeder rats (One male with two female rats). The next morning, the observation of spermatozoa in the vaginal smear of a diabetic or non-diabetic female rat was the evidence of the copulation and a probable fertilization, and thus marked the first day (D1) of gestation. The mating period lasted at most 15 days, approximately 3 estrous cycles, allowing to increase the chance of obtaining a large number of pregnant females. During this period, the vaginal smears were examined every morning until fertilization. The non-fertilized female rats, considered as infertile, were excluded from the study [39]. To ensure at least five effectively pregnant rats in each group for reliable statistical analysis by the experiment's end, fertilized rats presumed as pregnant were randomly assigned into six experimental groups based on their initial fasting blood glucose levels as follows :

- Group 1 or normal control (n = 10): pregnant non diabetic rats receiving distilled water (10 mL/kg).
- Group 2 or diabetic control (n = 20): pregnant diabetic rats receiving distilled water (10mL/kg);
- Group 3 or positive control (n = 12): pregnant diabetic rats treated with glibenclamide (10 mg/kg);

- Groups 4, 5 and 6 (n = 10 for each group): pregnant diabetic rats receiving the *A. oligophyllus* leaves aqueous extract at the doses of 50, 100 and 200 mg/kg, respectively.

Animals were thus treated by gavage and monitored every day (in the morning) for 20 days. Those that did not exhibit developed pregnancy 12 days after their probable fertilisation were removed. Those displaying a sudden and drastic loss of body mass, greatly reduced mobility, extreme fatigue, or abnormal behaviors during the course of the experiment should be euthanized under ketamine and diazepam (80/20 mg/kg) within 24 hours, in case of any recovery. Only effective pregnant normal and diabetic rats were monitored for pregnancy course.

NB : All removed female rats (those that did not be fertilized over 15-days of mating, and those that did not exhibit developed pregnancy 12 days after their fertilisation) were used in another study.

Pregnancy course monitoring

Maternal body weight and fasting blood glucose levels were measured every 5 days up to the end of pregnancy. Oral glucose tolerance (OGTT) and insulin sensitivity tests were performed on days 17 and 18 of gestation, respectively, as described by Kiss et al. [40]. At the day 20 of pregnancy, pregnant rats were sacrificed under anesthesia (Ketamine/Diazepam complex (70/30)). The whole blood was collected in dry tubes and centrifuged at 3000 rpm for 15 min. The collected serum was separated in two aliquots and stored at -20°C for the measurement of serum 17-β-estradiol and progesterone levels, as well as other serum biochemical parameters. Uterine, liver, kidney, spleen, heart, pancreas, aorta, abdominal fat, pre-gonadic fat, brain, adrenal gland and ovaries were collected and weighed. The uterine horns were dissected to determine the number of live and/or stillborn fetuses and examine their morphology. The number of implantation and resorption sites was determined. Placentas were also removed,

weighed and their diameter measured. Placental efficiency was calculated according to Wilson and Ford formula [41]. The maternal pancreas and placenta were removed, weighed and fixed in 10% formalin buffer for histological sections.

Oral Glucose Tolerance Test in pregnant normal and diabetic rats

An Oral Glucose Tolerance Test (OGTT) was performed, as described by Kiss et al. [40]. Briefly, on day 17 of gestation, pregnant normal and diabetic rats were weighted and fasted for 06 h. After fasting, animals were administered a single oral dose of D-glucose solution (3 mg/kg) (groups 2-6 or DC, Gli, AoAE50, AoAE100 and AoAE200 groups) or an equal volume of solvent (group 1or NC) by gavage. Blood glucose levels were measured before the D-glucose administration (0 minute), and at 30, 60, and 120 minutes after. At the end of the test, animals received their daily oral treatments by gavage as up described.

Insulin Sensitivity Test in pregnant normal and diabetic rats

An insulin sensitivity test was performed on day 18 of gestation as described by Kiss et al. [40]. Briefly, non-fasted pregnant normal and diabetic rats were weighed and received a single dose of insulin solution (2 IU/kg) in *I.P.* Blood glucose levels were measured before the insulin administration (0 minute), and at 30 and 60 minutes after. At the end of the test, a glucose solution (0.5g/kg) was administered to animals in drinking water for 24 hours, in order to counteract possible cases of severe hypoglycemia following the insulin injection [42], allowing sacrificing all animals at the end of experiment without death. Then, daily treatments were administrated oraly by gavage in each group as up described

Maternal blood glucose level measurement

Blood glucose determination (at 0, 30, 60 and 120 minutes for OGTT ; at 0, 30 and 60 minutes for Insulin sensitivity test ; and at 0, 5, 10, 15 and 20 days for sub-acute experiment) was carried out using ACCU-CHEK® active glucometer (Roche Diagnostics, Germany) as previously described [42,44].

Other maternal serum biochemical parameters analysis

After collected in dry tubes, maternal whole blood was centrifuged at 3000 rpm for 15 min and the obtained serum was aliquoted in two tubes and stored at –20°C for the measurement of serum estradiol and progesterone levels, as well as aspartate aminotransferase (AST) and alanine aminotransferase (ALT) activities. Serum concentrations of total protein, total cholesterol (TC), triglycerides (TG), and high-density lipoprotein cholesterol (HDL-C) were also measured, while low-density lipoprotein cholesterol (LDL-C) levels were calculated using Friedewald's formula: LDL-C = (TC) - (HDL-C) - (TG/5) [45].

Pregnancy outcomes and fetal development

The romoved gravid uteri were dissected for determining the number of live and dead fetuses, reabsorption (embryonic death), implantation sites and of luteal bodies. The number of undetectable implantation sites was determined as described by Costa-Silva et al. [46]. The rate of pre-implantation loss was calculated as [(number of corpora lutea – Number of implantations) x 100 / Number of implantations] [47]. Collected fetuses from the uterine horns were weighed for body weight classification according to the mean ± 1.7 x standard deviation (SD) of body weight obtained in the control group [48]. Placental efficiency defined in percent was calculated according to Wilson and Ford formula [41] as:

$$\textbf{Placental efficiency} = \frac{\textbf{Placental mass (g)}}{\textbf{Real mass of fetus (g)}} \times \textbf{100}$$

Statistical evaluation

GraphPad Prism 8.0.1 was used for all statistical evaluations, with differences considered statistically significant at $p<0.05$.

***In Vitro* Study:** For the *in vitro* study, each test was performed in triplicates, and results were expressed as Mean ± Standard Deviation (SD). The Kruskal-Wallis non-parametric test followed by Dunnett's post hoc test was used to analyze the *in vitro* antioxidant and antidiabetic capacities of the plant extract. The IC50 (concentration required to inhibit 50% radicals) values were computed by linear regression of each concentration tested, with the radical scavenging percentage as the response variable.

***In Vivo* Study:** Mean values among experimental groups in the *in vivo* study were compared as follows:

- Two-way ANOVA with Bonferroni’s post-test was used for comparing repeated measures data between groups, such as body weight and blood glucose.
- One-way ANOVA with Mann-Whitney was used for comparing experimental groups regarding serum biochemical parameters, as well as the number of implantations, live and dead fetuses, resorptions, and corpora lutea.
- Fisher’s exact test was used to analyze proportion data, including gestation percentage and fetal mass for gestational age.

Results

Qualitative and quantitative phytochemistry of *A. oligophyllus* aqueous extract

“Table 1” shows that thiols proteins, saponins, alkaloids, polyphenolic, flavonoids and flavonols, tannins and cathechic tannins, free quinones, triterpenoids, carotenoids and

unsaturated sterols compounds were identified in the *A. olligophyllus* leaves aqueous extract. Polyphenolic compounds being the group mostly explored in qualitative analysis, quantitative analysis of total polyphenols and the sub group flavonoids were also carried out and the results showed that total flavonoïds represent ≈ 68.74% of Total polyphenols “Table 1”

Table 1. Qualitative and Quantitative Phytochemical Analyses of *A. oligophyllus* leaves aqueous extract

Phytoconstituents	*A. oligophyllus* leaves aqueous extract		
	Qualitative analyses	Quantitative analyses	
		Total polyphenols (mGEA/g)	Total flavonoïds (mQE/g)
**Thiols proteins**	+		
**Saponins**	+		
**Alkaloids**	+		
**Polyphenols**	+	**145.0 ± 7.5**	**99.68 ± 11.34 (≈ 68.74% of Total polyphenols)**
- Flavonoids	+		
. Flavonols	+		
- Tanins	+		
. Cathechic tanins	+		
- Free quinones	+		
**Terpenes**	/		
- Triterpenoids	+		
- Carotenoids	+		
- Unsatured sterols	+		

+ = present; / = not verified; Values are expressed as mean ± SEM; n = 3

In vitro anti-α-amylase and antioxidant activities of *Angylocalyx oligophyllus* leaves aqueous extract

Anti-α-amylase activity of *A. oligophyllus* leaves aqueous extract

The plant's capacity to inhibit the α-amylase activity is shown in "Fig 1". The *A. oligophyllus* leaves extract displayed a concentration-dependent inhibitory effect with an inhibitory potency (IC_{50} = 27.51 mg/mL) closed to that of acarbose (IC_{50} = 22.94 mg/mL). The plant extract concentration of 50 mg/mL highly and significantly inhibited ($p<0.01$) the α-amylase activity (92.07 ± 2.66 %) compared to acarbose (82.27 ± 3.28%).

Fig 1. Effect of different concentrations of *A. oligophyllus* leaves aqueous extract on α-amylase activity.

Each bar represents mean ± SD; n = 3 per group; $^{\alpha\alpha}p<0.01$= significant difference to acarbose; $^{\beta}p<0.05$, $^{\beta\beta}p<0.01$, $^{\beta\beta\beta}p<0.001$= significant difference to plant extract.

Free radical scavenging and antioxidant activities of *A. oligophyllus* aqueous extract

The "Fig 2A" shows the DPPH (2, 2-diphenyl-1-picrylhydrasyl) radical scavenging activity. The aqueous extract of *A. oligophyllus* showed a good capacity to trap the DPPH free radical with an inhibitory concentration 50 (IC50) value of 61.60 µg/mL, while that of ascorbic acid (vitamin C) used as reference was 4.505 µg/mL. The effective concentration 50 (EC_{50}) and relative free radical scavenging capacity (RSP) of *A. oligophyllus* extract were 0.97 and 1.02 µg/mL, respectively, compared to those of vitamin C (0.07 and 13.98 µg/mL).

In the ABTS [2, 2'-azino-bis-(3-ethylbenzathiazoline-6-sulfonic) acid] assay, *A. oligophyllus* leaves aqueous extract, like Trolox, showed a concentration-dependent inhibition on ABTS cations "Fig 2B". The extract strongly inhibited $ABTS^{+}$ radicals with an IC_{50} of 7.097 µg/mL, close to that of Trolox (4.561 µg/mL).

In the case of FRAP assay "Fig 2C", the *A. oligophyllus* extract showed good ability to chelate the metal iron with IC_{50} of 36.26 µg/mL, approaching to that of vitamin C (IC_{50} = 4.61

μg/mL). However, the chelating power of the plant extract is approximately 8 folds as tower than that of vitamin C (“Fig 2C”).

The “Fig 2D” shows the scavenging capacity for hydroxyl radicals. This activity was concentration-dependent for both *A. oligophyllus* aqueous extract and Trolox. The extract IC_{50} value of 25.51 μg/mL was 3.34 times lower than that of the standard antiradical Trolox (85.29 μg/mL). Moreover, the extract showed greater concentration-dependent anti-peroxide activity than Trolox.

Fig 2. *A. oligophyllus* extract effects on (A) DPPH; (B) $ABTS^+$; (C) FRAP and (D) Hydrogen peroxide.

Each point represents mean ± SD; n = 3 per group.

Effects of *A. oligophyllus* leaves aqueous extract in diabetic pregnant rats

Effects of the plant extract on maternal body and relative organs masses

The body masses of pregnant normal and diabetic rats significantly increased ($p<0.05$ - $p<0.001$) during the gestation “Fig 3”. The pregnant diabetic rats showed a significant ($p<0.001$) reduced body mass gain from the 10th day compared to pregnant normal rats. Compared to diabetic control, the *A. oligophyllus* aqueous extract (*Ao*AE) at doses of 100 and 200 mg/kg significantly increased the body mass gain of pregnant diabetic rats from 10th to 20th days ($p<0.05$-$p<0.01$) of gestation, with the maximum increase of 12.18% ($p<0.05$) observed on day 20 at 200 mg/kg dose. This dose extract (200 mg/kg) also significantly increased the body mass gain of pregnant diabetic rats (7.13%; $p<0.05$) compared to glibenclamide on day 15 of gestation “Fig 3”.

"Table 2" shows in pregnant diabetic control rats significant liver and kidney relative weights increases and abdominal and peri-gonadic fat decreases (p<0.05-p<0.01) as compared to normal control. The administration of the plant extract to pregnant diabetic rats did not significantly decrease the liver relative weight increase, but, significantly decreased (p<0.05-p<0.01) the kidney relative weight at the dose extract of 100 mg/kg, as compared to pregnant diabetic control rats and to glibenclamide-treated ones. In terms of adipose tissue decrease, the *A. oligophyllus* extract at all doses increased the abdominal and peri-gonadic fat in pregnant diabetic rats, with the maximum and significant effects at the dose of 50 mg/kg (by 3.61 and 4.36 folds respectively; p<0.05), compared to pregnant diabetic control. Glibenclamide did not reduce liver and kidney relative masses increase, nor enhance fat masses compared to pregnant diabetic control rats. Moreover, all other organs relative masses did not significantly change between pregnant diabetic control and pregnant normal control rats, despite some increases in pancreas and spleen relative weights observed in glibenclamide- (p<0.05) and the 200 mg/kg extract dose- (p<0.05-p<0.001) treated pregnant diabetic groups as compared to pregnant normal control.

Fig 3. Body mass changes in pregnant normal and untreated and treated diabetic rats

Values are expressed as mean ± ESM (n = 5-9 per group); * p<0.05, ** p<0.01, ***p<0.001, = significant difference compared to NC; $^{\alpha}$p<0.05, $^{\alpha\alpha}$p<0.01= significant difference compared to DC; $^{\beta}$p<0.05= significant difference from Glib; NC= normal control; DC = diabetic control; Gli = Pregnant diabetic rats treated with glibenclamide; AoAE 50, AoAE 100 and AoAE 200 = Pregnant diabetic rats treated with *Angylocalyx oligophyllus* aqueous extract at the doses of 50, 100 and 200 mg/kg, respectively.

Table 2. Organs relative masses in pregnant normal and untreated and treated diabetic rats

Organs relative masses (g/g)	Treatments					
	NC (n=9)	DC (n=6)	Gli (n=5)	AoAE50 (n=8)	AoAE100 (n=5)	AoAE200 (n=7)
**Liver**	**2.88 ± 0.18**	**3.65 ± 0.20** **	**3.99 ± 0.22** *	**3.37 ± 0.11** $^{\beta}$	**3.26 ± 0.32**	**3.85 ± 0.27** *
**Kidney**	**0.46 ± 0.2**	**0.64 ± 0.04** **	**0.66 ± 0.06** **	**0.53 ± 0.03**	**0.48 ± 0.04** $^{\alpha\beta\beta}$	**0.68 ± 0.05** **
**Pancreas**	0.13 ±0.01	0.15 ± 0.02	0.18 ± 0.01 **	0.15 ± 0.01	0.15 ± 0.02	0.20 ± 0.03 ***
**Heart**	0.26 ± 0.01	0.28 ± 0.01	0.27 ± 0.01	0.28 ± 0.01	0.25 ± 0.02	0.31 ± 0.02 *
**Aorta**	0.035 ± 0.002	0.041 ± 0.012	0.042 ± 0.007	0.037 ± 0.007	0.033 ± 0.007	0.033 ± 0.007
**Spleen**	0.16 ± 0.02	0.28 ± 0.06	0.48 ± 0.15 *	0.23 ± 0.03	0.23 ± 0.04 *	0.32 ± 0.06**
**Abdominal fat**	**1.18 ± 0.21**	**0.23 ± 0.08** **	**0.37 ± 0.19** *	**0.83 ± 0.26** $^{\alpha}$	**0.95 ± 0.25**	**0.52 ± 0.16** *
**Peri-ovarian fat**	**1.28 ± 0.11**	**0.28 ± 0.12** **	**0.53 ± 0.21** *	**1.22 ± 0.18** $^{\alpha}$	**0.60 ± 0.09** **	**1.10 ± 0.27**$^{\alpha}$
**Brain**	0.71 ± 0.05	0.80 ± 0.02	0.85 ± 0.04	0.70 ± 0.07	0.72 ± 0.06	0.82 ± 0.02
**Adrenal gland**	0.031 ± 0.004	0.035 ± 0.003	0.042 ± 0.003	0.028 ± 0.002	0.033 ± 0.004	0.036 ± 0.004
**Ovaries**	0.036 ± 0.002	0.041 ± 0.012	0.042 ± 0.003	0.037 ± 0.007	0.033 ± 0.007	0.033 ± 0.007

Values are expressed as mean ± SEM; n = 5-9; $^{*}p<0.05$, $^{**}p<0.01$, $^{***}p<0.001$= significant difference compared to NC; $^{\alpha}p<0.05$= significant difference compared to DC; $^{\beta}p<0.05$, $^{\beta\beta}p<0.01$= significant difference compared to Gli; NC = normal control; DC = diabetic control; Gli = Pregnant diabetic rats treated with glibenclamide*;* AoAE 50, AoAE 100 and AoAE 200 = Pregnant diabetic rats treated with *Angylocalyx oligophyllus* aqueous extract at the doses of 50, 100 and 200 mg/kg, respectively.

Effect of the plant extract on maternal blood glucose

The fasting blood glucose remained significantly elevated ($p<0.01$-$p<0.0001$) in pregnant diabetic rats throughout pregnancy as compared to pregnant normal controls "Table 3". The administration of *A. oligophyllus* aqueous extract at all tested doses decreased this blood glucose from day 5 of gestation compared to pregnant diabetic control. This decrease was significant on days 15 and 20 of gestation at extract doses of 50 mg/kg (39.07% and 45.17% respectively; $p<0.01$) and 200 mg/kg (48.97%; $p<0.001$) compared to pregnant diabetic control. Furthermore, the glibenclamide administered to pregnant diabetic rats resulted in non-significant blood glucose decrease of 25.9% at 20th day of gestation compared to pregnant diabetic control.

Table 3. Blood glucose levels variation in pregnant normal and untreated and treated diabetic rats

Treatments	Times (Days)				
	**D1**	**D5**	**D10**	**D15**	**D20**
**NC (n=9)**	107.70 ± 7.68	99.20 ± 4.98	87.17 ± 4.25	91.83 ± 4.79	86.67 ± 4.73
**DC (n=6)**	350.40 ± 48.16**	391.00 ± 2.19****	360.20 ± 41.30***	372.20 ± 37.64****	397.20 ± 11.43****
**Gli (n=5)**	366.00 ± 44.03	359.20 ± 55.26	353.20 ± 28.22	354.40 ± 49.64	294.20 ± 26.16
**AoAE50 (n=8)**	331.00 ± 34.36	304.50 ± 93.75	317.00 ± 37.21	226.80 ± 49.45 $^{\alpha\alpha}$	217.80 ± 33.21 $^{\alpha\alpha}$
**AoAE100 (n=5)**	340.20 ± 42.75	250.40 ± 48.12	292.60 ± 20.30	298.80 ± 53.91	263.00 ± 52.25
**AoAE200 (n=7)**	317.20 ± 22.38	272.70 ± 30.20	282.20 ± 48.63	253.70 ± 38.95	202.70 ± 40.73$^{\alpha\alpha\alpha}$

Values are expressed as mean ± SEM; n = 5-9 per group; $^{*}p<0.05$, $^{**}p<0.01$, $^{***}p<0.001$, $^{****}p<0.001$= significant difference compared to NC; $^{\alpha}p<0.05$, $^{\alpha\alpha}p<0.01$, $^{\alpha\alpha\alpha}p<0.001$= significant difference compared to DC; NC = normal control; DC = diabetic control; Gli = Pregnant diabetic rats treated with glibenclamide; AoAE 50, AoAE 100 and AoAE 200 = Pregnant diabetic rats treated with *Angylocalyx oligophyllus* aqueous extract at the doses of 50, 100 and 200 mg/kg, respectively.

Effects of the plant extract on maternal oral glucose tolerance and insulin sensitivity

Thirty minutes after the administration of D-glucose, a significant increase of 50.91% ($p<0.01$) in postprandial glycaemia was observed in diabetic control rats compared to normal control “Fig 4A”. The administration of the plant extract at the dose of 200 mg/kg reduced this blood glucose increase by 21.85% compared to pregnant diabetic control and moreover by approximately 1.5 folds compared to glibenclamide-treated diabetic rats group. The glibenclamide reduced the pregnant diabetic rats’ postprandial glycaemia by 15.1% compared to pregnant diabetic control. However, the estimation of the area under the glycaemia curve (AUC) for each group, expressing mean blood glucose levels between 0 and 120 min, showed high and significant ($p<0.01$) blood glucose levels in diabetic groups than in the normal control group after carbohydrate loading. The plant extract doses of 100 and 200 mg/kg respectively inhibited the global postprandial glycaemia increase (AUC) by 9.5% and 16.1% ($p<0.05$) compared to the pregnant diabetic control “Fig 4A”.

“Fig 4B” shows insulin sensitivity in diabetic pregnant rats. The intra-peritoneal injection of insulin in pregnant diabetic rats resulted in a blood glucose levels decrease in all groups. However, 60 minutes after insulin injection, the blood glucose levels reduction was greater in pregnant diabetic control rats (83.6%; $p<0.001$) than in pregnant normal control rats (42.5%; $p<0.001$) as compared to the initial blood glucose values, which resulted in a reduced AUC values in diabetic control (50.73 mg/dL/min) compared with normal control (58.21 mg/dL/min). The plant extract at 100 mg/kg dose and glibenclamide respectively reduced the diabetic pregnant rats’ hypersensitivity (AUC) by 12.5% and 24.46% compared to pregnant diabetic control.

Fig 4. Oral glucose tolerance (A) and insulin sensitivity (B) in *A. oligophyllus* extract-treated pregnant diabetic rats

Values are expressed as mean ± SEM; n = 5-9 per group; ** p<0.01, ****p<0.0001= significant difference Compared to NC; $^{\alpha}$p<0.05, = significant difference compared to DC; AUC = Area Under the glycaemia Curve; NC = normal control; DC = diabetic control; Gli = Pregnant diabetic rats treated with glibenclamide; AoAE 50, AoAE 100 and AoAE 200 = Pregnant diabetic rats treated with *Angylocalyx oligophyllus* aqueous extract at the doses of 50, 100 and 200 mg/kg, respectively.

Effects of the plant extract on some of maternal biochemical parameters

Pregnant diabetic rats showed a significant increase (p<0.05-p<0.001) in serum transaminases (ALT and AST), triglycerides, total cholesterol, LDL-cholesterol and the AST/ALT ratio, and a significant decrease (p<0.05) in HDL-cholesterol compared to normal control rats "Table 4".

The *A. oligophyllus* aqueous extract at all tested doses reduced serum AST and ALT activities as well as AST/ALT ratio, with significant reductions observed at the 100 mg/kg dose for ALT (42.1%%; p<0.05), and at doses of 50, 100 and 200 mg/kg for AST (48.7%, 36% and 46.8% respectively; p<0.05-p<0.01) and AST/ALT ratio (55.9%, 65.1% and 56.7% respectively; p<0.01-p<0.001), all compared to the pregnant diabetic control "Table 4". In addition, the plant extract at doses of 50, 100 and 200 mg/kg also reduced (p<0.05- p<0.01) serum levels of triglycerides (inversely dose-dependantly by 36.1%, 33.1% and 24% respectively), total cholesterol (dose-dependantly by 36%, 41.9% and 47.7% respectively) and LDL-cholesterol (Inversely dose-dependantly by 42%, 33.7% and 12% respectively), while increasing HDL-cholesterol levels (dose-dependantly by 99.5%, 146.9% and 258.8% respectively; p<0.05-p<0.01), as compared to pregnant diabetic control. Interestingly, the plant extract doses of 100 and 200 mg/kg increased the HDL-cholesterol level by 5% and 52.54% respectively, compared with pregnant normal control rats.

“Table 4” shows that the glibenclamide also induced significant reductions in serum AST (38.1%; $p<0.05$) and ALT (51.6%; $p<0.5$) activities, AST/ALT ratio (70.7%; $p<0.01$) and total cholesterol levels (42%; $p<0.01$), while increasing HDL-cholesterol levels (100.8%; $p<0.05$) compared to pregnant diabetic control.

Table 4. Maternal biochemical parameters in pregnant normal and untreated and treated diabetic rats

	Treatments					
**Biomarkers**	**NC (n=9)**	**DC (n=6)**	**Gli (n=5)**	**AoAE50 (n=8)**	**AoAE100 (n=5)**	**AoAE200 (n=7)**
**ALT (U/L)**	30.1 ± 5.4	$56.8 \pm 9.2^{*}$	$27.5 \pm 6.9^{\alpha}$	33.0 ± 7.7	$32.9 \pm 4.3^{\alpha}$	40.7 ± 2.4
**AST (U/L)**	106.0 ± 16.9	$281.4 \pm 31.2^{***}$	$174.2 \pm 25.2^{\alpha}$	$144.4 \pm 14.9^{\alpha\alpha}$	$180.0 \pm 10.7^{\alpha}$	$149.7 \pm 17.2^{\alpha\alpha}$
**AST/ALT**	2.33 ± 1.05	$9.91 \pm 1.65^{***}$	$2.90 \pm 0.56^{\alpha\alpha}$	$4.37 \pm 0.43^{\alpha\alpha}$	$3.46 \pm 0.37^{\alpha\alpha\alpha}$	$4.29 \pm 0.33^{\alpha\alpha}$
**Total Proteins (g/L)**	3.3 ± 0.2	3.1 ± 0.2	3.6 ± 0.3	3.6 ± 0.3	3.5 ± 0.2	$4.4 \pm 0.2^{**\alpha\alpha}$
**TriGlycerids (mg/dl)**	133.1 ± 9.5	$192.5 \pm 9.9^{**}$	153.5 ± 16.7	$123.1 \pm 11.0^{\alpha\alpha}$	$128.7 \pm 4.4^{\alpha}$	$146.3 \pm 6.8^{\alpha}$
**Total Cholest. (mg/dl)**	65.22 ± 5.28	$109.0 \pm 8.4^{*}$	$63.23 \pm 6.71^{\alpha\alpha}$	$69.75 \pm 9.27^{\alpha}$	$63.28 \pm 7.78^{\alpha\alpha}$	$60.25 \pm 3.05^{\alpha\alpha\alpha}$
**HDL-Cholest. (mg/dl)**	17.15 ± 3.17	$7.29 \pm 0.11^{**}$	$14.64 \pm 1.20^{\alpha}$	$14.54 \pm 1.14^{\alpha\alpha}$	$18.00 \pm 2.65^{\alpha}$	$26.16 \pm 2.34^{*\alpha\beta}$
**LDL-Cholest. (mg/dl)**	80.49 ± 9.62	$116.5 \pm 7.14^{*}$	96.78 ± 11.15	$67.59 \pm 10.2^{\alpha}$	77.19 ± 14.52	102.5 ± 5.76

Values are expressed as mean ± SEM; n = 5-9 per group; $^{*}p<0.05$, $^{**}p<0.01$, $^{***}p<0.001$= significant difference compared to NC; $^{\alpha}p<0.05$, $^{\alpha\alpha}p<0.01$= significant difference compared to DC; $^{\beta}p<0.05$= significant difference from Gli; NC = normal control; DC = diabetic control; Gli = Pregnant diabetic rats treated with glibenclamide; AoAE 50, AoAE 100 and AoAE 200 = Pregnant diabetic rats treated with *Angylocalyx oligophyllus* aqueous extract at the doses of 50, 100 and 200 mg/kg, respectively; ALT = ALanine Transaminase; AST = Aspartate Transaminase; Total Cholest. = Total Cholesterol, HDL-Cholest. = High-Density Lipoprotein cholesterol; LDL-Cholest. = Low-Density Lipoprotein cholesterol.

Effect of the plant extract on histomorphological changes in maternal pancreas

Microscopic examination of pancreatic sections from pregnant normal control rats "Fig 5A" showed a regular appearance of islets of Langerhans resembling a rounded or oval pale-coloured area not encapsulated within the pancreatic lobules, which are made up of groups of cells arranged in irregular, branched and anastomosing cords separated by blood capillaries. However, the pregnant diabetic rats pancreatic sections showed atrophied and shrunken islets of Langerhans. On the other hand, *A. oligophyllus* extract and glibenclamide increased islet volume with near-normal contours showing cellular restoration in treated pregnant diabetic rats "Fig 5A". The "Fig 5B" shows that pre-gestational diabetes significantly reduced ($p<0.05$) the number of pancreatic cells by 35.49% compared to normal control. The pancreatic cells number of diabetic rats treated with the plant extract doses of 50, 100 and 200 mg/kg and glibenclamide significantly increased ($p<0.05$- $p<0.01$), respectively by 58.44%, 57.96%, 42.80% and 54.66%, compared to diabetic control.

Fig 5A. Microphotography of pancreas sections (haematoxylin-eosin x100) from pregnant normal and untreated and treated diabetic rats

NC = normal control; DC = diabetic control; Gli = Pregnant diabetic rats treated with glibenclamide; AoAE 50, AoAE 100 and AoAE 200 = Pregnant diabetic rats treated with *Angylocalyx oligophyllus* aqueous extract at the doses of 50, 100 and 200 mg/kg, respectively; exP = exocrine pancreas; enP = endrocrine pancreas, Red arrow = reduced diameter; Black arrow = normal diameter.

Fig 5B. Histomorphometrical data of pancreas sections from pregnant normal and untreated and treated diabetic rats

Values are expressed as mean ± SEM; n = 5-9 per group; $^{*}p<0.05$= significant difference compared to NC; $^{\alpha}p<0.05$, $^{\alpha\alpha}p<0.01$= significant difference compared to DC; NC = normal control; DC = diabetic control; Gli =

Pregnant diabetic rats treated with glibenclamide; AoAE 50, AoAE 100 and AoAE 200 = Pregnant diabetic rats treated with *Angylocalyx oligophyllus* aqueous extract at the doses of 50, 100 and 200 mg/kg, respectively.

Effects of the plant extract on maternal reproductive parameters

Maternal reproductive parameters registered at the 20th day of pregnancy are shown in “Table 5”. Overall, fertilization and pregnancy outcomes presented interesting findings in this study. In terms of fertilization, a high fertilization rate of 93.5% was observed among the 77 female rats (10 normal and 67 diabetic) mated. This included all 10 normal females and 62 diabetic females. A small proportion (6.5% or 5 total female rats), all of whom were diabetic, remained unfertilized after 15 days of mating and were categorized as infertile.

For pregnancy outcomes (among the fertilized population), 51.9% (40 out of the 77 total, or 9 normal and 31 diabetic) achieved an effective pregnancy. Intriguingly, 41.6% of the fertilized rats (32 total, or 1 normal and 31 diabetic) did not develop a pregnancy 12 days post-fertilization, highlighting a significant difference in pregnancy success, particularly among the diabetic group.

Furthermore, statistical analysis in each experimental group revealed that the coexisting adverse effects of pregnancy and pregestational diabetes in pregnant diabetic control rats, when compared to pregnant normal control rats, led to significant reductions ($p<0.05-p<0.01$) in gestation percentage (66.67%), pre-implantation loss (28.25%), corpora lutea (53.96%), live-born fetus numbers (47.95%), and uterine horn mass (30.26%). Additionally, there were significant increases ($p<0.05$) in post-implantation losses (1482% or ≈ 15.82-fold), resorptions (800% or ≈ 9-fold), and the number of stillborn fetuses (8) in the pregnant diabetic control group compared to the pregnant normal rat group.

Administration of *A. oligophyllus* leaves aqueous extract to pregnant diabetic rats increased the gestation percentage, significantly ($p<0.05$) by 2.67 and 2.33-fold (166.67%, and 133.33%) at doses of 50 and 200 mg/kg respectively, and non-significantly by 1.67 (66.67%)

at 100 mg/kg dose extract. The plant extract also increased but non-significantly live-born fetus numbers by 52.63%, 21.05%, and 50% at respective doses of 50, 100 and 200 mg/kg. However, it significantly increased ($p<0.05$) corpora lutea numbers by 66.39%, 35.25%, and 35.25% at these same respective doses, all compared to pregnant diabetic control rats. In pregnant diabetic rats, only the 100 mg/kg dose of the plant extract significantly reduced the pre-implantation loss (5.86%; $p<0.01$) compared to pregnant diabetic control rats. Interestingly, the plant extract at all tested doses (50-200 mg/kg) reduced the post-implantation loss respectively by 90.48%, 89.11% and 50.19%, and prevented the resorptions and fetal death, as compared to pregnant diabetic control rats. Furthermore, the plant extract increased the uterine horn mass at all doses but significantly at a dose of 50 mg/kg (43.38%; $p<0.05$) compared to pregnant diabetic control rats, and at doses of 50 mg/kg (56%; $p<0.01$) and 200 mg/kg (29.7%; $p<0.05$) compared to glibenclamide-treated rats group.

In glibenclamide-treated pregnant diabetic rats, compared to pregnant diabetic control, there were reduced pre-implantation loss (5.42%; $p<0.01$), post-implantation loss (58.74%), resorptions (77.78%), and live fetus numbers (7.89%). Additionally, no stillborn fetuses were recorded, and there was an increased gestation percentage (11.67%) and corpora lutea number (38.52%).

Table 5. Treatment-related variations in reproductive parameters of pregnant rats

Parameters	Groups					
	NC (n=9)	DC (n=6)	Gli (n=5)	AoAE50 (n=8)	AoAE100 (n=5)	AoAE200 (n=7)
**Pregnant (Day 0) (N)**	**10**	**20**	**12**	**10**	**10**	**10**
**Effectively pregnant (Day 20) (N)**	**9**	**6**	**5**	**8**	**5**	**7**
% Pregnancy	90	30**	41.67*	80 $^{\alpha}$	50	70 $^{\alpha}$
Gestational index (%)						
Mean ± SEM	85.0 ± 5.0	35.0 ± 6.4**	55.6 ± 6.3	80.0 ± 4.5 $^{\alpha}$	76.7 ± 7.1 $^{\alpha}$	80.0 ± 7.6$^{\alpha}$
**Corpora lutea (N)**	**265**	**122** **	**169**	**203** $^{\alpha}$	**165** $^{\alpha}$	**165** $^{\alpha}$
Mean ± SEM	33.13 ± 2.62	20.33 ± 2.32	33.80 ± 2.22	29.0 ± 2.14	33.0 ± 1,95	27.0 ± 3.55
**Implantation (N)**	**74**	**48**	**39**	**59**	**38**	**63**
Mean ± SEM	9.3 ± 0.9	8.0 ± 0,5	7.8 ± 0,5	8.4 ± 0.4	7.6 ± 3.1	9.0 ± 0.6
**Pre-implantation loss (%)**	**566.1**	**406.2***	**384.2** $^{\alpha\alpha}$	**487.9**	**382.4** $^{\alpha\alpha}$	**385.6**
Mean ± SEM	70.8 ± 3.5	58.0 ± 6.4	76.8 ± 0.9	69.7 ± 3.1	76.5 ± 3.1	64.3 ± 5.8
**Post-implantation loss (%)**	**8.3**	**131.3***	**54.17**	**12.5**$^{\beta}$	**14.3**	**65.4**
Mean ± SEM	1.8 ± 1.8	18.8 ± 13.7	10.8 ± 2.8	1.79 ± 1.8	2.9 ± 2.9	9.3 ± 4.7
**Resorptions (N)**	**1**	**9***	**2**	**0**	**0**	**1**
Mean ± SEM	0.13 ± 0.13	1.28 ± 1.2	0.33 ± 0.21	0.00 ± 0.00	0.00 ± 0.00	0.14 ± 0.14
**Fetal survival index (%)**						
Mean ± SEM	95.2 ± 7.5	82.8 ± 13.9	91.7 ± 3.5	94.8 ± 2.4	97.6 ± 2.4	89.9 ± 5.3
**Live fetuses (N)**	**73**	**38***	**35**	**58**	**46**	**57**
Mean ± SEM	9.1 ± 0.8	6.3 ± 1.3	7.0 ± 0.6	8.3 ± 0.4	7.7 ± 0.7	8.1 ± 0.7
**Dead fetuses (N)**	**0**	**8***	**0**	**0**	**0**	**0**
Mean ± SEM	0.0 ± 0.0	1.1 ± 1.4	0.0 ± 0.0	0.0 ± 0.0	0.0 ± 0.0	0.0 ± 0.0
**Uterine horn (g)**	**39.0 ± 2.2**	**27.2 ± 3.5***	**24.7 ± 1.0**	**39.0 ± 2.9**$^{\alpha\beta\beta}$	**29.2 ± 2.6**	**32.0 ± 1.9**$^{\beta}$
**Placenta (g)**	**3.50 ± 0.27**	**3.79 ± 0.38**	**3.41 ± 0.12**	**3.66 ± 0.17**	**3.41 ± 0.46**	**3.90 ± 0.50**

Values are expressed as mean ± SEM; n =5-9 per group; $^{*}p<0.05$, $^{**}p<0.01$= significant difference compared to NC; $^{\alpha}p<0.05$, $^{\alpha\alpha}p<0.01$= significant difference compared to DC; NC = normal control; DC = diabetic control; Gli = Pregnant diabetic rats treated with glibenclamide; AoAE 50, AoAE 100 and AoAE 200 = Pregnant diabetic rats treated with *Angylocalyx oligophyllus* aqueous extract at the doses of 50, 100 and 200 mg/kg, respectively.

Effects of the plant extract on maternal serum 17-β-Estradiol and Progesterone levels

The “Fig 6A” shows that serum 17-β-estradiol levels significantly decreased by 27.54% ($p<0.01$) in pregnant diabetic control rats compared to pregnant normal control. The *A. oligophyllus* leaves aqueous extract at all doses tested significantly increased ($p<0.01$) serum 17-β-estradiol levels with maximum percentages observed at doses of 50 mg/kg (50.38%) and 200 mg/kg (40.14%) compared to pregnant diabetic control.

In the other hand, slight non-significant variations in serum progesterone levels were recorded between groups “Fig 6B”, with a 1.8% decrease in pregnant diabetic control compared to pregnant normal control, and enhancements of 8.5%, 3.1% and 5.5% observed at the plant extract doses of 50, 100 and 200 mg/kg compared to pregnant diabetic control. The glibenclamide induced a non-significant 22.94% increase in serum 17-β-estradiol level, but did not vary the progesterone level compared to pregnant diabetic control.

Fig 6. Serum17-β-estradiol (A) and progesterone (B) levels in pregnant normal and untreated and treated diabetic rats

Values are expressed as mean ± SEM; n = 5-9 per group; $^{*}p<0.05$, $^{**}p<0.01$= significant difference compared to NC; $^{\alpha\alpha}p<0.01$= significant difference compared to DC; NC = normal control; DC = diabetic control; Gli = Pregnant diabetic rats treated with glibenclamide; AoAE 50, AoAE 100 and AoAE 200 = Pregnant diabetic rats treated with *Angylocalyx oligophyllus* aqueous extract at the doses of 50, 100 and 200 mg/kg, respectively.

Effects of the aqueous extract of *A. oligophyllus* leaves on fetal parameters

Effects of the plant extract on fetal weight and external morphological characteristics

“Table 6” shows the equivalent fetuses weight percentages of gestational age between experimental groups. The pups from diabetic control rats had a low weight ($p<0.05$) compared to those from normoglycaemic or normal control rats. In addition, the AGA (adequate fetal weight for gestational age) percentage decreased ($p<0.05$), whereas percentages of SGA (small fetal weight for gestational age) ($p<0.05$) and LGA (larger fetal weight for gestational age) increased in diabetic control group compared to normal control. However, the percentages of SGA and AGA of the diabetic control group were almost equal (40.81% vs. 48.98%).

The *A. oligophyllus* extract and glibenclamide normalized the pups body weights ($p<0.05$-$p<0.01$). Furthermore, although the SGA percentages were very low in pregnant diabetic rats treated with *A. oligophyllus*, the distribution of pups in these groups between AGA and LGA was such that the AGA increased and LGA decreased in an inversely dose-dependent manner, with significant values ($p<0.05$) at the 50 mg/kg dose, AGA and LGA percentages being almost equal in the 200 mg/kg dose (50% vs. 46.43%), all compared to the diabetic controls. Moreover, the plant extract doses of 100 and 200 mg/kg showed high percentages of LGA compared to normal and diabetic controls. Glibenclamide also increased the percentage of pups with AGA and reduced the percentages of SGA and LGA, compared to the diabetic controls.

The “Fig 7” shows that maternal diabetes induced caudal regression and hematomas in fetuses. The fetuses from *A. oligophyllus* aqueous extract-treated diabetic rats displayed a more developed tail and reduced haematomas compared to those from diabetic control rats.

Table 6. Variation in fetal mass and percentages of fetal weight corresponding to gestational age, according to treatments

Groups	Parameters			
	Fetal body weight (g)	SGA (%)	AGA (%)	LGA (%)
**NC** (n=9)	3.2 ± 0.2	0.0	92.65	7.35
**DC** (n=6)	2.1 ± 0.2*	40.81*	48.98*	10.20
**Gli** (n=5)	3.2 ± 0.4$^{\alpha}$	22.5	90	7.5
**AoAE50** (n=8)	3.1 ± 0.2 $^{\alpha\alpha}$	0.0 $^{\alpha}$	97.96$^{\alpha}$	2.04
**AoAE100** (n=5)	2.9 ± 0.5	4.35	78.26	17.39
**AoAE200** (n=7)	3.3 ± 0.4 $^{\alpha}$	3.57	50	46.43$^{\alpha}$

Values are expressed as mean ± SEM; n = 5-9 per group; *p<0.05, = significant difference compared to NC; $^{\alpha}$p<0.05, $^{\alpha\alpha}$p<0.01= significant difference compared to DC; NC= Normal control; DC = diabetic control; Gli = Pregnant diabetic rats treated with glibenclamide; AoAE50, AoAE100 and AoAE200 = Pregnant diabetic rats treated with *Angylocalyx oligophyllus* aqueous extract at the doses of 50, 100 and 200 mg/kg, respectively. SGA= Small Gestational Age; AGA= Adequate Gestational Age; LGA= Large Gestational Age.

Fig 7. External morphological malformations in fetuses from pregnant normal and untreated and treated diabetic rats

n = 5-9; NC= Normal control; DC = diabetic control; Gli = Pregnant diabetic rats treated with glibenclamide; AoAE 50, AoAE 100 and AoAE 200 = Pregnant diabetic rats treated with *Angylocalyx oligophyllus* aqueous extract at the doses of 50, 100 and 200 mg/kg, respectively; CR = Caudal Regression; Hem = Haematomas

Effect of the plant extract on placental efficiency

The “Fig 8” shows that the placental efficiency significantly decreased by 40.65% (p<0.01) in pregnant diabetic control rats compared to pregnant normal control. The *A. oligophyllus* aqueous extract doses of 50 and 200 mg/kg increased the placental efficiency respectively by

55.12% ($p<0.01$) and 55.04% ($p<0.05$), while glibenclamide and the plant extract dose of 100 mg/kg induced a non-significant increase ($p>0.05$) of 40.23% and 23.01% respectively, all compared to pregnant diabetic control "Fig 8".

Fig 8. Effect of *A. oligophyllus* on placental efficiency

Values are expressed as mean ± SEM; n = 5-9 per group; $^{*}p<0.05$, = significant difference compared to NC; $^{\alpha}p<0.05$, $^{\alpha\alpha}p<0.01$= significant difference compared to DC; $^{\beta\beta}p<0.01$= significant difference compared to Glib; NC= Normal control; DC = diabetic control; Gli = Pregnant diabetic rats treated with glibenclamide; AoAE 50, AoAE 100 and AoAE 200 = Pregnant diabetic rats treated with *Angylocalyx oligophyllus* aqueous extract at the doses of 50, 100 and 200 mg/kg, respectively.

Effect of the plant extract on histomorphological changes in placenta

The "Fig 9" shows microphotographs of rat placental sections. Placental sections from normal rats showed glycogenic cells with glycogen deposits (Gly), trophoblastic cells (TC), giant trophoblastic cells (gTC) and a spongiotrophoblast (ST) in the basal zone. Compared to normal rats, diabetic rats showed no glycogenic cells but a very marked presence of haematomas (Hem). The *A. oligophyllus* leaves aqueous extract and glibenclamide corrected these alterations by reducing the surface area of trophoblastic haematomas. In addition, the plant extract and glibenclamide induced glycogenic cells regeneration with glycogen deposits.

Fig 9. Microphotography of placenta sections (haematoxylin-eosin x100) from pregnant normal and untreated and treated diabetic rats

n = 5-9; NC = normal control; DC = diabetic control; Gli = Pregnant diabetic rats treated with glibenclamide; AoAE 50, AoAE 100 and AoAE 200 = Pregnant diabetic rats treated with *Angylocalyx oligophyllus* aqueous extract at the doses of 50, 100 and 200 mg/kg, respectively; Gly = Glycogen, Hem = Haematoma, Bv= Blood vessels, Agly = Absence of glycogen, ST = Spongiotrophoblast, TC = Trophoblastic cells, gTC = giant Trophoblastic cells; LZ = Labyrinth Zone; JZ= Junctional Zone; D = Decidua.

Discussion

Pregestational diabetes is one of the most common complications in pregnancy with serious consequences for the mother and her offspring [6]. In this study, an animal model of pregestational diabetes was established to determine the effect of hyperglyceamia on metabolism, maternal reproductive performance and fetal morphological parameters during pregnancy, and the *Angylocalyx oligophyllus* leaves aqueous extract’s impact.

Body weight is one and the first of the most important physiological parameters during pregnancy providing information on fetal growth and mother health due to its direct relationship with nutrient metabolism [49]. Indeed, pregnancy is a period in a woman's life during which many physiological changes occur, including an inevitable and necessary weight gain. Weight gain during pregnancy is the result of the adaptation of the pregnant woman's body to the fetus that inhabits it, through physical, physiological and hormonal changes and is due to three rather equal causes: Increase in fat, increase in body mass by the fetus, increase in volume of water, blood, organs. During pregnancy, the maternal organism thanks to pregnancy hormones stores fats necessary and essential to support the development of the fetus. In addition, the growth of the fetus, placenta and amniotic fluid, water retention and increased blood volume (to provide sufficient nutrients and oxygen to the fetus through the blood), the increase in the volume of the uterus and mammary glands, contribute to a weight gain of the pregnant woman. These parameters or at least some can be modified in the event of coexisting pathologies during pregnancy such as pregestational diabetes, thus affecting the proper development of pregnancy [50]. Present results showed progressive lowed weight gain associated with reduced abdominal and peri-ovarian fat mass, small fetal mass for gestational age, reduced uterine horn mass and number of born fetuses, but high liver and kidneys relative masses in pregnant type 1 diabetic control rats compared with pregnant

normal control rats, although placental mass did not change between groups. These results have been also reported in many experimental studies [51,52,53], and were justified by the altered nutrients metabolism (glucose, lipid and proteins) in pregnant diabetic rats. Giudetti [54] suggested that substantial weight loss would be related to the rate of lipid absorption in adipose tissue. Seydoux et al. [55] had already reported that streptozotocin alters metabolism by atrophy of the abdominal fat tissue. Streptozotocin-induced type 1 diabetes is due to insulinopenia, which would have contributed to a state of denutrition and/or malnutrition and altered nutrients metabolism, decreasing the fat mass and reducing the body weight gain both in the mother and her offspring.

The administration of *A. oligophyllus* leaves aqueous extract enhanced body weight gain, abdominal and peri-ovarian fat masses, and increased the uterine horn mass, the number of fetuses and the fetal weight in plant-treated pregnant diabetic rats, probably by improving nutrients (glucose, lipid and protein) levels and metabolism. The plant extract promoting effect on both mother and pups body weight gain may be justified by the increase in fat masses, which would itself suggest a lipogenic effect of this plant extract, but also by a nutritional value of the plant as suggested by Tenezogang et al. [24] which reported a weight gain in pregnant normoglycaemic rats receiving the *A. oligophyllus* leaves aqueous extract during pregnancy. Similarly, several other plants such as *Hibiscus rosa-sinensis* [56], *Olea europea* [52] and *Garcinia kola* [57] are reported enhancing weight gain in pregnant diabetic rats. The increased number of fetuses in plant extract-treated pregnant diabetic rats contributed to increasing mother’s weight gain, and may be linked to the plant's beneficial effects on diabetes control and its adverse effects on pregnancy.

During pregnancy with type 1 diabetes, carbohydrate metabolism is doubly affected by hyperglycemia related to insulinopenia and by insulin resistance that naturally develops during pregnancy. The oral glucose tolerance test is recommended for better glycemic control

during pregnancy, as this test would be a better indicator to determine the degree of glucose intolerance during gestation, and it expresses the capacity of β cells to secrete insulin [58]. Improved glucose intolerance during gestation would then indicate better stimulability of insulin release. Pregnant diabetic control rats showed chronic hyperglycemia associated with glucose intolerance and insulin resistance both characterized by significant increases in the areas under the blood glucose curves compared to normal pregnant rats. The hyperglycemia could be explained by the reduction in the surface area of the endocrine pancreas (small pancreatic islets) and the total number of pancreatic cells, as observed in pancreas sections from diabetic control rats, reflecting cellular necrosis. Glucose intolerance would therefore result from insulinopenia due to pancreatic cells number reduction, thus leading to a decrease in the response to insulin [59]. This observation has been reported by several other authors [52,56,60,61].

The *A. olligophyllus* leaves aqueous extract administered during pregnancy significantly reduced blood glucose levels in pregnant diabetic rats on gestational days 15 (50 mg/kg) and 20 (50 and 200 mg/kg) compared to pregnant diabetic controls, suggesting the presence of bioactive metabolites (polyphenols, flavonoids, tanins, free quinone, alkaloids, saponins etc.) already reported to have insulinotropic effects [62,63,43]. The plant extract would also have acted by inhibiting salivary and/or pancreatic α-amylase activities, as it is here observed that the plant extract inhibited the α-amylase activity in vitro, thereby reducing the intestinal glucose absorption and improving glucose tolerance; this extract would also have affected carbohydrate metabolism by improving liver/muscle glucose absorption [64] and stimulating the production of incretins (GLP-1) which have an effect on insulin release. Moreover, the histology of pancreatic sections showing an increase in the pancreatic islets size and a normalization of the total number of pancreatic cells in the plant extract-treated pregnant diabetic rats, suggests that the *A. olligophyllus* extract may prevent pancreatic cell damage

and/or stimulate the regeneration of pancreatic cells and thus of β-cells, and explains the beneficial effects of this plant on glycemia, glucose tolerance and insulin sensitivity. Flavonols which presence was revealed in the *A. olligophyllus* leaves aqueous extract could be partly responsible for these effects, as Coshun et al. [65] reported that a flavonol compound, quercetin, prevents β-cell damage associated with glucotoxicity. Furthermore, the increase in adipose tissue recorded in diabetic rats receiving the *A. oligophyllus* leaves aqueous extract would also explain the improvement of carbohydrate profile and the regeneration of pancreatic β-cells by this plant extract. Ikemoto et al. [66] reported that adipose tissue from a type 1 diabetic is a result or evidence of regeneration of insulin-producing cells. These observations have also been reported with extracts from *Ortosipon stamineus* [60] and *Ganoderma lucidum* [61].

The high physiologically modification of energy metabolism for fetal development and growth during pregnancy also influences every mother's body organ so that in case of pregestational diabetes, these are even more affected due to impaired glycemic adjustment. Liver and kidneys have been reported being the mostly affected organs by energy metabolism [67,68,69]. However, transaminases ALT and AST are essential tool enzymes (biomarkers) for assessing liver function and diseases, whose blood increase indicates hepatic, but also renal and muscular disorders [68,70,71]. Moreover, since the liver is also the site of lipid and protein metabolism, their abnormal serum levels would also explain impaired liver function. The increased liver and kidney relative masses recorded in pregnant diabetic rats would suggest a hepatomegaly and a kidney hypertrophy respectively. The observed hepatomegaly would be linked to fat accumulation suggesting increased production of hepatic triglycerides, and/or to hepatic hyperplasia indicating liver injury. Regnell and Lernmark [72] reported that hyperglycaemia leads to the production of triglycerides in the hepatocytes, which would be responsible for the liver fatty deposits. Thus, since lipid metabolism plays a major role in

maternal and fetal energy requirements, a pregnant diabetic woman is at increased risk of dyslipidemia [73,74]. The impaired lipid profile (high triglyceridemia, high total and LDL–cholesterolemia, low HDL–cholesterolemia) recorded in pregnant diabetic control rats, confirms the hepatic lipid hyperproduction suggested above. The associated reduced protein levels also registered in pregnant diabetic control rats are probably due to an increase in proteolytic activity and neoglucogenesis, and a decrease in amino-acid absorption and in protein biosynthesis and absorption [75,76]. Furthermore, serum transaminase (AST/ALT) levels usually are decreased slightly or do not change significantly during pregnancy, and can be used to assess liver damage during pregnancy [77]. Thus, the increase in serum transaminase (AST/ALT) activities observed in pregnant diabetic control rats are mainly due to diabetes. This indicates or confirms the hepatocellular injury or necrosis, and could be explained by fatty infiltration related to the disruption of the β-oxidation system in hepatic cell mitochondria and/or an increase in gluconeogenesis. Stefan et al. [78] reported that in situations of hyperglycemia, the liver no longer detects glucose availability and produces more of it via several enzymes involved in its synthesis (glucose-6-phosphate dehydrogenase, hexokinase, glucokinase).

The kidney hypertrophy, on the other hand, would be related to increased renal vasculature, interstitial volume and dead space, significant water and glucose reabsorption [79], due both to diabetes and pregnancy, as it is reported that hyperglyceamia is closely related to increased kidney size [80]. This may also indicate possible apparent high blood pressure.

Although the *A. oligophyllus* leaves aqueous extract did not decrease the high relative masses of liver and kidney, it significantly improved lipid profile, protein and transaminases levels in plant extract-treated pregnant diabetic rats compared to pregnant diabetic control, limiting tissues damages. Mohammed et al. [52] also reported hypolipidemic effect of olive leaves extract in pregnant diabetic rats. The *A. oligophyllus* extract would have increased the

expression of lipoprotein lipase (LPL), by activating PPAR, or by inhibiting the activities of one or more enzymes involved in cholesterol metabolism, increasing the blood level of LPL, notably ACAT 1 and 2, IBAT, CETP, MTP and/or squalene synthase, which would have promoted the degradation and/or decrease in the synthesis or transport of triglycerides and cholesterol in the digestive tract, blood, liver and adipose tissue. Interestingly, the 52.54% increase in HDL-cholesterol levels observed at the plant extract dose of 200 mg/kg compared to pregnant normal control rats indicates a probable beneficial effect against the risk of developing atherosclerosis and consequently a reduction in cardiovascular risk, since it is commonly accepted that an increase in HDL-cholesterol of approximately 30% would be beneficial against the risk of developing atherosclerosis [81]. The significant blood protein levels increase obtained at a plant extract dose of 200 mg/kg could be compensatory for the effects of oxidative stress, attributed to the steroids and proteins contained in the plant extract, and/or related to insulin secretion. Moreover, the reducing effects of the plant extract on serum ALT and AST levels suggests a hepatoprotective effect of the plant, which could be explained by the plant’s ability to protect hepatocyte lipid membranes by preventing the production and/or neutralising free radicals, thus improving liver function.

Impaired reproductive performance is a well-known consequence of diabetic syndrome in many mammalian species, including humans. Pregnant diabetic control rats showed decreased pregnancy percentage, corpora lutea, implantation sites, preimplantation loss, and increased resorptions, postimplantation loss, and dead fetuses compared to pregnant normal rats. Many studies have shown that mild hyperglycemia leads to impaired maternal reproductive performance, spontaneous abortions, and perinatal deaths. Similarly, this hyperglycemia associated with oxidative stress impairs embryogenesis from the pre-implantation phase and fetal development [82,83,84]. *A. oligophyllus* extract at all doses significantly increased pregnancy percentage and corpora lutea number compared to pregnant diabetic control rats.

This increase in corpora lutea number is linked to the hyperglycemia decrease and the islet β-cells mass increase. The plant extract would also have stimulated PPARγ (peroxisome proliferator-activated receptor γ), which is highly expressed in ovaries and placenta and contributes to the production of gestational hormones (estradiol and progesterone secretion compatible with embryonic implantation) [85]. The increase of serum 17-β-estradiol levels in pregnant diabetic rats treated with all plant extract doses compared to pregnant diabetic control rats would explain these plant effects on the ovary and even the placenta, contributing to fetal development.

Fetal weight and morphology, placental weights, placental efficiency and placental anatomo-histology are good indicators of the quality of fetal-maternal exchanges during gestation [86,87]. The placenta constitutes a functional unit between mother and child, playing a major role in maternal-placental transfer and in the fetal utilization of nutrients and oxygen. Its growth in weight and size is correlated with that of the fetus. The correlation between growth retardation and placental abnormalities has been established. Bendix et al. [88] showed that placental alteration resulted in reduced fetal weight. Hyperglycemia leads to exhaustion of the functional fetal pancreas, with reduced insulin production impairing fetal growth, characterized by a very high percentage of SGA or microsomia [89]. This was highlighted in the present work by the fetal growth retardation (small weight for gestational age (SGA)) and congenital malformations (caudal/tail regression, sub-cutaneous hematoma) associated with placental injury and inefficiency observed in pregnant diabetic control rats, all probably due to a severe insulinopenia and consequent hyperglycemia affecting the well metabolic and energy activity control.

Pregnant diabetic rats receiving the plant extract doses produced predominantly fetuses with adequate weight for gestational age (AGA), which could be explained by an improvement in the structure and function of the pancreas, increasing insulin synthesis and insulin sensitivity

at the placental level. Moreover, the very marked presence of hematomas and the high glycogenic cells reduction observed in placental sections from pregnant diabetic control rats associated to reduced serum 17-β-estradiol levels are linked to adverse effects of diabetes and suggest maternal blood flow interruption, risks of spontaneous abortion and consequently impaired reproductive performance in these animals compare to pregnant normal control. However, the reduced surface area of trophoblastic hematomas associated to glycogenic cells regeneration with glycogen deposits observed in placental sections from plant extract-treated pregnant diabetic rats show that the *A. olligophyllus* improved reproductive performance by preventing spontaneous abortion due to diabetes. Sun et al. [90] reported that *Juglans regia* L. may have improved the weight of fetuses from diabetic mothers thanks to its antioxidant properties. *A. olligophyllus* extract may also have exerted all these effects, partly due to its numerous antioxidant properties, as observed in this study in the in vitro tests.

The overall beneficial effects of the *A. olligophyllus* leaves aqueous extract in pregnant diabetic rats could be attributed to the complexity of the synergistic, additive and/or antagonistic effects of the numerous bioactive secondary metabolites that constitute it, such as polyphenols, flavonoids, free quinones, tannins, alkaloids, saponins, terpenes, unsaturated sterols, carotenoids, etc. identified in this plant extract. Flavonoids are known for their numerous biological activities including strong antioxidant properties. Tannins bind proteins and enzymes, protecting against proteolysis and inhibiting enzymes, thus slowing down the absorption of nutrients including glucose in the digestive tract and also playing an antioxidant role. Monomeric, dimeric and polymeric tannin fractions extracted from *Raphia gentiliana* fruit powder have shown antihyperglycemic properties in mice and antiradical properties in vitro [91].

Conclusion

The present study revealed that diabetes negatively impacts fertilization, nidation and the course of pregnancy. However, aqueous extract from *A. oligophyllus* leaves, administered from day 1 of gestation, maintained pregnancy, improved feto-maternal outcomes, and thus improved reproduction in pregnant diabetic rats through the antidiabetic and antioxidant effects of its bioactive molecules. Although models used are reliable, and *A. oligophyllus* is used empirically for pregnancy improvement in case of diabetes, further studies are needed to translate these results to human being or other species.

Conflict of interests

The authors have not declared any conflict of interests.

Acknowledgements

This work did not receive funding. However, we would like to express our sincere thanks to the Alexander Von Humboldt Foundation for awarding the equipment grant to one of the authors which enabled part of this work to be carried out.

Supporting Information

SI1. PLOS ONE Humane Endpoints Checklist for PLOS ONE manuscript number: PONE-D-25-21592

SI2. ARRIVE Guidelines Checklist: Author Checklist - Full

Author contributions

Conceptualization: Christian T. Tenezogang, Marie C. Tchamadeu, Dieudonné L. Massoma

Data curation: Christian T. Tenezogang, Marie C. Tchamadeu, Dieudonné L. Massoma,

Formal analysis: Christian T. Tenezogang, Marie C. Tchamadeu, Sylvin B. Ateba, William N. Yousseu, Calvin Z. Bogning, Modeste Wankeu-Nya, Alain B. Dongmo, Dieudonné L. Massoma

Methodology: Christian T. Tenezogang, Marie C. Tchamadeu, Dieudonné L. Massoma, Pascal E. Owona

Investigation: Christian T. Tenezogang, Marie C. Tchamadeu, Wylliam N. Yousseu, Pascal E. Owona, Quelie N. Selakong, Calvin Z. Bogning, Armel-Kevin F. Pechi, Ahmadou Hassimatou

Supervision: Dieudonné L. Massoma, Marie C. Tchamadeu

Visualization: Christian T. Tenezogang, Marie C. Tchamadeu, Pascal E. Owona, Dieudonné L. Massoma

Validation: Marie C. Tchamadeu, Dieudonné L. Massoma

Writing – original draft: Christian T. Tenezogang, Marie C. Tchamadeu

Writing – review and editing: Christian T. Tenezogang, Marie C. Tchamadeu, Sylvin B. Ateba; Wylliam N. Yousseu, Modeste Wankeu-Nya, Alain B. Dongmo, Dieudonné L. Massoma

References

1. Araujo-Silva VC, Santos-Silva A, Lourenco AS, Barros-Barbosa CM, Moraes-Souza RQ, Soares TS. Congenital anomalies programmed by maternal diabetes and obesity on offspring of rats. Frontiers in Physiology. 2021; 12:701767. http://doi.org/ 10.3389/fphys.2021.701767

2. Jullien M, Assirlikian S, Jannot-Lamotte M-F. Diabètes et fertilité – Diabetes and fertility. Médecine de la Reproduction; Cairn.info. 2023; 3(25):206-216. https://doi.org/10.1684/mte.2023.0969

3. Ornoy A, Becker M, Weinstein-Fudim L, Ergaz Z. Diabetes during Pregnancy: A Maternal Disease Complicating the Course of Pregnancy with Long-Term Deleterious Effects on the

Offspring. A Clinical Review. International Journal of Molecular Sciences. 2021; 22(6): 2965. http//doi.org/ 10.3390/ijms22062965

4. Reitzle L, Heidemann C, Baumert J, Kaltheuner M, Adamczewski H, Icks A, Scheidt-Nave C. Pregnancy Complications in Women With Pregestational and Gestational Diabetes Mellitus. Deutsches Arzteblatt International. 2023;120(6):81-86. http//doi.org/10.3238/arztebl.m2022.0387

5. IDF (Internationnal Diabetes Federation). IDF Diabetes Atlas. 10th Edition, 2021, p141. www.diabetesatlas.org**.**

6. Meykiechel T, Bourcigaux N, Christin-Maitre S. Le diabète gestationnel: diagnostic et prise en charge - Gestational diabetes: Diagnosis and management. EM Consulte, Anesthésie et Réanimation; Société Française d'Anesthésie et de Réanimation (SFAR), Elsevier. 2023; 9(3):268-276. https://doi.org/10.1016/j.anrea.2023.05.002

7. Rodolaki K, Pergialiotis V, Iakovidou N, Boutsikou T, Iliodromiti Z and Kanaka-Gantenbein C. The impact of maternal diabetes on the future health and neurodevelopment of the offspring: a review of the evidence. Frontiers Endocrinology. 2023; 14:1125628. http//doi.org/10.3389/fendo.2023.1125628

8. Fleming TP, Sun C, Denisenko O, Caetano L, Aljahdali A, Gould JM, Khurana P. Environmental Exposures around Conception: Developmental Pathways Leading to Lifetime Disease Risk. International Journal Environmental Research and Public Health. 2021;18(17):9380. http//doi.org/10.3390/ijerph18179380

9. Wyman A, Pinto AB, Sheridan R, Moley KH. One-cell zygote transfer from diabetic to nondiabetic mouse results in congenital malformations and growth retardation in offspring. Endocrinology. 2008; 149:466 – 469. http//doi.org/ 10.1210/en.2007-1273

10. Negrato CA, Mattar R, Gomes MB. Adverse pregnancy outcomes in women with diabetes. Diabetology & Metabolic Syndrome. 2012; 4:41.

http://www.dmsjournal.com/content/4/1/41

11. Bouché C, Bismuth E, Catherine C, Lepercq J, Lubin V, Rouge D, Vambergue A. Management of pregnancy in type 1 diabetic women. Expert-consensus from société Francophone du Diabète (SFD) 2010. Extrait de Médecine des maladies métaboliques. 2011; 5(1): 7-19. https://doi.org/10.1016/S1957-2557(11)70050-5

12. Capobianco G, Gulotta A, Tupponi G, Dessole F, Pola M, Virdis G, Sotgiu, G. Materno-Fetal and Neonatal Complications of Diabetes in Pregnancy: A Retrospective Study. Journal of Clinical Medicine. 2020; 9(9): 2707.

13. Temple RC, Aldridge VJ, Murphy HR. Prepregnancy care and pregnancy outcomes in women with type 1 diabetes. Diabetes Care. 2006; 29:1744-9. https://doi.org/10.2337/dc05-2265

14. Chabbert-Buffet N. Diabète de type 1 et fertilité - Type 1 diabetes and fertility. Médecine des Maladies Métaboliques. 2021; 15(4):364-368. https://doi.org/10.1016/j.mmm.2021.04.009

15. Vargas R, Repke JT, Ural SH. Type 1 Diabetes Mellitus and Pregnancy. Reviews in Obstetrics and Gynecology. 2010; 3(3):92-100. doi: 10.3909/riog0114

16. Brown MA, Magee LA, Kenny LC, Karumanchi SA, McCarthy FP, Saito S, Hall DR, Warren CE, Adoyi G, Ishaku S. International Society for the Study of Hypertension in Pregnancy (ISSHP): The hypertensive disorders of pregnancy: ISSHP classification, diagnosis & management recommendations for international practice. Pregnancy Hypertens. 2018; 13:291-310. http//doi.org/ 10.1161/HYPERTENSIONAHA.117.10803

17. Romero FT, Sonia C, Carlos LP, López NN, Román GS, Fernández G. Protective effect of NeuroEPO on the reproduction of diabetic rats. [Internet]. Amsterdam: Mendeley Data. 2022; 1. http//doi.org/10.17632/dvk9rtwccy

18. Kopylov AT, Papysheva O, Gribova I, Kotaysch G, Kharitonova L, Mayatskaya T, Morozov SG. Molecular pathophysiology of diabetes mellitus during pregnancy with antenatal complications. Scientific Reports. 2020; 10(1):19614. http//doi.org/10.1038/s41598-020-76689-9

19. Medjidova UM, Polukhova SM, Kazimova AU. Phytotherapy for gestational diabetes mellitus. SCIREA Journal of Medicine. 2023; 7(2):32-39. https://doi.org/10.54647/pm310227

20. Burkill HM. The useful plants of West Tropical Africa. 2nd Edition. Volume 3, Families J–L. Royal Botanic Gardens, Kew, Richmond, United Kingdom. 1995: 857. http://plants.jstor.org/upwta/2_580

21. Neuwinger HD. African traditional medicine: a dictionary of plant use and applications. Medpharm Scientific, Stuttgart, Germany. 2000: p589.

22. Wakeu KB, Jouda JB, Foudjo MG, Sidjui SL, Mkounga P, Lateef M, Shaiq AM, Wandji J, Djama MC. Oligoamide, a new lactam from the leaves of *Angylocalyx oligophyllus*. Natural Product Research. 2018: 1-5. http//doi.org/ 10.1080 / 14786419. 2018. 1483925

23. Asghar G, Sajad J. Streptozotocin as a tool for induction of rat models of diabetes: a practical guide. EXCLI Journal, 2023; 22:274-294. https://dx.doi.org/10.17179/excli2022-5720

24. Tenezogang TC, Tchamadeu MC, Bogning ZC, Emambo P, Wankeu NM, Dongmo AB, Massoma LD. Maternal-fetal repercussions of *Angylocalyx oligophyllus* leaves aqueous extract in pregnant rat. African Journal of Pharmacy and Pharmacology. 2022; 16(9): 143-152. https://doi.org/ 10.5897/AJPP2022.5317

25. OECD. OECD Guideline for the Testing of Chemicals, n^0 414. Pre-natal developmental toxicity study. Organization for Economic Cooperation and development Paris, France. https://doi.org/10.1787/20745788

26. Trease GE and Evans WC. Trease and Evan's Textbook of Pharmacognosy. 13th Edition. London: Cambridge University Press; 1989. 546p.

27. Gülçin I, Alici HA and Cesur M. Determination of in vitro antioxidant and radial scavenging activities of propofol. *Chemical and Pharmceutical Bulletin*, 2005; 53(3): 281-285. http://dx.doi.org/10.1248/cpb.53.281

28. Dohou N, Yamni K, Tahrouch S, Idrissi H, Badoc A, Gmira N. Screening phytochimique d'une endémique ibéromarocaine, Thymelaea lythroides. Bulletin de la Société Pharmaceutique de Bordeaux. 2003 ; 29: 233-239. https://doi.org/10.24310/abm.v29i0 .7221

29. Chun OK, Kim DO, Smith N, Schroeder D, Han JT, Lee CY. Daily consumption of phenolics and total antioxidant capacity from fruit and vegetable in the American diet. Journal of the science of Food and Agriculture. 2005; 85: 1715-1724. http://dx.doi.org/10.1002/jsfa.2176

30. Zhishen JT, Mengcheng and Jianming W. The determination of flavonoid contents in mulberry and their scavenging effects on superoxide radical. Food chemistry. 1999; 64: 555-559.

31. Apostolidis E and Lee C. *In vitro* potential of *Ascophyllum nodosum* phenolic antioxidant-mediated α-glucosidase and α-amylase inhibition. Journal Food Science. 2010; *75*, H97–H102.

32. Mensor LL, Menezes FS, Leitão GG, Reis AS, Santos TC, dos Coube CS, Leitão SG. Screening of Brazilian plant extracts for antioxidant activity by the use of DPPH free radical method. Phytotherapy Research. 2001; 15(2), 127–130. http//doi.org/10.1002/ptr.687

33. Re R, Pellegrini N, Proteggente A, Pannala A, Yang M, Rice-Evans C. Antioxidant activity applying an improved ABTS radical cation decolorization assay. Free radical biology medicine. 1999; 26 (9-10), 1231-1237

34. Rao MNA, Kunchandy E. Oxygen radical scavenging activity of curcumin. International Journal of Pharmaceutics. 1990; 58(3): 237–240. http//oi.org/10.1016/0378-5173(90)90201-e

35. Benzie IFF, Strain JJ. The ferric reducing ability of plasma as a measure of "antioxidant power" the FRAP assay. Analytical Biochemistry. 1996; 239, 70–76. https://doi.org/10.1006/abio.1996.0292.

36. Rees DA, Alcolado JC. Animal models of diabetes mellitus. Diabetes Medicine. 2005; 22:359–370

37. Eid FA, Shoman HH, Elnaga NAA, Abed El-Halim H. Effect of Olive Leaf Extract on the Kidney of Pregnant Diabetic Rats and Their Fetuses. International Journal of Advanced Research. 2014; 2 (11): 740-76. http//www.journalijar.com

38. Lehtoranta L. Fetal cardiac and placental function in rat model of maternal hyperglycemia and human type 1 diabetes pregnancies. Annales Universitatis Turkuensis tom. 1285/Medica-Odontologica/Turku. 2017, p1-101.

39. Damasceno DC, Volpato GT, Sinzato YK, Lima PH, Souza MS, Iessi IL, Kiss AC, Takaku M, Rudge MV, Calderon IM. Genotoxicity and fetal abnormality in streptozotocin-induced diabetic rats exposed to cigarette smoke prior to and during pregnancy. Experimental Clinical Endocrinology Diabetes. 2011; 119:549–553. http//doi.org/10.1055/s-0031-1277193

40. Kiss K, Brozik A, Kucsma N, Toth A, Gera M, Berry L, Vallentin A, Vial H, Vidal M, Szakacs G. Shifting the Paradigm: The Putative Mitochondrial Protein ABCB6 Resides

in the Lysosomes of Cells and in the Plasma Membrane of Erythrocytes. 2012; 7 (5): 0037378. http//www.plosone.org

41. Wilson ME and Ford SP. Comparative aspects of placental efficiency. Reproduction Supplement. 2001; 1(58):223–232.

42. Ngueguim TF, Esse EC, Dzeufiet DPD, Gounoue RK, Bilanda DC, Kamtchouing P, Dimo T. Oxidative palm oil and sucrose induced hyperglycemia in normal rats: effects of *Scleroclarya birrea* stem barks acqueous extract. BMC Complementary and Alternative Medicine, 2016 ; 16 (47):1-11.

43. Tchamadeu MC, Dzeufiet DPD, Blaes N, Girolami JP, Kamthouing P, Dimo T. Antidiabetic effects of aqueous and Dichloromethane/Methanol stem bark extracts of *Pterocarpus soyauxii* Taub (Papilionaceae) on Streptozotocin-induced Diabetic Rats. Pharmacognosy Research. 2017; 9(1): 80-86. https://doi.org/10.4103/0974-8490.199767

44. Diehl K-H, Hull R, Morton D, Pfister R, Rabemampianina Y, Smith D, Vidal J-M, Cor van de Vortenbosch. A good practice guide to the administration of substances and removal of blood, including routes and volumes. Journal of Applied Toxicology, 2001 ; 21: 15‑23.

45. Boers M, Nurmohamed MT, Doelman CJ, Lard LR, Verhoeven AC, Voskuyl AE, et al. Influence of glucocorticoids and disease activity on total and high density lipoprotein cholesterol in patients with rheumatoid arthritis. Annals of the Rheumatic Disease. 2003; 62: 842–845. PMID: 12922956

46. Costa-Silva JH, Lyra MM, Lima CR, Arruda VM, Araújo AV, Ribeiro AR, Wanderley AG. A toxicological evaluation of the effect of Carapa guianensis Aublet on pregnancy in Wistar rats. Journal of Ethnopharmacology. 2007; 112(1): 122-1. http//doi.org/10.1016/j.jep.2007.02.004

47. Santos TM, Sinzato YK, Gallego FQ, Lessi IL, Volpato GT, Dallaqua B, Damasceno DC. Extracellular HSP70 levels in diabetic environment in rats. Cell Stress Chaperones. 2015; 20: 595–603. https://doi.org/10.1007/s12192-015-0581-4

48. Soares TS, Andreolla AP, Miranda CA, Klöppel E, Rodrigues LS, Moraes-Souza RQ, Damasceno DC, Volpato GT, Campos KE. Effect of the induction of trans-generational obesity on maternal-fetal parameters. Systems Biology Reproductive Medicine. 2018; 64:51–59. https://doi.org/10.1080/19396368.2017.1410866

49. Karcz K and Królak-Olejnik B. Impact of Gestational Diabetes Mellitus on Fetal Growth and Nutritional Status in Newborns. Nutrients. 2024; 16:4093. https:// doi.org/10.3390/nu16234093

50. Parrettini S, Caroli A and Torlone E. Nutrition and metabolic adaptations in physiological and complicated pregnancy: Focus on obesity and gestational diabetes. Frontiers in Endocrinology. 2020;11:611929. doi: 10.3389/fendo.2020.611929

51. Al-Attar AM, Alsalmi FA. Effect of *Olea europaea* leaves extract on streptozotocin induced diabetes in male albino rats. Saudi Journal of Biological Sciences. 2017. https://doi.org/10.1016/j.sjbs.2017.03.002

52. Mohammed H, Okail H, Ibrahim M, Emam N. Influences of olive leaf extract in the kidney of diabetic pregnant mice and their offspring. The Journal of Basic and Applied Zoology. 2018; 79:3. http//doi.org/10.1186/s41936-018-0024-8

53. Cruz LL, Silva BSF, Araujo GG, Leal-Silva T, Paula VG, Souza MR, Soares TS, Moraes-Souza RQ, Monteiro GC, Lima GPP, Damasceno DC, Volpato GT. Phytochemical and antidiabetic analysis of Curatella americana L. aqueous extract on the rat pregnancy. Journal of Ethnopharmacology. 2022; 293:115287. https://doi.org/10.1016/j.jep.2022.115287

54. Giudetti AM. Editorial: Lipid metabolism in obesity. Frontiers in Physiology. 2023; 14: 1268288. Doi:10.3389/fphys.2023.1268288

55. Seydoux J, Chinet A, Schneider-Picard G, Bas S, Imesch E, Jeannet FA, Girardier L. Brown Adipose Tissue Metabolism in Streptozotocin-Diabetic Rats. Endocrinology.1983; 113(2): 604–610. https://doi.org/10.1210/endo-113-2-604

56. Afiune LAF, Leal-Silva T, Sinzato YK, Moraes-Souza RQ, Soares TS, Campos KE, Fujiwara RT, Herrera E, Damasceno DC, Volpato GT. Beneficial effects of *Hibiscus rosa-sinensis* L. flower aqueous extract in pregnant rats with diabetes. PLoS ONE. 2017; 12(6): e0179785. https://doi.org/10.1371/journal.pone.0179785.

57. Eze VN and Chioma N. Effects of aqueous extract of bitter kola (*Garcinia kola*) on the pregnancy outcome and early postnatal development of the offsprings of diabetic pregnant rats. CARD International Journal of Medical Science and Applied Biosciences (IJMSAB). 2017; 2(3): 59-68. http://www.casirmediapublishing.com

58. ADA (American Diabetes Association). Management of diabetes in pregnancy: Standards of Medical Care in Diabetes—2020. Diabetes Care. 2020; 43(1): S183–S192. https://doi.org/10.2337/dc20-S014

59. Vambergue A, Valat AS, Dufour P, Cazaubiel M, Fontaine P, Puech F. Physiopathology of diabetes gestational. Journal Gynecology Obstetrical Biological Reproduction. 2002; 31(6): 4S3-4S10. PMID: 12451352.

60. Lokman EF, Saparuddin F, Muhammad H, Omar MH, Zulkapli A. *Orthosiphon stamineus* as a potential antidiabetic drug in maternal hyperglycemia in streptozotocin-induced diabetic rats. Integrative Medicine Research. 2019; 8: 173–179. http//doi.org/ 10.1016/j.imr.2019.05.006

61. Viroel FJM, Laurino LF, Caetano ÉLA, Jozala AF, Spim SRV, Pickler TB, Sercundes MK, Gomes MC, Hataka A, Grotto D, Gerenutti M. Ganoderma lucidum Modulates

Glucose, Lipid Peroxidation and Hepatic Metabolism in Streptozotocin-Induced Diabetic Pregnant Rats. Antioxidants. 2022; 11: 1035. https://doi.org/10.3390/ antiox11061035

62. Lopez G, Wysocka W, Maizitaigui B, Alzugaray ME, Del Zotto H, Borelli MI. Quinolizidine alkaloids isolated from Lupinus species enhance insulin secretion. European Journal of Pharmacology.2004; 504 (1-4): 139- 142. http//doi.org/ 10.1016/j.ejphar.2004.09.008

63. Fang XK, Gao Y, Yang HY, Lang SM, Wang QJ, Yu BY, Zhu DN. Alleviating effects of active fraction of Euonymus alatus abundant in flavonoids on diabetic mice. American Journal of Chinese Medicine. 2008; 36 (1): 125-140. http//doi.org/ 10.1142/S0192415X08005643

64. Deepa P, Sowndhararajan K, Kim S, Park SJ. A role of *Ficus* species in the management of diabetes mellitus: A review. Journal of Ethnopharmacology. 2018; 215: 210–232. http//doi.org/10.1016/j.jep.2017.12.045.

65. Coskun O, Kanter M, Korkmaz A, Oter S. Quercetin, a flavonoid antioxidant, prevents and protects streptozotocin-induced oxidative stress and beta-cell damage in rat pancreas. Pharmacological Research. 2005; 51: 117-123. http//doi.org/10.1016/j.phrs.2004.06.002.

66. Ikemoto T, Kazunori FT, Wada Y, Gao L, MD, Miyazaki K, Yamada S, Saito Y, Imura S, Morine Y, Shimada M. Adipose Tissue from Type 1 Diabetes Mellitus Patients Can Be Used to Generate Insulin-Producing Cells. Pancreas. 2020; 49 (9): 1225–1231. http//doi.org/ 10.1097/MPA.0000000000001663.

67. Suarez M, Kattah A, Grande J, Garovic V. Renal disorders in pregnancy: Core curriculum. Am Journal Kidney Disease.2019;73(1):119–130. https://doi.org/10.1053/j.ajkd.2018.06.006

68. Lata I. Hepatobiliary diseases during pregnancy and their management: an update. International Journal Critical Illness and Injury Science. 2013; 3:175–82.

http//doi.org/ 10.4103/2229-5151.119196

69. Salahi P, Rocky A, Dezfoulian O, Azizi A, Alirezaei M. Betaine alleviated hepatic and renal injury in diabetic pregnant rats: biochemical and histopathological evidences. Journal of Diabetes & Metabolic Disorders. 2020; 9p. https://doi.org/10.1007/s40200-020-00572-7

70. Ribeiro-Filho J, Carvalho-Leite F, Surrage-Calheiros A, de Brito Carneiro A, Alves Azeredo, J, Fernandes de Assis E, da Silva Dias C, Regina Piuvezam MT, Bozza P, Curine. Inhibits Macrophage Activation and Neutrophil Recruitment in a Mouse Model of Lipopolysaccharide-Induced Inflammation. Toxins. 2019: 11(12): 705. http//doi.org/ 10.3390/toxins11120705

71. Nguenang GS, Ntyam ASM, Kuete V. Acute and Subacute Toxicity Profiles of the Methanol Extract of *Lycopersicon esculentum* L. Leaves (Tomato), a Botanical with Promising *In Vitro* Anticancer Potentialial. Evidence-Based Complementary and Alternative Medicine. 2020; V2020:1-10. http//doi.org/ 10.1155/2020/8935897

72. Regnell SE, Lernmark A. Hepatic Steatosis in Type 1 Diabetes. The Review of Diabetic Studies. 2011; 8(4): 454–467. http//doi.org/10.1900/rds.2011.8.454

73. Herrera E, Amusquivar E. Lipid metabolism in the fetus and the newborn. Diabetes Metabolism Research and Reviews. 2000; 16: 202–210. http//doi.org//10.1002/1520-7560(200005/06)16:3<202::aid-dmrr116>3.0.co;2-#

74. Mulder JWCM, Kusters DM, van Lennep JER, Hutten BA. Lipid metabolism during pregnancy: consequences for mother and child. Current Opinion in Lipidology. 2024; 35(3): 133–140. http//doi.org/10.1097/MOL.0000000000000927

75. Sundaram EN, Uma Maheswara Reddy P, Singh KP. Effect of Alcoholic Extracts of Indian Medicinal Plants on the Altered Enzymatic Activities of Diabetic Rats. Indian

Journal of Pharmaceutical Sciences. 2009; 71(5): 595-598. http//doi.org/ 10.4103/0250-474X.58175

76. Al-Jaghthmi OHA, Zeid IEMEA. Hypoglycemic and hepatoprotective effect of Rhizophora mucronata and Avicennia marina against streptozotocin-induced diabetes in male rats. Journal of Advanced Veterinary and Animal Research.2020; 7(1): 177-185. http://doi.org/10.5455/javar.2020.g408

77. Vinturache A, Khalil A.. *Chapter :* Maternal Physiological Changes in Pregnancy ; in : The Continuous Textbook of Women's Medicine Series – Obstetrics Module -Volume 4 - Fetal development and maternal adaptation ; Editor : Khalil A ; Edition Glob Libr Women's Med. 2021; 1-26. (GLOWMISSN: 1756-2228; DOI 10.3843/GLOWM.411323)

78. Stefan N, Sun Q, Fritsche A, Machann J, Schick F, Gerst, F, Jeppesen C, Joost H, Hu FB, Boeing H, Ullrich S, Häring H, Schulze MB. Impact of the Adipokine Adiponectin and the Hepatokine Fetuin-A on the Development of Type 2 Diabetes: Prospective Cohort- and Cross-Sectional Phenotyping Studies. *PLoS ONE.* 2014; 9(3): e92238. http//doi.org/10.1371/journal.pone.0092238

79. Trammel JC, Sapra A. Physiology, Systemic vascular Resistance. StatPearls. 2023. www.ncbi.nlm.nih.gov>books>NBK556075.

80. Domon A, Katayama K, Sato T, Tochigi Y, Tazaki H and Suzuki H. Empagliflozin ameliorates symptoms of diabetic and renal tubular dysfunction in a rat model of diabetes with enlarged kidney (DEK). PLoS ONE. 2021; 16(5): e0251135. https://doi.org/10.1371/journal.pone.0251135.

81. Morozova S, Suc-Royer I, Auwerx J. Modulateurs du métabolisme du cholestérol et avenir du traitement de l'athérosclérose. Médecine/Sciences. 2004; 20(6-7) : 685-90. http://dx.doi.org/10.1051/medsci/2004206-7685

82. Bequer L, Gómez T, Molina JL, Álvarez A, Chaviano C, Clapés S. Experimental diabetes impairs maternal reproductive performance in pregnant Wistar rats and their offspring. Systems Biology in Reproductive Medicine. 2018; 64(1): 60-70. http//doi.org//10.1080/ 19396368. 2017; 1395928.

83. Gallego FQ, Sinzato YK, Miranda CA, Iessia IL, Dallaqua B, Volpato GT, Scaranod WR, SanMartíne S, Damasceno DC. Pancreatic islet response to diabetes during pregnancy in rats. Life Sciences. 2018; 214: 1–10. https://doi.org / 10.1016 / j.lfs. 2018. 10.046

84. Bueno A, Sinzato YK, Volpato GT, Gallego FQ, Perecin F, Rodrigues T, Bueno, A., Damasceno, D. C. Severity of prepregnancy diabetes on the fetal malformations and viability associated with early embryos in rats. *Biol. Reprod*. 2020; 103: 938–950. http//doi.org/10.1093/biolre/ioaa151

85. Vitti M, Di Emidio G, Di Carlo M, Carta G, Antonosante A, Artini PG, Cimini A, Tatone C, Benedetti E. Peroxisome Proliferator-Activated Receptors in Female Reproduction and Fertility. *PPAR Research*. 2016; 2016: 4612306. http// doi.org/10.1155/2016/4612306

86. Desforges M, Sibley C. Placental nutrient supply and fetal development. The International Journal of Developmental Biology. 2010; 54: 377–390. https://doi.org/10.1387/ijdb.082765md

87. Cornélis F. Intérêt de l'examen anatomopathologique du placenta. Revue Francophone Des Laboratoires. 2008;(402): 71–76. https://doi.org/10.1016/S1773-035X(08)71786-6

88. Bendix I, Miller SL, & Winterhager E. Editorial: Causes and Consequences of Intrauterine Growth Restriction. Frontiers in Endocrinology. 2020; 11: 1-3. http//doi.org/10.3389/fendo.2020.00205

89. Cruz LL, Silva BSF, Araujo GG, Leal-Silva T, Paula VG, Souza MR, Soares TS, Moraes-Souza RQ, Monteiro GC, Lima GPP, Damasceno DC, Volpato GT. Phytochemical and

antidiabetic analysis of Curatella americana L. aqueous extract on the rat pregnancy. Journal of Ethnopharmacology. 2022; 293:115287. https://doi.org/10.1016/j.jep.2022.115287

90. Sun B, Yan H, Li C, Yin L, Li F, Zhou L, Han X. Beneficial effects of walnut (*Juglans regia* L.) oil-derived polyunsaturated fatty acid prevents a prooxidant status and hyperlipidemia in pregnant rats with diabetes. Nutrition and Metabolism (Lond). 2020; 17:92. https://doi.org/10.1186/s12986-020-00514-3

91. Masunda TA, Mbala MB, Kayembe SJ, Longoma BF, Ngbolua KN, Tshibangu DST, et Mpiana PT. Activité anti-hyperglycémique et antiradicalaire des extraits des fruits de *Raphia gentiliana* De Wild. (Arecaceae). International Journal of Biological and Chemical Sciences. 2014; 8(6): 2441-2451.

Click here to access/download;Figure;Fig1.tif.tif

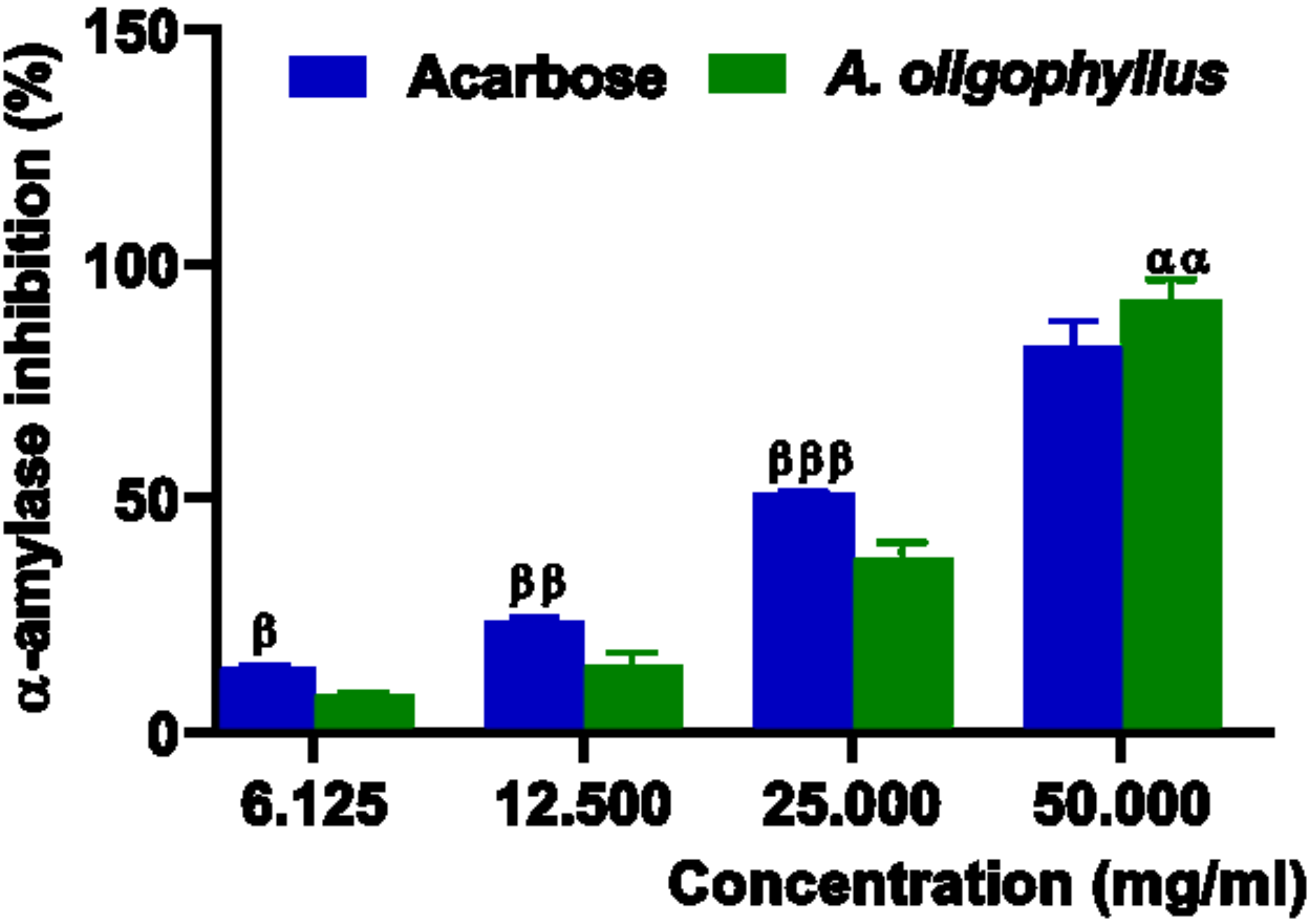

Fig2A.tif
Click here to access/download;Figure;Fig2A.tif.tif

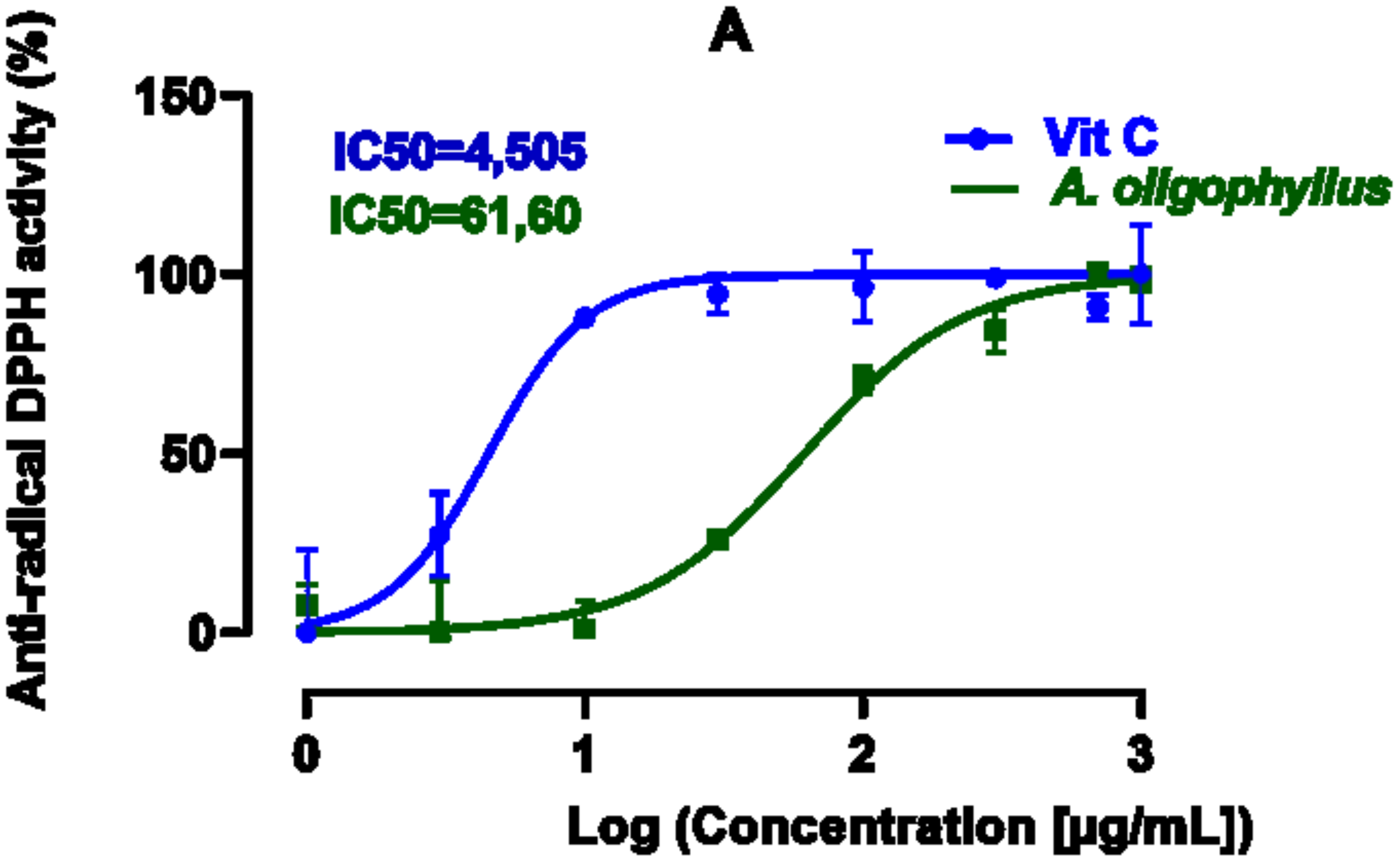

Fig2B.tif

Click here to access/download;Figure;Fig2B.tif.tif

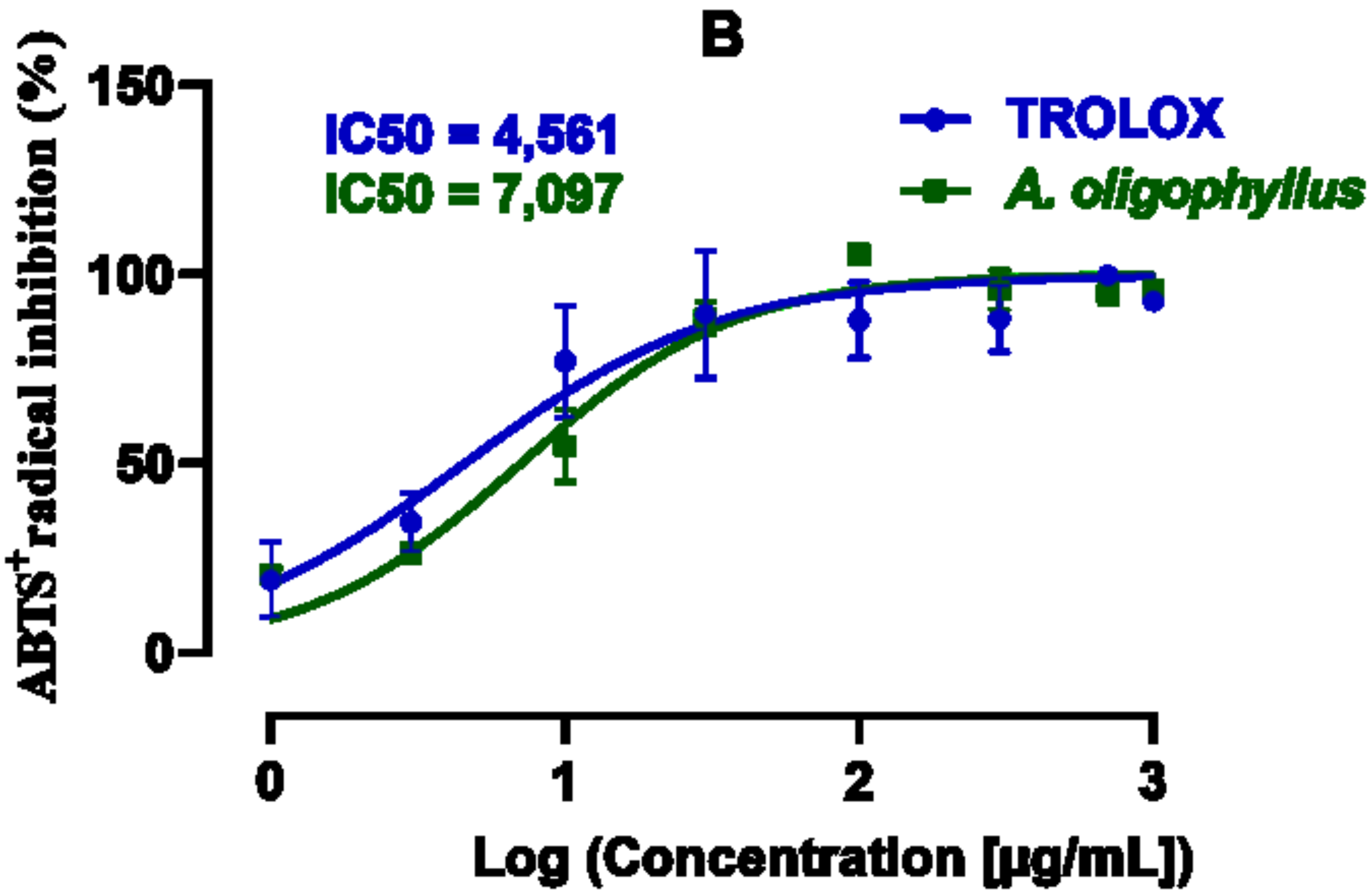

Fig2C.tif Click here to access/download;Figure;Fig2C.tif.tif

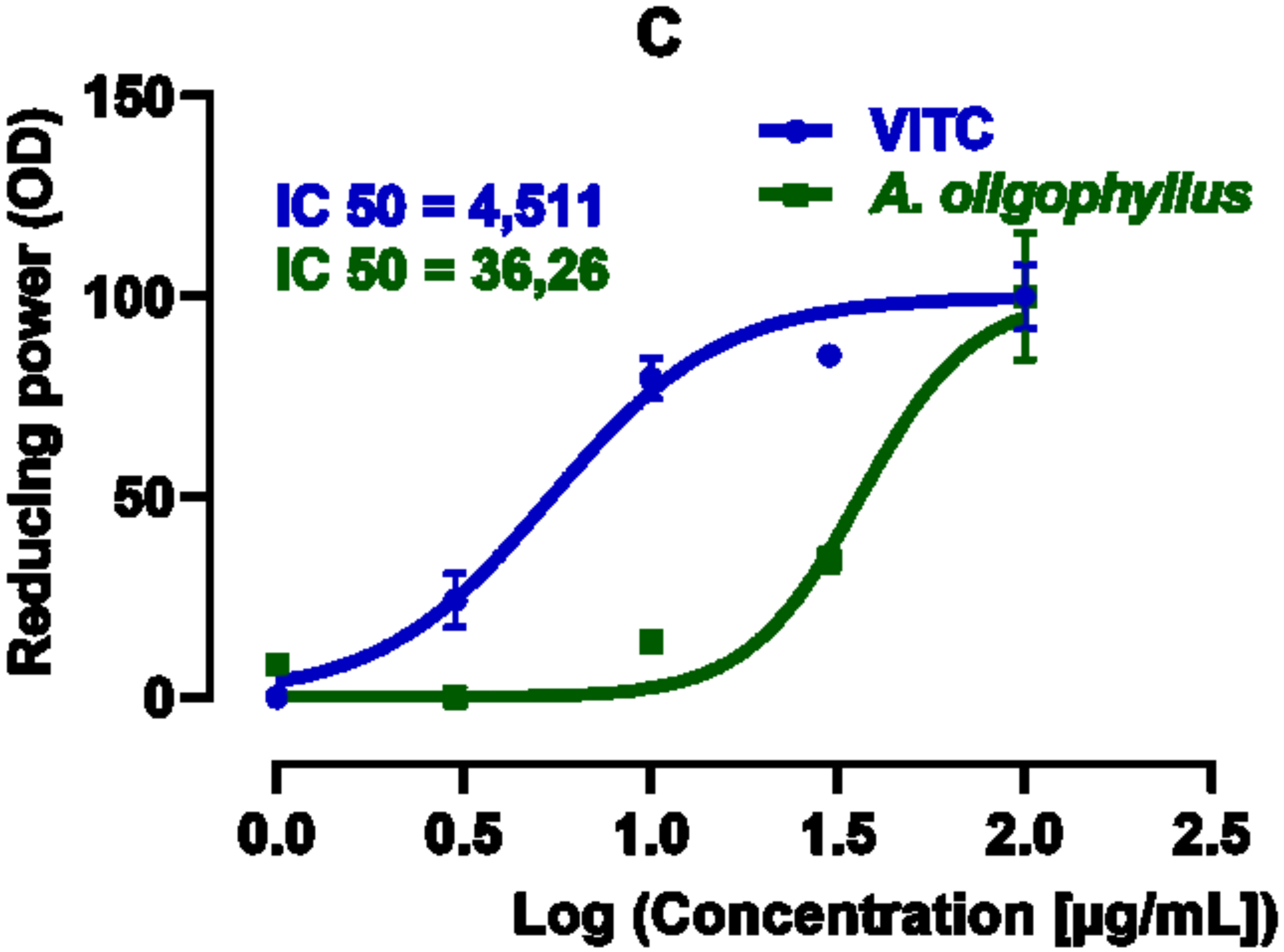

Fig2D.tif

Click here to access/download;Figure;Fig2D.tif.tif

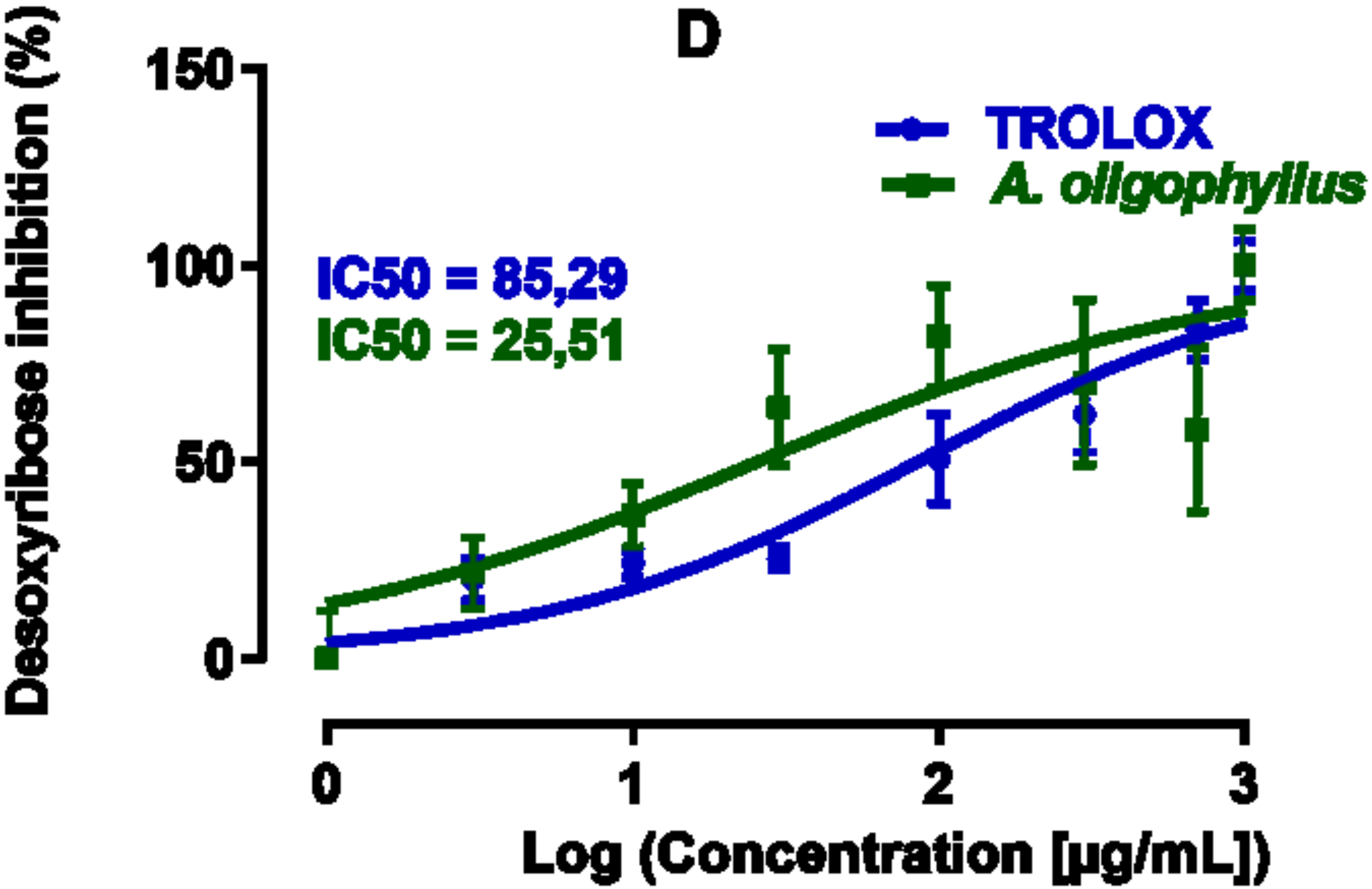

Fig3.tif
Click here to access/download;Figure;Fig3.tif.tif

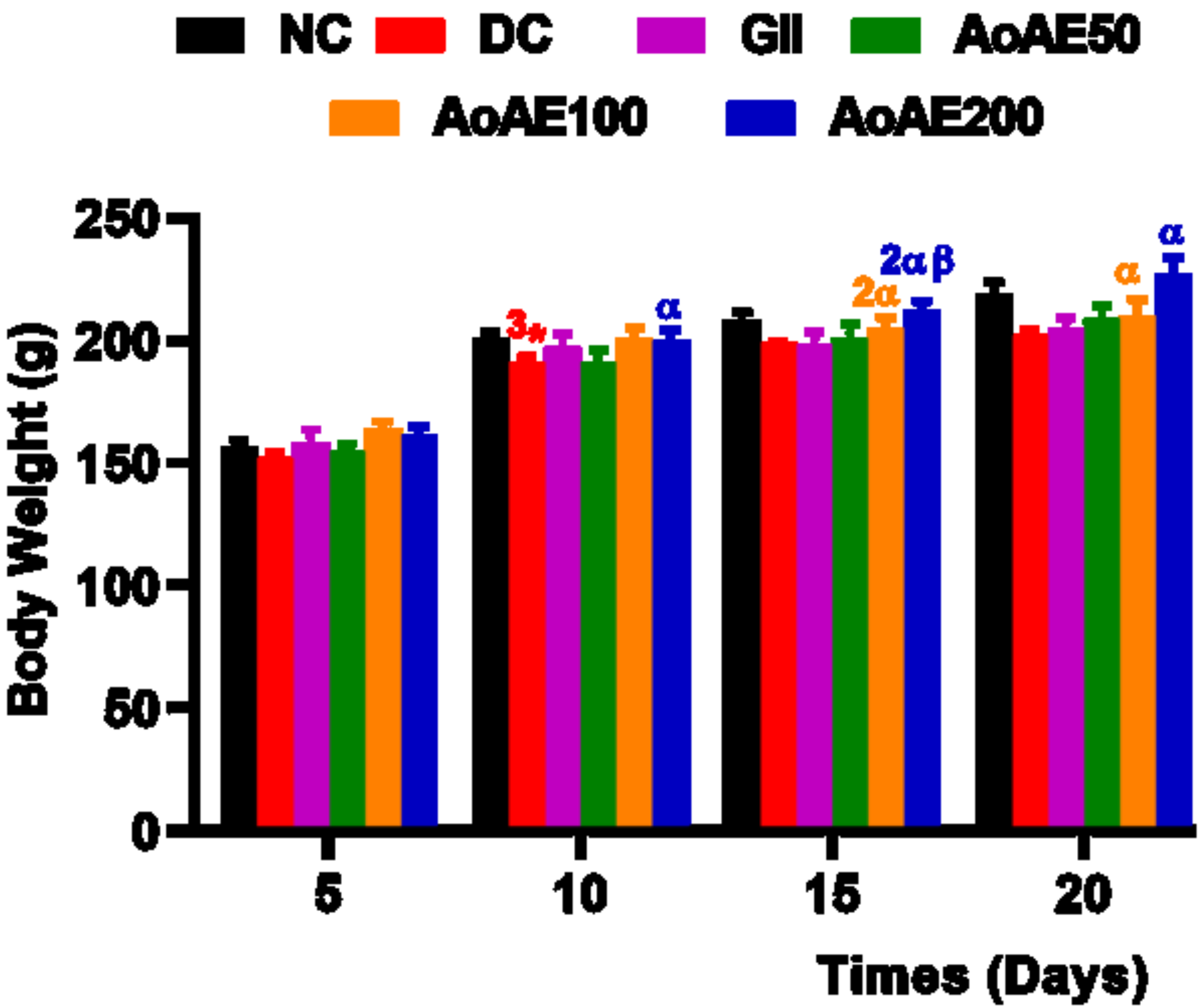

Click here to access/download;Figure;Fig4A.tif.tif

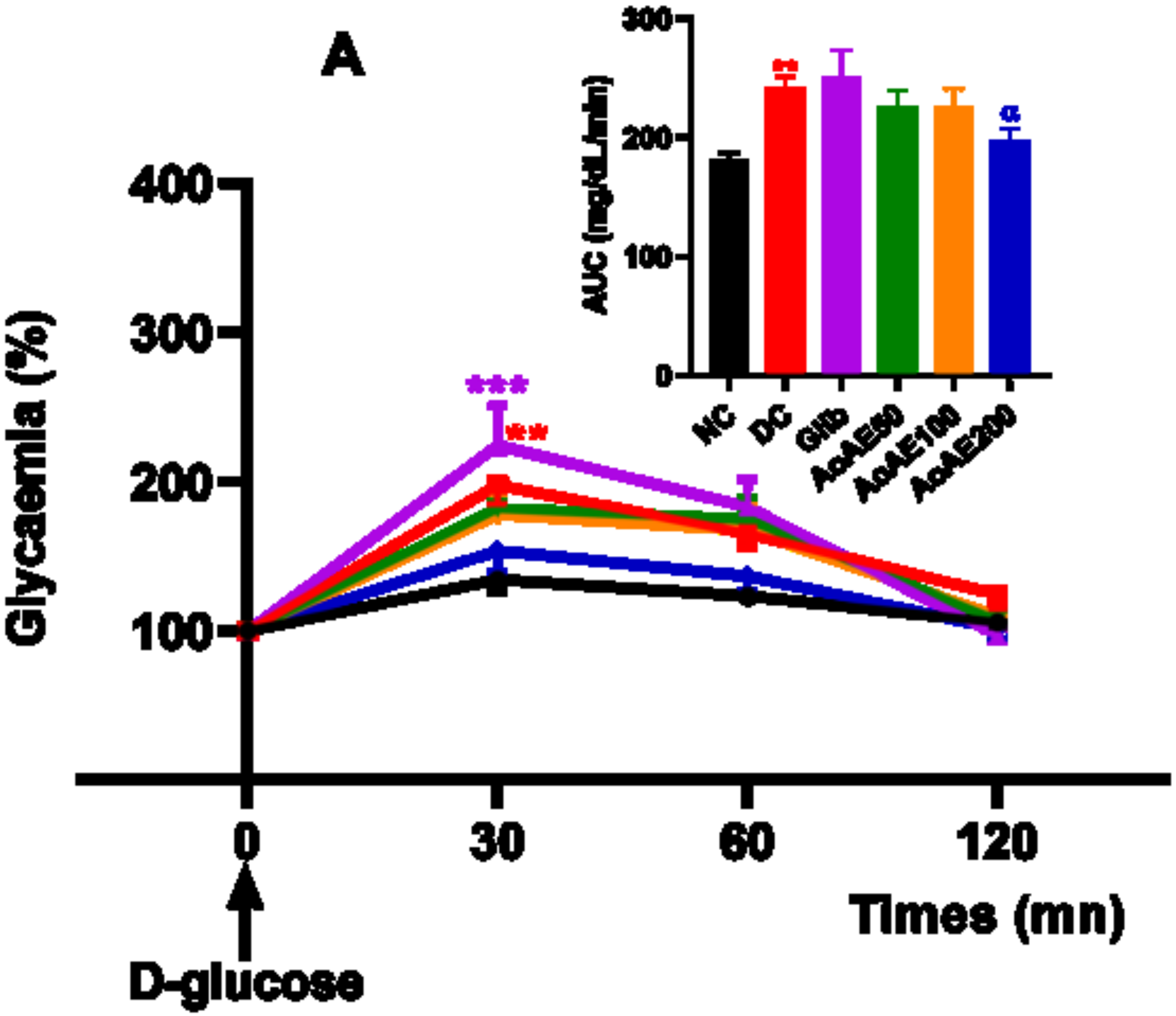

Click here to access/download;Figure;Fig4B.tif.tif

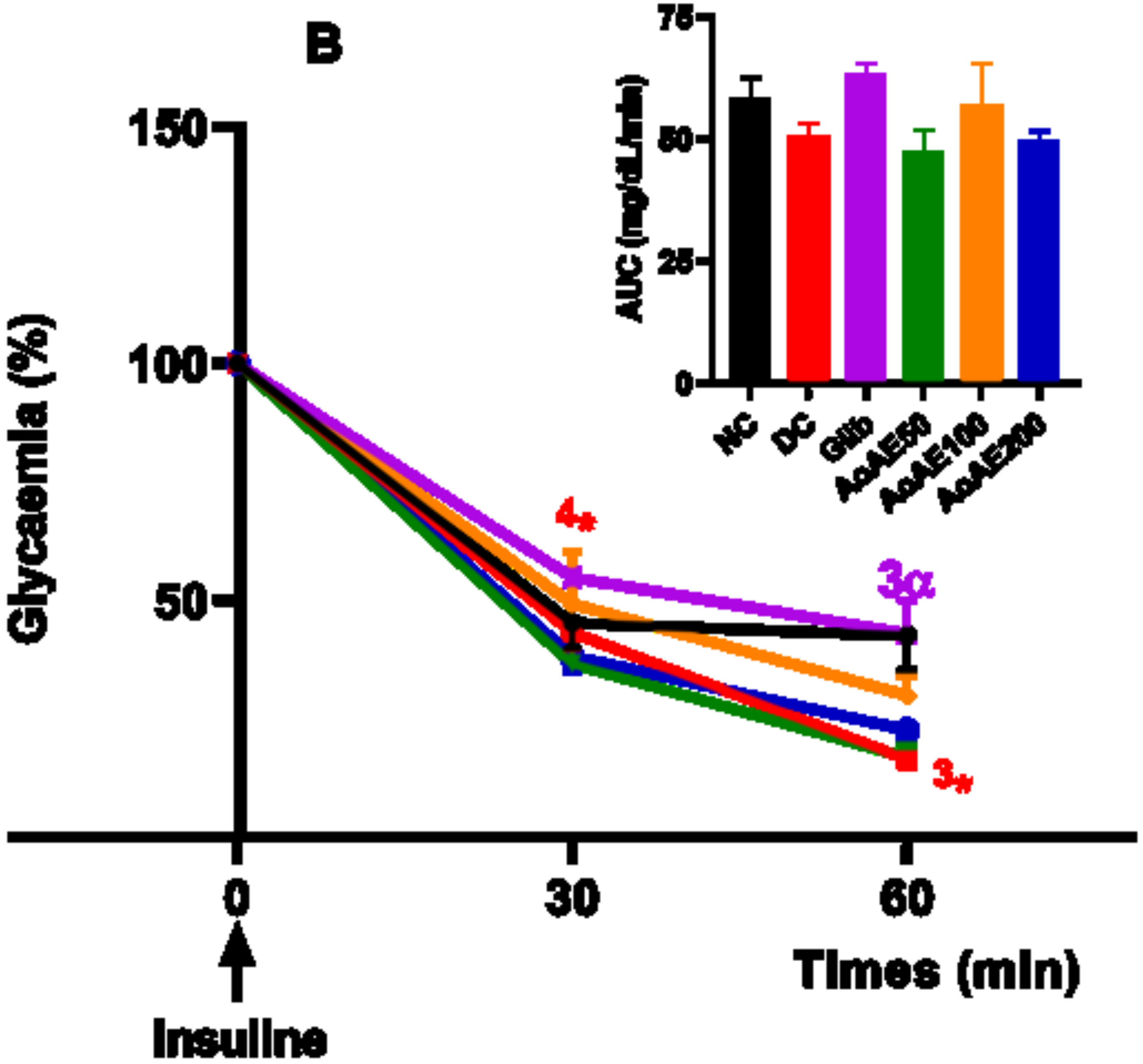

Fig5A.tif Click here to access/download;Figure;Fig5A.tif.tif

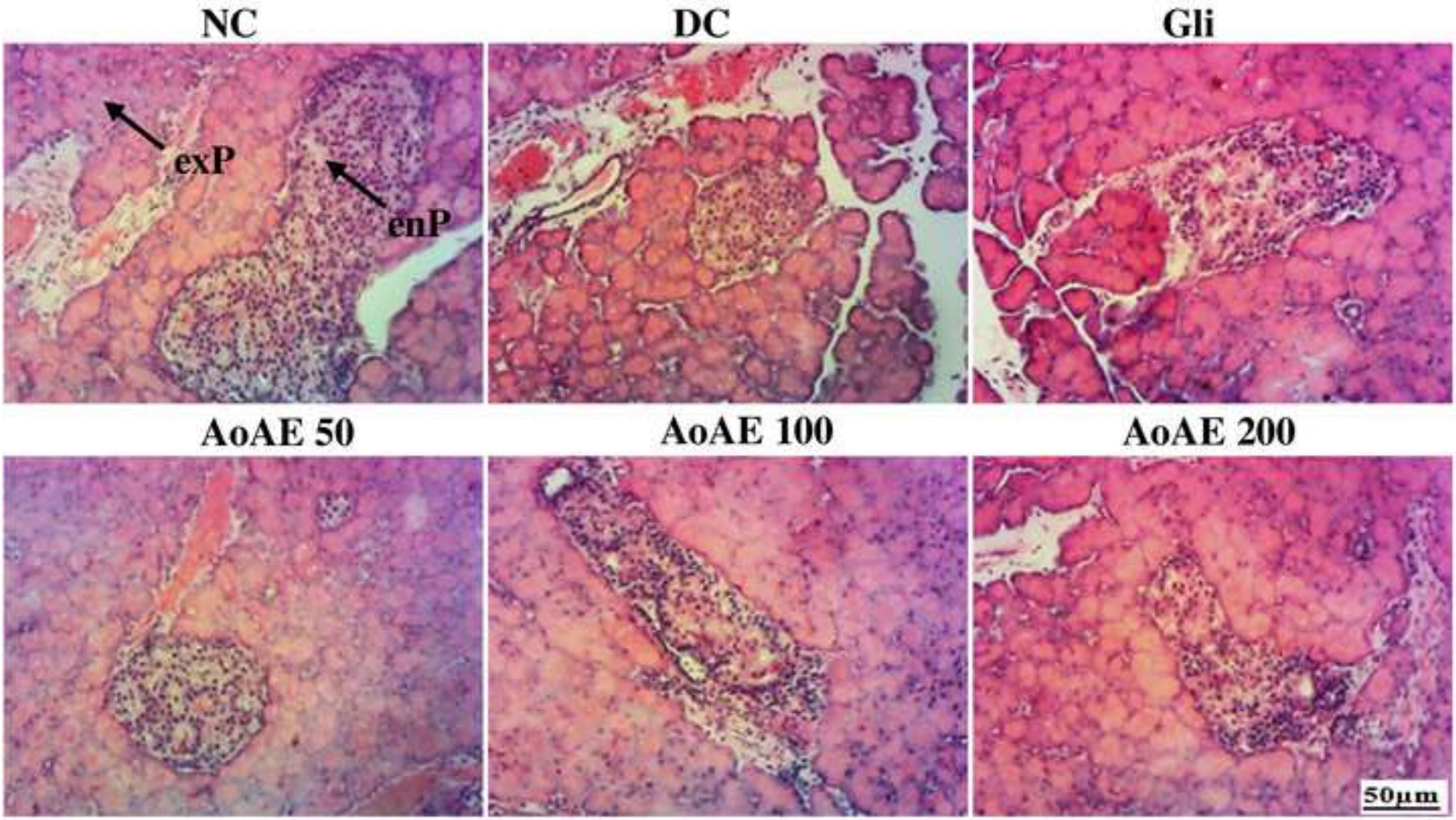

Fig5B.tif

Click here to access/download;Figure;Fig5B.tif.tif

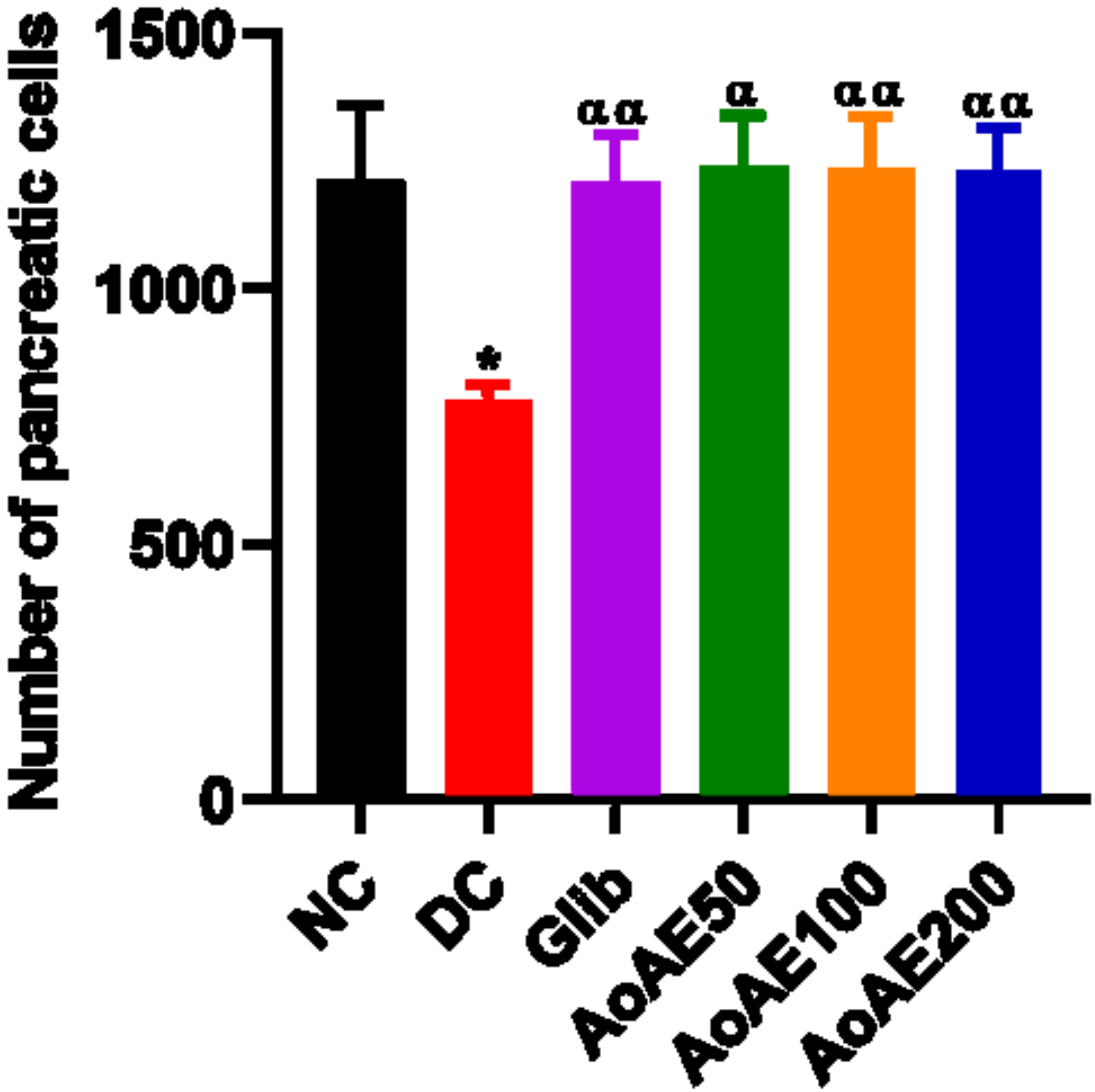

Fig6A.tif Click here to access/download;Figure;Fig6A.tif.tif

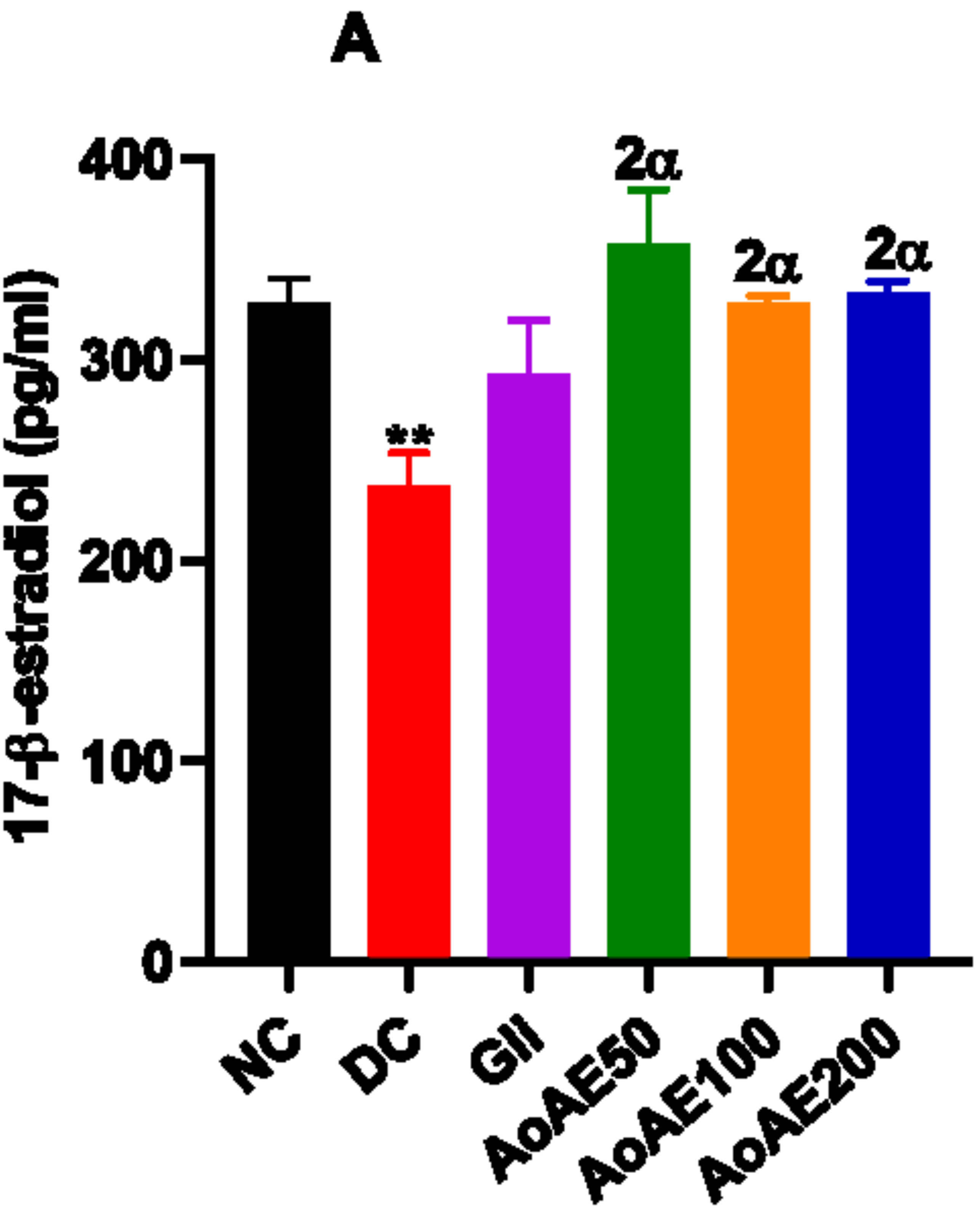

Fig6B.tif Click here to access/download;Figure;Fig6B.tif.tif

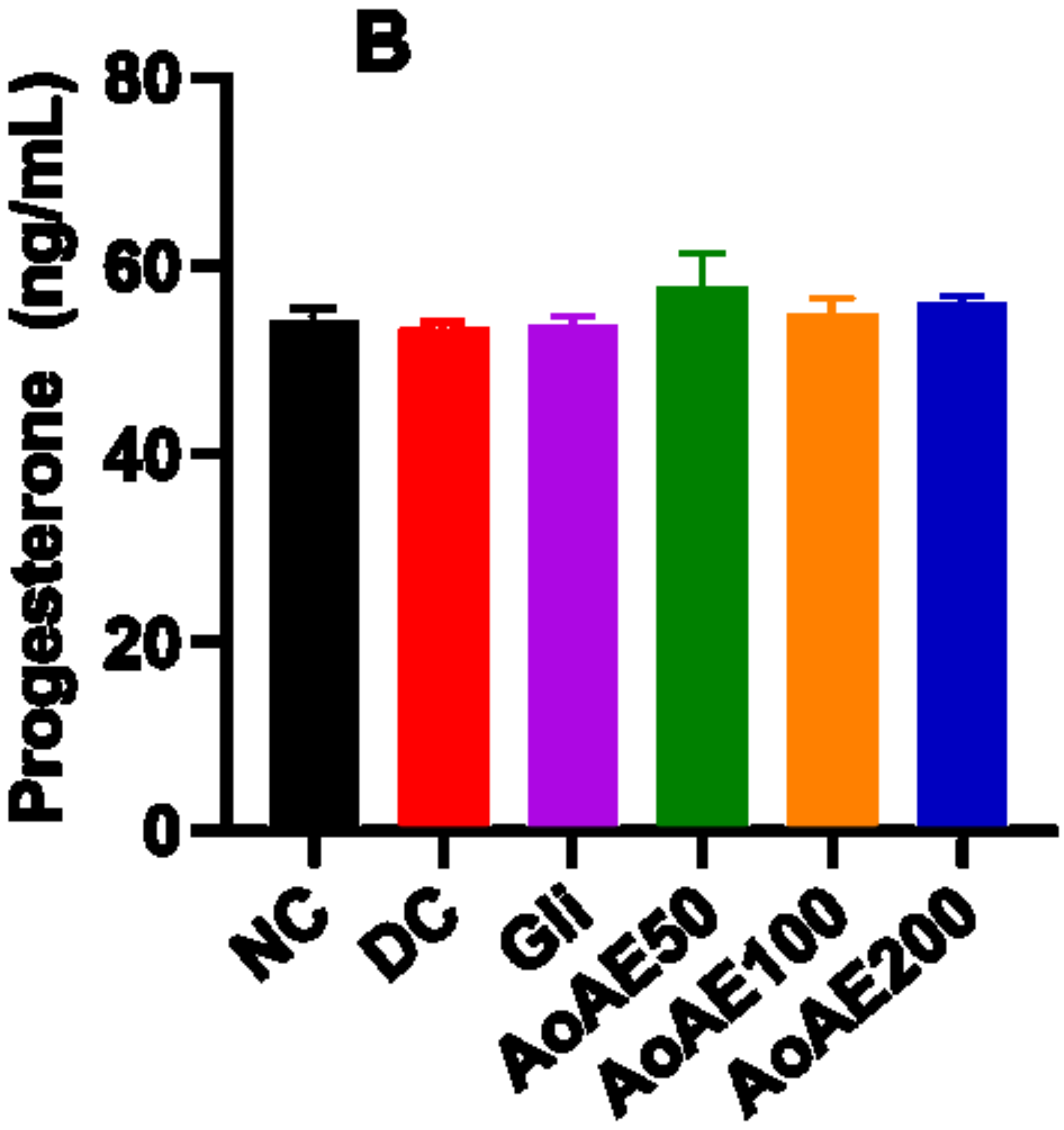

Fig7.tif
Click here to access/download;Figure;Fig7.tif.tif

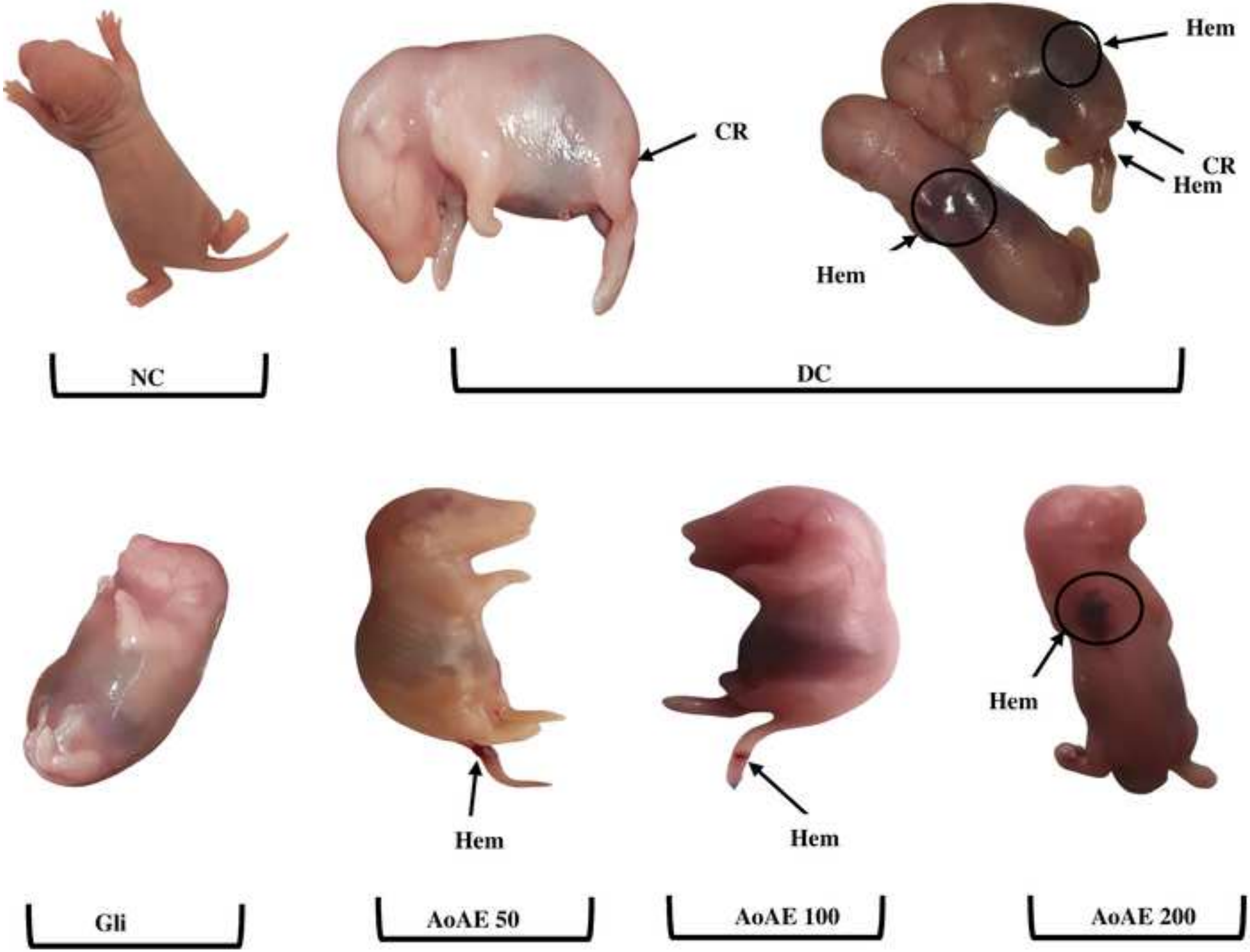

Click here to access/download;Figure;Fig8.tif.tif

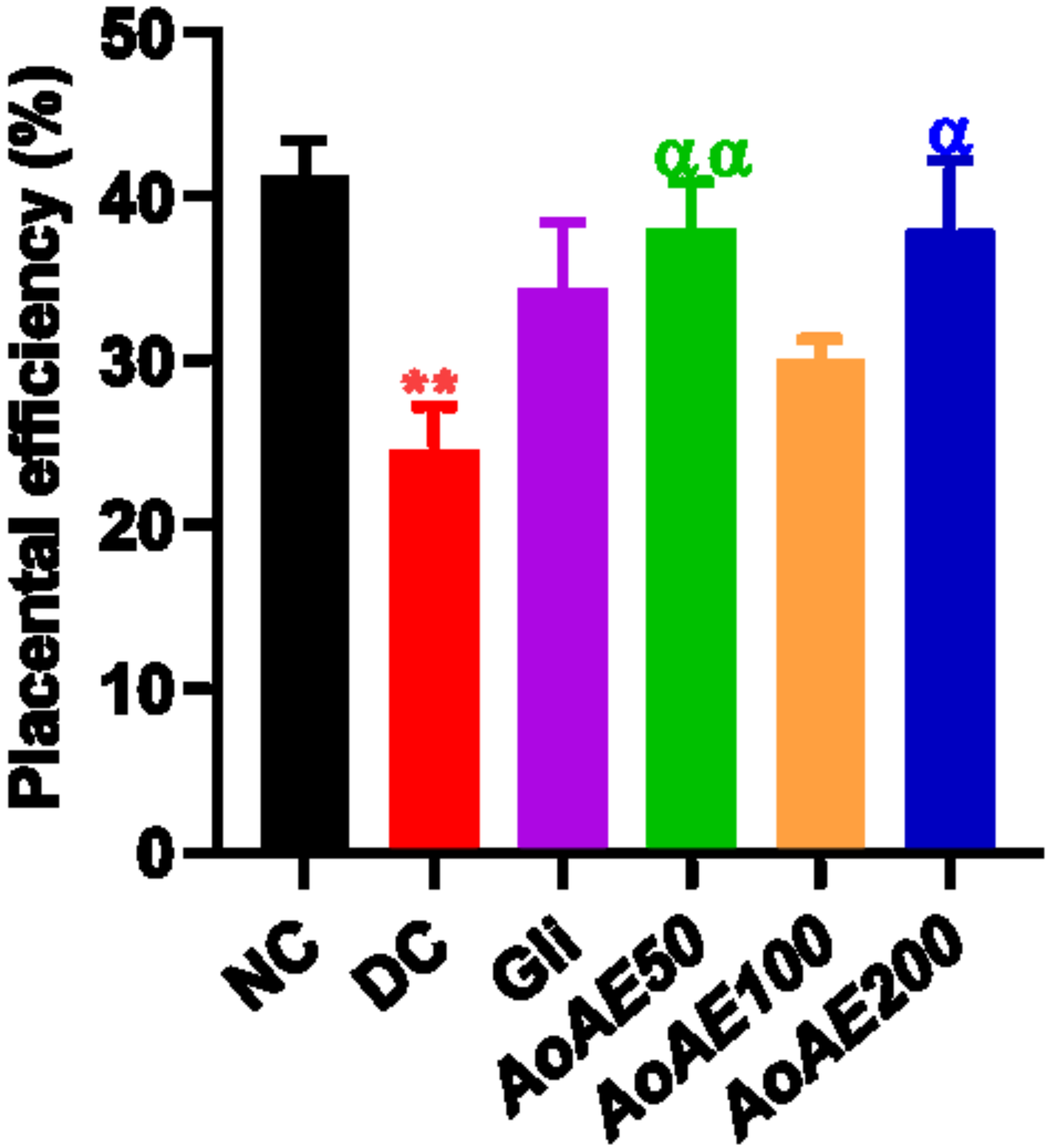

Fig9.tif

Click here to access/download;Figure;Fig9.tif.tif

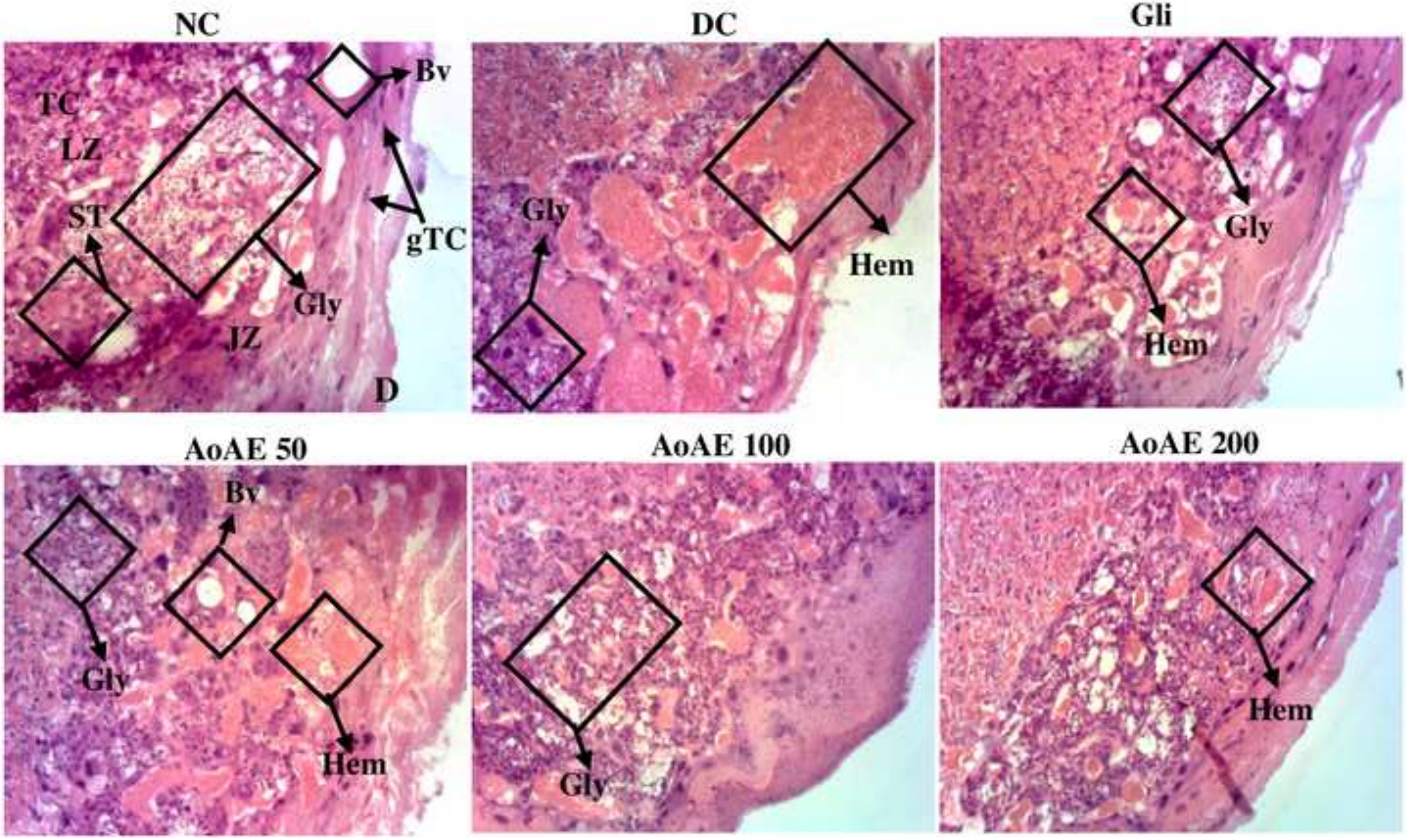

Supplement: Attachment — Submitted filename: 1-PONE-D-25-21592.pdf [file pone.0334166.s002.pdf]
